# Integrins and tetraspanins mediate CD36-actin coupling and regulate CD36 organization and signaling

**DOI:** 10.1083/jcb.202504156

**Published:** 2026-08-21

**Authors:** Soma Jana, Huong-Tra Ngo, Han Huang, Jaime Guerrero, Jesus Vega-Lugo, Nicolas Touret, Aparajita Dasgupta, Khuloud Jaqaman

**Affiliations:** 1 Department of Biophysics, UT Southwestern Medical Center, Dallas, TX, USA; 2 Department of Biochemistry, University of Alberta, Edmonton, Canada; 3 Lyda Hill Department of Bioinformatics, UT Southwestern Medical Center, Dallas, TX, USA

## Abstract

Submembrane cortical actin (CA) plays a large role in regulating the dynamic organization of cell surface receptors, which in turn regulates receptor signaling. Many receptors have short intracellular domains and no known link to actin. Here, we identified the β_1_-integrin subunit and several tetraspanins (CD9, CD81, and CD151) as part of the hitherto unknown molecular link between the receptor CD36 and CA. Our data indicate that CD36 in vascular endothelial cells interacts with these proteins, with stronger interactions near the cell edge. Compromising these interactions via the point mutation G12V in the N-terminal transmembrane domain of CD36 alters the dynamic organization of CD36 on the cell surface and weakens its coupling to CA dynamics. Moreover, it abolishes thrombospondin-1–induced CD36 signaling through the Src family kinase Fyn. Given their many interactions with transmembrane proteins, tetraspanins and integrins may provide a ubiquitous mechanism for plasma membrane–CA coupling.

## Teaser

Jana, Ngo et al. reveal that β1-integrin and the tetraspanins CD9, CD81, and CD151 link the surface receptor CD36 to cortical actin, thereby regulating its dynamic organization and signaling. With their many interactions, integrins and tetraspanins may link many receptors lacking actin-binding motifs to cortical actin.

## Introduction

CD36 is an integral membrane protein with a large extracellular domain and two short intracellular domains, expressed on the surface of many cell types, including microvascular endothelial cells (MVECs), macrophages, microglia, platelets, and various epithelia (Silverstein and Febbraio, 2009). It binds diverse ligands, e.g., thrombospondin-1 (TSP-1), oxidized low-density lipoprotein, fibrillar β-amyloid, and malaria-infected erythrocytes, and functions as a fatty acid transporter (Pepino et al., 2014; Silverstein and Febbraio, 2009). It is implicated in various physiological and pathological processes, such as angiogenesis, atherosclerosis, Alzheimer’s disease, immunity, diabetes, and obesity (Silverstein and Febbraio, 2009).

Like many receptors (Chen et al., 2022; Garcia-Parajo et al., 2014; Garcia-Parajo and Mayor, 2024; Goyette et al., 2019; O’Shea and Murray, 2008; Treanor et al., 2010; Wilson et al., 2011), CD36 signaling requires clustering and the formation of signaling complexes (Bamberger et al., 2003; Chen et al., 2022; Githaka et al., 2016; Heit et al., 2013; Jaqaman et al., 2011; Jiménez et al., 2000; McGilvray et al., 2000; Wilkinson et al., 2006; Wong et al., 2016). With its two short intracellular domains—7 and 13 amino acids—that lack signaling motifs and scaffolding domains, clustering and complex formation are critical to bring CD36 together with its signaling partners (Chen et al., 2022; Heit et al., 2013). In MVECs, CD36 clusters are enriched with Fyn (a Src family kinase; SFK), facilitating signaling upon ligand binding (Githaka et al., 2016).

The dynamic organization of CD36 at the plasma membrane (PM)—like that of many receptors (Garcia-Parajo et al., 2014; Garcia-Parajo and Mayor, 2024; Jaqaman and Grinstein, 2012) —depends on cortical actin (CA): perturbing actin reduces the dynamic interactions and clustering of CD36, and mutes signaling in response to ligand (Githaka et al., 2016; Jaqaman et al., 2011). In this work, the term CA encompasses the various actin cytoskeletal components within the submembrane cell cortex. It includes the barrier-forming CA meshwork, short dynamic CA filaments interspersed within the meshwork, and actin bundles/stress fibers interconnected with the meshwork, all of which have been implicated in influencing the organization and dynamics of PM constituents (Gowrishankar et al., 2012; Kusumi et al., 2012; Lehtimaki et al., 2021; Li et al., 2020; Svitkina, 2020; Vignaud et al., 2021). Yet the short intracellular domains of CD36 are unlikely to interact with CA directly. Rather, CA most likely exerts its influence on CD36 indirectly through other molecules (Freeman et al., 2018; Goswami et al., 2008; Gutiérrez-Martínez et al., 2023; Kusumi et al., 2012). In this respect, CD36 represents many PM components that are influenced by CA, but primarily indirectly through the action of other PM components.

The β_1_-integrin subunit and the tetraspanins CD9, CD81, and CD151 are strong candidates for mediating the molecular link between CD36 and CA. They physically interact with CD36, as shown via co-immunoprecipitation (co-IP) in various cell types, including MVECs (Heit et al., 2013; Huang et al., 2011; Kazerounian et al., 2011; Miao et al., 2001; Primo et al., 2005; Thorne et al., 2000). They also interact with each other (Berditchevski, 2001; Charrin et al., 2009; Yauch et al., 2000; Zhang et al., 2009) and with actin (Bailey et al., 2011; Coffey et al., 2009; Geiger et al., 2001; Kim et al., 2011; Sala-Valdés et al., 2006; Schmidt et al., 2024; Shigeta et al., 2003; Takeda et al., 2007; Zhang et al., 2009). Integrins and tetraspanins interact with many proteins at the PM (Charrin et al., 2009, 2014; Ivaska and Heino, 2011; Vicente-Manzanares and Sánchez-Madrid, 2018); thus, they likely play a ubiquitous role in PM-CA coupling.

Here, we tested the hypothesis that CD36 interactions with β_1_-integrin and tetraspanins mediate the link between CD36 and CA. Starting with the known physical interactions between CD36, β_1_-integrin, and the tetraspanins CD9, CD81, and CD151, we utilized advanced cellular imaging, quantitative image analysis, and point mutation of CD36 to (1) identify where at the PM and to what extent CD36 interacts with β_1_-integrin and these tetraspanins, (2) inhibit CD36 interactions with β_1_-integrin and tetraspanins, and (3) determine the consequences of inhibiting these interactions for CD36–CA coupling, the dynamic organization of CD36, and CD36 signaling.

## Results

### CD36 in MVECs colocalizes with β_1_-integrin and the tetraspanins CD9, CD81, and CD151, especially near the cell edge

**Figure S1.**
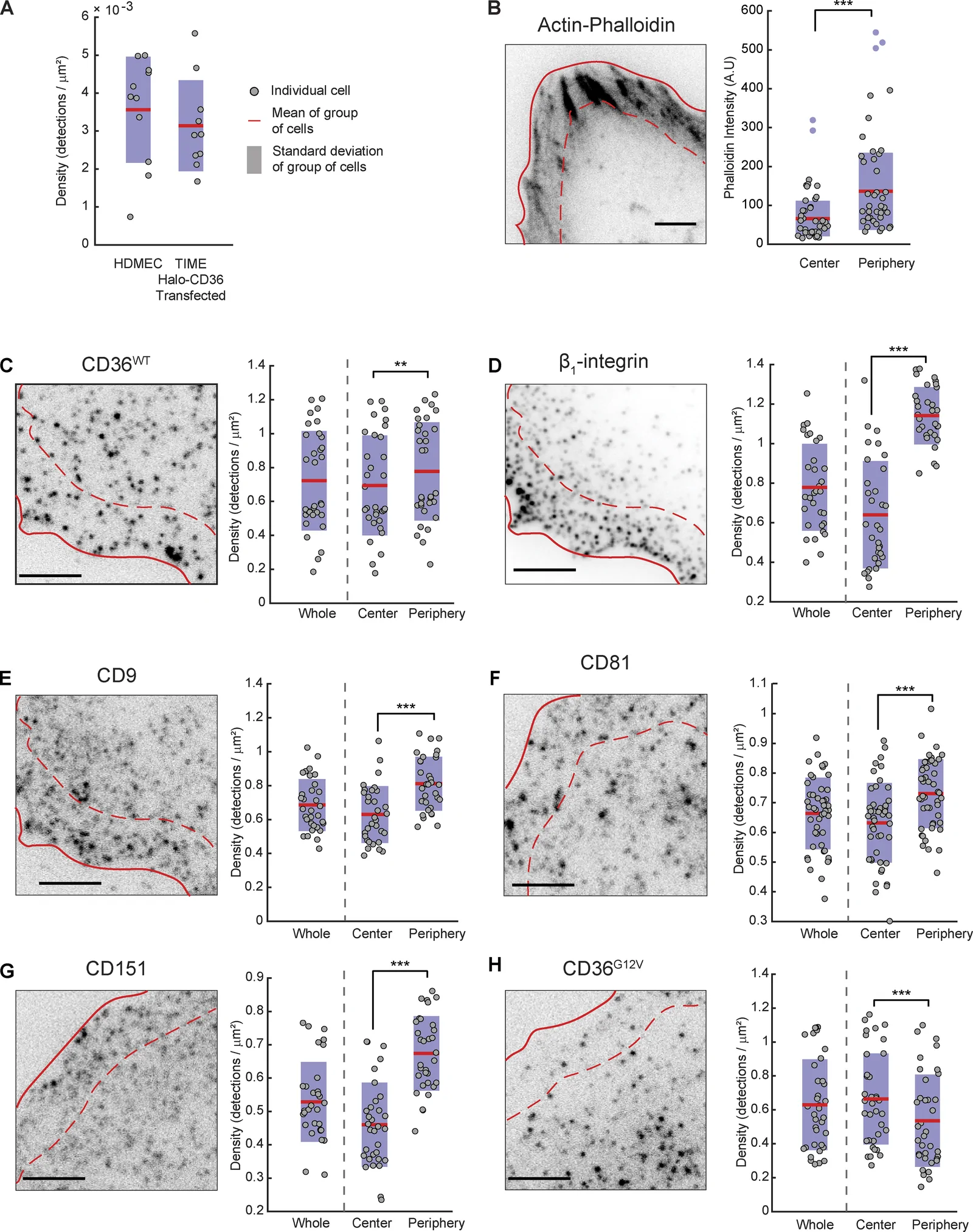
**CD36 expression level; density distributions of the various imaged molecules and actin in TIME cells. (A)** Quantification of CD36 detection density in HDMECs (primary MVECs) endogenously expressing CD36 (*n* = 11 cells) and in TIME cells transiently transfected with Halo-CD36^WT^ (*n* = 10 cells), in both cases as detected by the FA6-152 antibody against CD36. **(B)** Representative fixed-cell image of actin (stained using phalloidin) and quantification of its average intensity in the center and periphery regions separately. **(C–H)** Representative fixed-cell images of the indicated surface molecules and quantification of their detection densities in the whole imaged region, as well as in the center and periphery regions separately. In all images, solid red lines show the segmented cell edge; dashed lines specify the boundary between the periphery and center regions. Scale bars, 5 μm. The images shown in C, D, E, and H are, respectively, the same as the images shown in Fig. 1, B, C, D, and I. In all plots, circles, red lines, shaded bars, and asterisks are as in Fig 2. See Table 4 for number of cells, number of experimental repeats, and number of objects per channel per ROI used for analysis, except for A, where dataset size is indicated in the figure legend.

**Figure 1.**
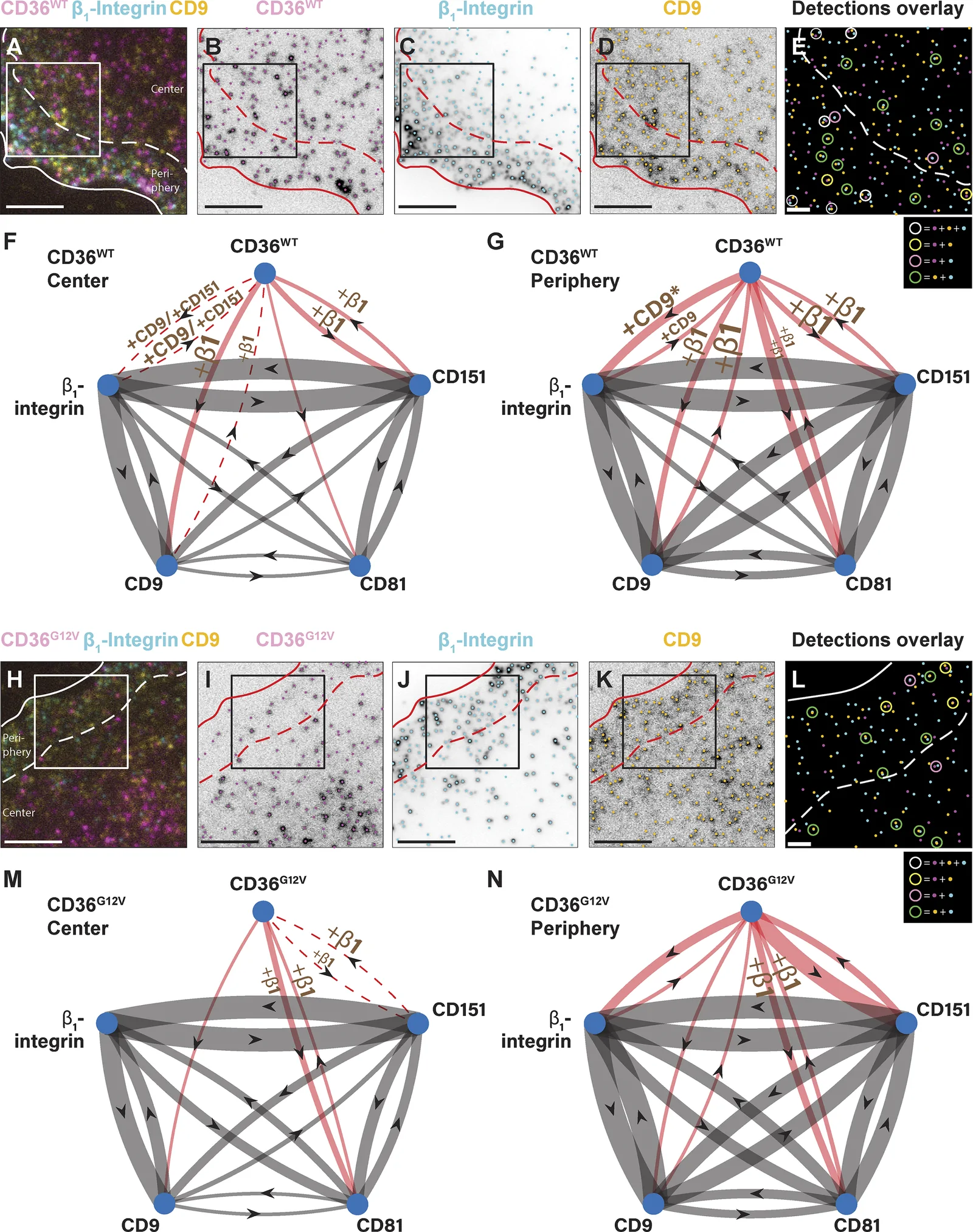
**G12V mutation weakens CD36 colocalization with β**_**1**_ **-integrin and CD9 and alters CD36 colocalization with CD81 and CD151 based on subcellular region (center vs. periphery). (A)** Representative three-color fixed-cell image of CD36 (magenta), β_1_-integrin (cyan), and CD9 (yellow) on the surface of a TIME cell expressing Halo-tagged CD36^WT^ and imaged via TIRFM. Scale bar, 5 μm. **(B–D)** Particle detections (shown as dots) overlaid on the individual channels. Scale bar, 5 μm. **(E)** Overlay of the three-channel detections for the boxed area in A–D, with color-coding following that in A–D. Circles highlight colocalization events between the different molecules, following color-coding in the legend at the bottom of the panel. Scale bar, 1 μm. In A–E, solid lines show the segmented cell edge; dashed lines specify the boundary between the periphery and the center. **(F and G)** Representation of the colocalization networks of CD36^WT^, β_1_-integrin, CD9, CD81, and CD151 in the center (F) and periphery (G) regions. Network arrows involving/not involving CD36 are shown in red/gray, for visual clarity. Arrows point from target molecule to reference molecule and represent the extent of target colocalization with reference. Solid arrows indicate significant target colocalization with reference (P value ≤ 0.05), with arrow thickness proportional to level of significance (i.e., extent of colocalization). “+ molecule name” next to arrow represents condition molecule, and indicates that target colocalization with reference is significantly enhanced for subset of CD36 colocalized with condition. An exception is CD36 colocalization with β_1_-integrin in the periphery region (indicated by * in G), which is significantly enhanced for the subset of CD36 colocalized with CD9 and also for the subset of β_1_-integrin colocalized with CD9. Font size of condition name reflects level of significance (i.e., extent) of enhancement. Dashed arrows indicate that target colocalization with reference is only significant for the subset of CD36 colocalized with the condition. Molecules with no connecting arrow do not exhibit significant colocalization. **(H–N)** Same as A–G but for CD36^G12V^. See Table S1 for the significance P values from which these graphs were generated. The images shown in B, C, D, and I are, respectively, the same as the images shown in Fig. S1, C, D, E, and H. See Table 4 for the number of cells, the number of experimental repeats, and the number of objects per channel per ROI used for analysis.

To study the interactions of CD36 with integrins and tetraspanins in their cellular context, we took an imaging and quantitative colocalization approach. As the interactions between CD36, β_1_-integrin, and the tetraspanins CD9, CD81, and CD151 have been demonstrated previously via biochemical methods such as co-IP (Heit et al., 2013; Huang et al., 2011; Kazerounian et al., 2011; Miao et al., 2001; Primo et al., 2005; Thorne et al., 2000), quantitative imaging yielded complementary insight by investigating these interactions in their native spatial context. We transiently expressed Halo-CD36 in telomerase-immortalized microvascular endothelial (TIME) cells, which express little endogenous CD36 (Dasgupta et al., 2023). The expression level of CD36 in transfected TIME cells was comparable with that in primary MVECs (Fig. S1 A). We then labeled Halo-CD36 with JF549-Halo-ligand and imaged CD36, together with combinations of immunolabeled β_1_-integrin (with the neutral antibody K20 [Byron et al., 2009]) and the tetraspanins CD9, CD81, or CD151, using total internal reflection fluorescence microscopy (TIRFM) (Fig. 1, A–E). The imaged molecules were then detected with sub-pixel localization (Aguet et al., 2013; Jaqaman et al., 2008) and, after correcting for registration shift between the different channels, their colocalization relationships were assessed using conditional colocalization analysis (Vega-Lugo et al., 2022).

Conditional colocalization analysis quantifies the colocalization relationships between three molecular entities. It yields the extent of colocalization between pairs of molecules, as well as whether the colocalization between a pair of molecules is enhanced by either molecule’s colocalization with a third molecule (Vega-Lugo et al., 2022). The approach is object-based, allowing the use of statistical methods based on the nullification of the spatial relationships between objects to assess the significance of any detected colocalization relationship. In this method, colocalization strength is reflected by how much the observed colocalized fraction exceeds that expected by chance, compensating for differences in expression, labelling efficiency, or spatial distribution. Statistical measures—P values and Cohen’s *d* (mean effect size measure) (Maher et al., 2013; Morgan, 2025) —quantify the colocalization strength and enable comparisons across molecules, subcellular regions, and conditions.

We performed conditional colocalization analysis separately for two PM regions, the periphery (a band of ∼4 μm right behind the cell edge) and the center (PM areas > ∼4 μm away from the cell edge) (Dasgupta et al., 2023). In our experiments, the cell edge was a free edge without contact with neighbors (while the cells had neighbors on other sides). Thus, the periphery contained the lamellipodium right behind the free cell edge and part of the lamella (Delorme et al., 2007), with higher CA density (Fig. S1 B) (Ehringer et al., 1999; Mendoza et al., 2015; Schaphorst et al., 1997; Verkhovsky et al., 2003) and different CA dynamics (Dasgupta et al., 2023; Ponti et al., 2004). These peripheral regions may resemble the leading edge of spreading or migrating cells (e.g., tip endothelial cells in the context of angiogenesis) in terms of actin cytoskeleton organization and molecular composition (Gerhardt et al., 2003; Yadunandanan Nair et al., 2022). As our molecules of interest link to CA (directly or indirectly), they could behave differently in the two subregions, as shown previously for CD36 (Dasgupta et al., 2023). In fact, many of the imaged molecules showed higher density in the periphery than in the center (Fig. S1, C–H).

**Figure S2.**
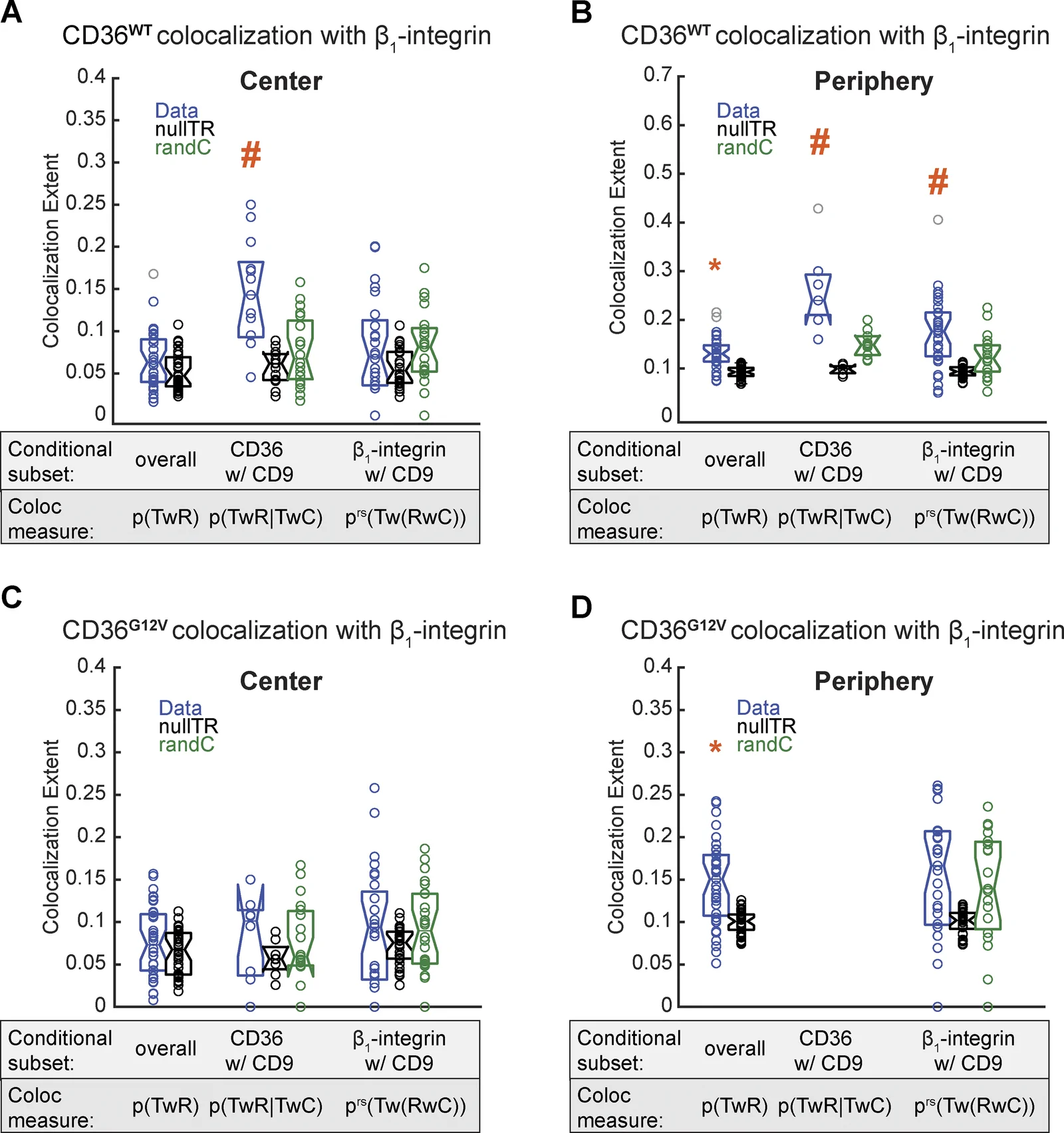
**Analysis of CD36 colocalization with β**_**1**_ **-integrin, conditional on CD9. (A–D)** Details of the colocalization analysis of the indicated three molecules underlying the P values presented in Table S1, for target = CD36^WT^ (A and B) or CD36^G12V^ (C and D), reference = β_1_-integrin, and condition = CD9, in the center region (A and C) or the periphery region (B and D). The measure p(TwR) quantifies the extent of CD36 colocalization with β_1_-integrin regardless of either’s proximity or not to CD9 (“overall”). The measure p(TwR|TwC) quantifies the extent of CD36 colocalization with β_1_-integrin for the subset of CD36 colocalized with CD9 (“CD36 w/ CD9”). The measure p^rs^(Tw(RwC)) quantifies the extent of CD36 colocalization with β_1_-integrin for the subset of β_1_-integrin colocalized with CD9 (“β_1_-integrin w/ CD9”). To assess the significance of colocalization for any of these sets/subsets, the observed colocalization measure (blue) is compared with that obtained when the target molecules (CD36) are replaced with points on a grid (“nullTR” [black]). To assess the significance of the effect of the condition, the observed p(TwR|TwC) and p^rs^(Tw(RwC)) (blue) are compared with their counterparts when the condition molecule locations are randomized (“randC” [green]). To complete their significance testing, p(TwR|TwC) and p^rs^(Tw(RwC)) are also compared with their corresponding p(TwR). Pairwise colocalization is taken as significant (indicated by *) when p(TwR) is significantly greater than its coincidental counterpart (nullTR) (P value ≤ 0.05). The effect of the condition molecule on colocalization is taken as significant (indicated by #) when p(TwR|TwC) or p^rs^(Tw(RwC)) is significantly greater than its coincidental counterparts (both nullTR and randC) and its corresponding p(TwR) (combined type I error = 0.05 for the three tests). See Vega-Lugo et al. (2022) for more analysis details. All measures are shown as boxplots. For each box, individual circles indicate individual cell measurements, the central mark is the median, and the edges are the 25th and 75th percentiles. The notch around the median indicates the 95% confidence interval of the median. Circles with the same color as the box are inliers, while gray circles are outliers. See Table 4 for the number of cells, the number of experimental repeats, and the number of objects per channel per ROI used for analysis.

As expected from their known physical interactions (Berditchevski, 2001; Charrin et al., 2009; Heit et al., 2013; Huang et al., 2011; Kazerounian et al., 2011; Miao et al., 2001; Primo et al., 2005; Thorne et al., 2000; Yauch et al., 2000; Zhang et al., 2009), we found significant colocalization between CD36, β_1_-integrin, and the three tetraspanins (Fig. 1, F, and G; Fig. S2; and Table S1). The colocalization relationships were stronger in the periphery than in the center (Fig. 1, F and G; Fig. S2; and Table S1). The analysis also provided evidence for (statistical) interdependence in the colocalization of CD36 with β_1_-integrin and tetraspanins. Namely, in the periphery, CD36-tetraspanin colocalization was enhanced for the subset of CD36 colocalized with β_1_-integrin, and CD36–β_1_-integrin colocalization was enhanced for the subset of CD36 colocalized with CD9 (Fig. 1 G). In the center, the colocalization of CD36 with β_1_-integrin, β_1_-integrin with CD36, and CD9 with CD36 was significant only for the subset of CD36 colocalized with a third interaction partner. The colocalization between β_1_-integrin and the tetraspanins themselves, on the other hand, was indifferent to CD36 in both PM regions (Fig. 1, F and G).

These results suggest that CD36 is recruited into multimolecular assemblies containing β_1_-integrin and various tetraspanins, which form independently of CD36 (as expected from their known physical interactions [Berditchevski, 2001; Charrin et al., 2009; Yauch et al., 2000; Zhang et al., 2009]). We refer to these groups of colocalizing molecules as “multimolecular assemblies,” because their exact nature is yet to be determined. This is partly due to the spatial scale of our colocalization analysis (100–200 nm), which is within that of higher-order tetraspanin-enriched PM domains (Schmidt et al., 2024; Zuidscherwoude et al., 2015) but is large from a molecular perspective. It is also partly due to the involvement of multiple factors not part of our study (such as lipids) in the formation of these assemblies, among other reasons. Thus, these multimolecular assemblies could be complexes, clusters, or various PM domains (Heit et al., 2013; Kazerounian et al., 2011; Truong Quang and Lenne, 2014; van Deventer et al., 2021). Importantly, CD36 recruitment into these multimolecular assemblies is stronger in the periphery than in the center.

### A mutation in the N-terminal transmembrane domain of CD36 (G12V) reduces its interactions with β_1_-integrin and CD9

**Figure S3.**
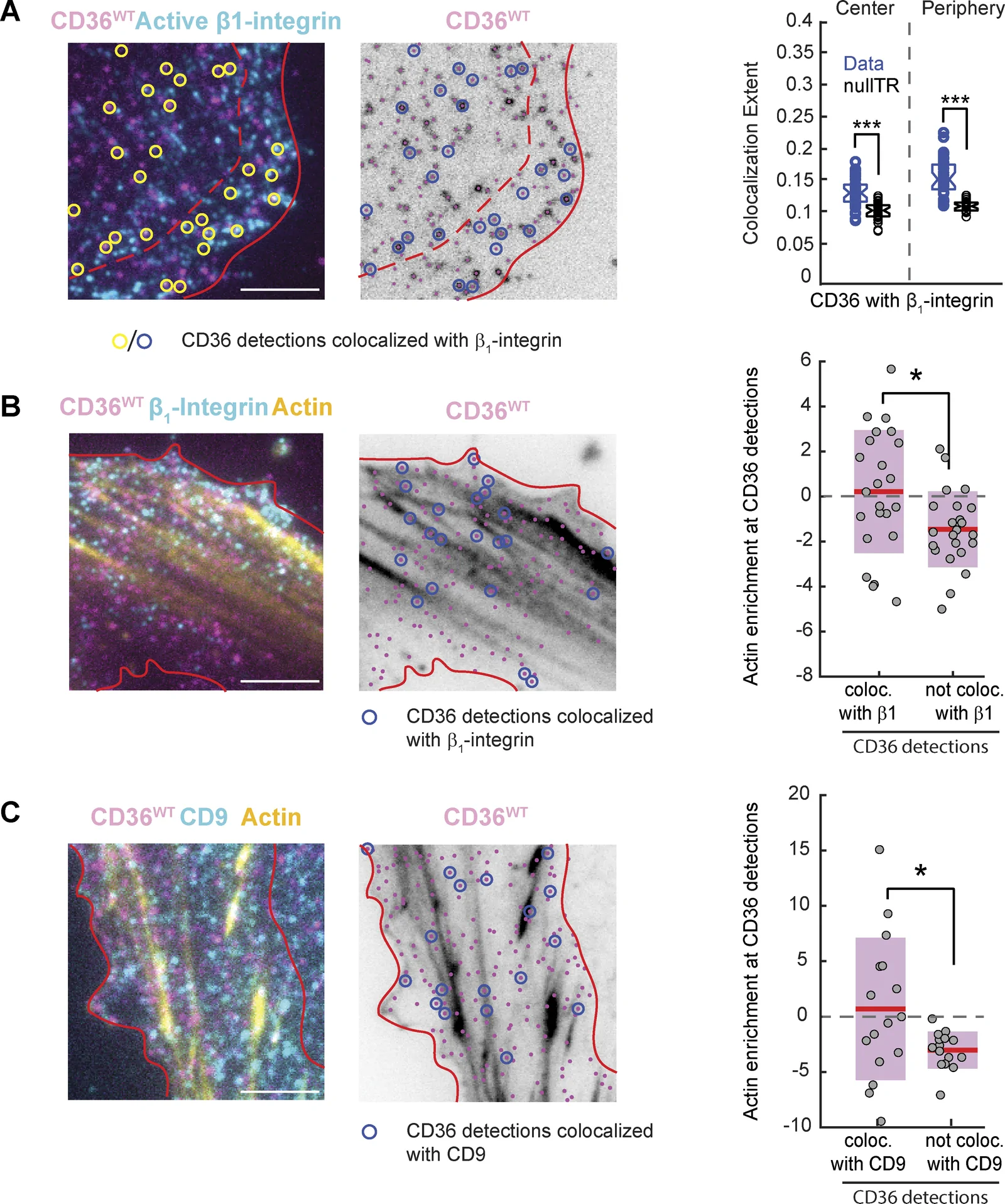
**CD36 colocalizes with activated β**_**1**_ **-integrin; CD36 colocalized with β**_**1**_ **-integrin or CD9 has higher actin enrichment around it than CD36 not colocalized with these interaction partners. (A)** Representative two-color fixed cell image of CD36^WT^ (magenta) and active β_1_-integrin (cyan; detected via the activation-specific antibody 12G10) in the left panel, and CD36 particle detections (shown as dots) overlaid on the CD36 channel in the middle panel. CD36^WT^ colocalized with active β_1_-integrin is indicated by yellow/blue circles in the left/middle panel. Solid red lines show the segmented cell edge; dashed lines specify the boundary between center and periphery regions. Quantification of CD36^WT^ colocalization with active β_1_-integrin, measured separately in cell center and periphery regions, is shown in the right panel. Right panel plot details are as in Fig. S2. Scale bar, 5 μm. *N* = 32 cells from four experimental repeats. **(B and C)** Left panels: Representative three-colored fixed cell images of CD36^WT^ (magenta), β_1_-integrin (B) or CD9 (C) (both in cyan), and actin (yellow). Middle panels: CD36 particle detections (shown as dots) overlaid on the actin channel (same as that shown in left panels). Right panels: Quantification of actin enrichment measured at CD36^WT^ detections colocalized with or not colocalized with β_1_-integrin (B) or CD9 (C). Solid red lines in image panels indicate ROI segmented based on actin intensity. In enrichment analysis plots (right panels), circles, red lines, shaded bars, and asterisks are as in Fig. 2. Scale bar, 5 μm. *N* = 32 cells from four experimental repeats.

CD36’s interactions with β_1_-integrin (including the activated form [Fig. S3 A]) and various tetraspanins may allow CD36 to link to CA, as these molecules have been shown to interact with actin (Bailey et al., 2011; Coffey et al., 2009; Geiger et al., 2001; Humphries et al., 2006; Kim et al., 2011; Sala-Valdés et al., 2006; Schmidt et al., 2024; Shigeta et al., 2003; Takeda et al., 2007; Zhang et al., 2009). To gather preliminary evidence, we co-imaged CD36, CA (phalloidin-stained), and either β_1_-integrin or CD9, and assessed whether CD36 colocalized with these partners was in areas with higher CA intensity than CD36 not colocalized with them. Indeed, CD36 colocalized with β_1_-integrin or CD9 had higher local CA intensity than CD36 not colocalized with either molecule (Fig. S3, B and C), consistent with β_1_-integrin and CD9 providing a link between CD36 and CA.

**Figure S4.**
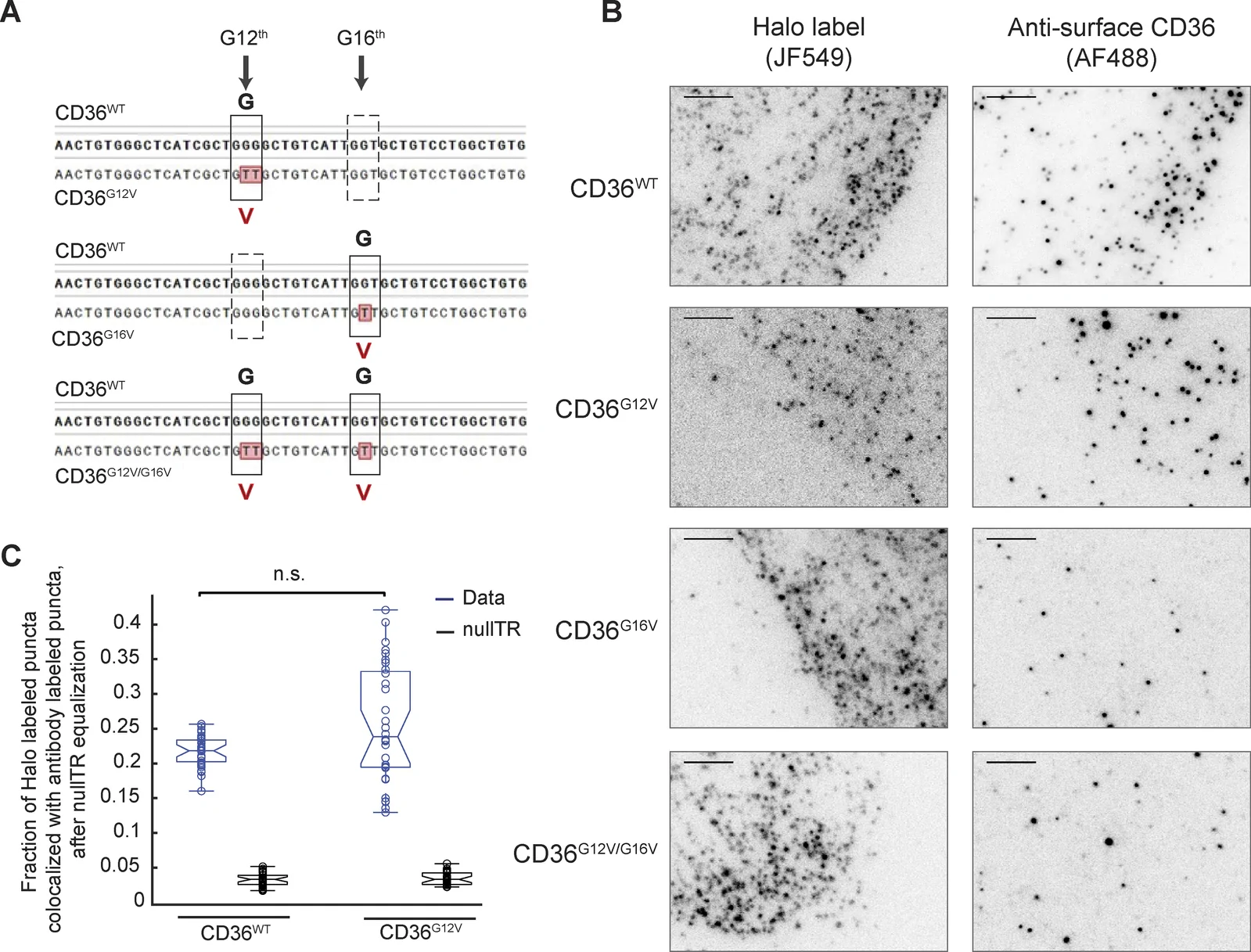
**Sequence confirmation and surface expression of different CD36 mutants. (A)** Sequence of WT (bold) compared with CD36^G12V^, CD36^G16V^, and CD36^G12V/G16V^. The change in codon from glycine to valine is shown in a black solid box with the nucleotides highlighted in red color. **(B)** Two-color TIRFM images of HaloTag-fused WT (CD36^WT^) and mutated CD36 (CD36^G12V^, CD36^G16V^, and CD36^G12V/G16V^) expressed in TIME cells, labeled using JF549-conjugated Halo ligand to assess total expression (first column) and an anti-CD36 antibody that targets the CD36 ectodomain to assess surface expression (second column). Scale bar, 5 μm. **(C)** To assess the fraction of CD36^WT^ and CD36^G12V^ expressed on the cell surface, the fraction of Halo-labeled puncta (labeling CD36 regardless of its surface expression) colocalized with antibody-labeled puncta (labeling only CD36 on the cell surface) was calculated. The plot shows these fractions after compensating for the slightly different overall expression levels of WT and mutant CD36, achieved by nullTR equalization. NullTR equalization = multiplying the data and nullTR values of one condition (CD36^WT^ in this case) with a factor to make its nullTR have the same median as the nullTR of the condition it is compared with (CD36^G12V^ in this case). The idea is that this compensates for different overall expression levels, as nullTR reflects the number of objects used in colocalization analysis (Vega-Lugo et al., 2022), thus allowing the direct comparison of the colocalization fractions between the two conditions. Plot details are as in Fig. S2. *N* = 32 cells for each of CD36^WT^ and CD36^G12V^, from four experimental repeats.

The above results are consistent with the hypothesis that CD36 interactions with β_1_-integrin and tetraspanins contribute to the link between CD36 and CA. To fully test this hypothesis, we sought to inhibit these interactions. Given integrins’ broad role in actin network dynamics (Bailey et al., 2011; Coffey et al., 2009; Sala-Valdés et al., 2006; Takeda et al., 2007; Toribio and Yáñez-Mó, 2022; Zhang et al., 2009) and the redundancy between tetraspanins (Charrin et al., 2009), we mutated CD36 to disrupt its association with its interaction partners. Recently, the GXXXG sequence motif in the N-terminal transmembrane helix of CD36 (residues 12–16) was shown to mediate CD36–CD9 interactions (Huang et al., 2023). Thus, we generated three CD36 mutants: a double mutant CD36^G12V/G16V^ and two single mutants CD36^G12V^ and CD36^G16V^. Out of the three, only CD36^G12V^ was expressed on the cell surface similarly to WT CD36 (CD36^WT^) (Fig. S4; and Fig. S1, C and H).

**Figure S5.**
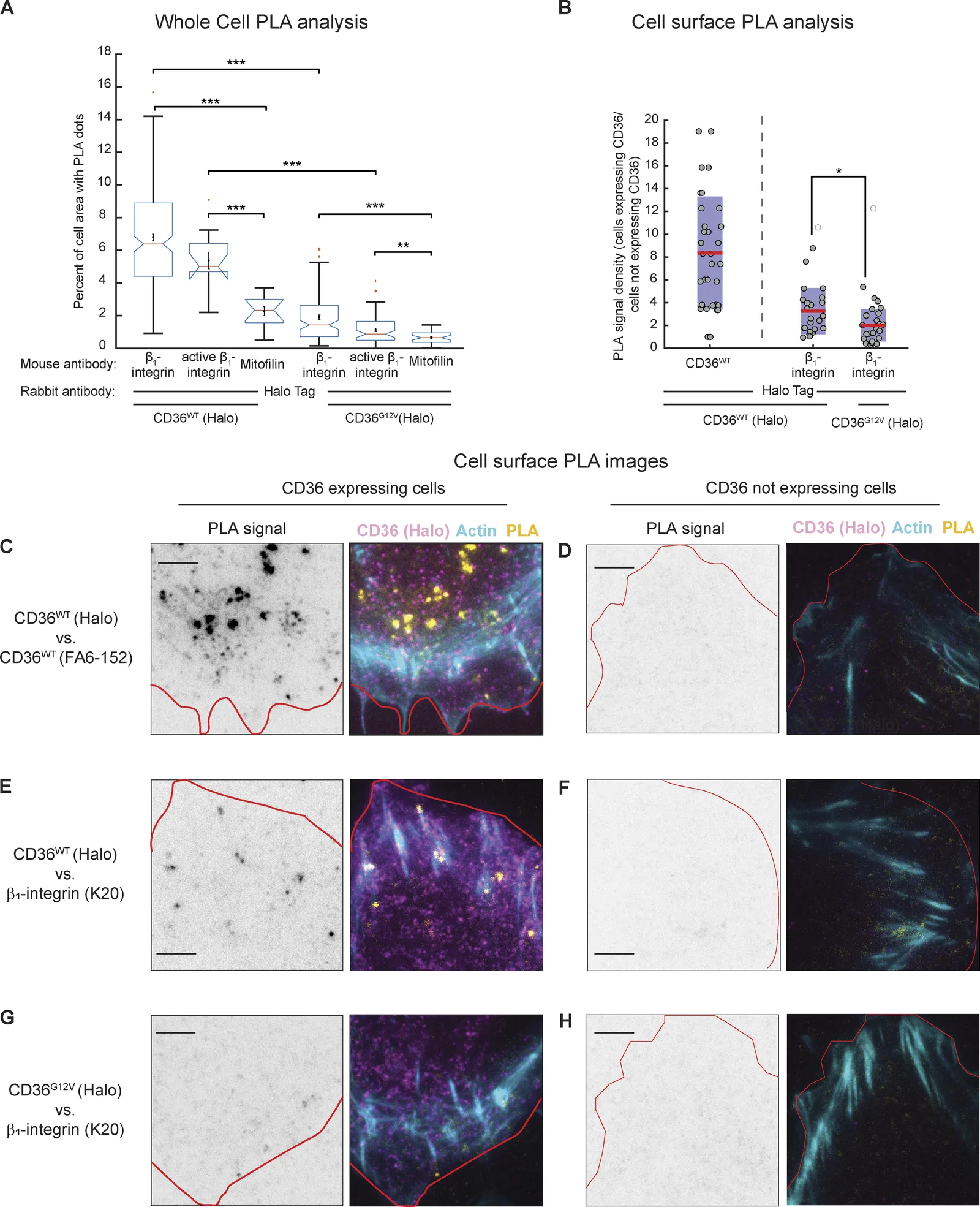
**PLA indicates that CD36 forms proximity associations with β**_**1**_ **-integrin that are reduced by the G12V mutation. (A)** Quantification of PLA signal from spinning disk confocal imaging, representing the extent of interaction between the antibody pairs against the proteins indicated below the x-axis throughout the whole cell. The boxplot for each pair displays the percentage of the cell area occupied by the PLA puncta for that pair. Nonparametric ANOVA Kruskal–Wallis test followed by a two-sided Wilcoxon rank-sum test were employed to compare the different conditions, with ** and *** indicating P ≤ 0.01 and 0.001, respectively. *N* = 233 (CD36^WT^ vs. K20), 120 (CD36^G12V^ vs. K20), 12 (CD36^WT^ vs. 12G10), 71 (CD36^G12V^ vs. 12G10), 12 (CD36^WT^ vs. mitofilin), and 43 (CD36^G12V^ vs. mitofilin) from two to three experimental repeats per pair. **(B)** Quantification of PLA signal from TIRFM, representing the extent of interaction between the antibody pairs against the proteins indicated below the x-axis on the cell surface. The extent of interaction is expressed as the ratio of PLA signal detection density in CD36-expressing cells to PLA signal detection density in cells not expressing CD36. Circles, red lines, shaded bars, and * are as in Fig. 2. *N* = 24–25 cells for each pair, combined from 3 experimental repeats. **(C–H)** Representative fixed cell images showing PLA signals acquired via TIRFM (corresponding to the quantifications shown in B) for the indicated protein pairs in transfected cells expressing CD36 (C, E, and G) or not expressing CD36 (D, F, and H). Left panels show PLA signal alone, while right panels show merged images of CD36 (magenta), actin (phalloidin, cyan), and PLA puncta (yellow). Solid red line indicates segmented cell edge. Scale bar (in all images), 5 μm.

Consistent with this motif mediating CD36–CD9 interactions (Huang et al., 2023), three-color imaging and conditional colocalization analysis revealed that the G12V mutation reduced CD36–CD9 colocalization, in both center and periphery (Fig. 1, H–N and Table S1). Interestingly, the mutation also reduced CD36–β_1_-integrin colocalization, even down to insignificance in the center (Fig. 1, H–N; Fig. S2, C and D; and Table S1). Note that integrins also contain a GXXXG motif, which mediates many PM protein–protein interactions (Schneider and Engelman, 2004; Teese and Langosch, 2015). To provide further evidence that the reduction in CD36–β_1_-integrin colocalization reflected disruption of their interactions, we employed proximity ligation assay (PLA) (Alam, 2018; Saini et al., 2025, *Preprint*), probing associations at a higher resolution (∼40 vs. ∼200 nm). We detected significant PLA signals for CD36^WT^–β_1_-integrin (total and activated), significantly above the mitofilin-negative control (Fig. S5 A). Upon the G12V mutation, these interactions were significantly reduced, both overall and at the cell surface (Fig. S5, A–H), providing evidence that the GXXXG motif contributes to CD36–β_1_-integrin as well as CD36–CD9 interactions (Huang et al., 2023). The effect of the mutation on CD36–CD81 and CD36–CD151 colocalization varied by subregion: CD36–CD81 colocalization increased in the center and decreased in the periphery, while CD36–CD151 colocalization showed the opposite trend (Fig. 1, M and N; and Table S1). Finally, the mutation also shifted the colocalization interdependence landscape, abolishing most of the colocalization interdependence for CD36–CD9–β_1_-integrin and CD36–CD151–β_1_-integrin, while enhancing it for CD36–CD81–β_1_-integrin (both center and periphery).

These results provide evidence that the G12V mutation weakens CD36 interactions with CD9 and β_1_-integrin across the PM. It also alters CD36 interactions with CD151 and CD81, weakening them in some regions (periphery/center for CD81/CD151) while potentially strengthening them in others (center/periphery for CD81/CD151).

### G12V mutation reduces coupling between CD36 mobility type and CA dynamics

The G12V mutation provided us with a molecular handle to test the hypothesis that CD36 interactions with β_1_-integrin and tetraspanins contribute to the link between CD36 and CA. Previous studies have shown that CD36 mobility at the PM is influenced by CA (Dasgupta et al., 2023; Jaqaman et al., 2011). Therefore, if CD36–β_1_-integrin–tetraspanin interactions contribute to the link between CD36 and CA, we expect the mobility of CD36^G12V^ to be less coupled to CA architecture and dynamics than that of CD36^WT^.

To this end, we employed our previously developed live-cell single-molecule imaging–fluorescent speckle microscopy (SMI-FSM) approach (Dasgupta et al., 2023). We expressed Halo-CD36^WT^ or Halo-CD36^G12V^ in TIME cells stably expressing low levels of mNeonGreen-actin (TIME-mNGrActin cell line), such that CA appeared as a collection of fluorescent speckles when imaged via TIRFM (Danuser and Waterman-Storer, 2006). We performed live-cell SMI of JF549-labeled CD36 at 10 Hz simultaneously with FSM of CA at 0.2 Hz (Video 1, 2, 3, and 4), probing each at its relevant timescale (Dasgupta et al., 2023). CD36 SMs were tracked and segmented into tracklets of a single mobility type (Jaqaman et al., 2008; Vega et al., 2018). CA speckles were tracked and cleaned of artifactual events (Dasgupta et al., 2023; Ponti et al., 2003). CD36 tracklets were then matched spatially and temporally to neighboring CA speckles, yielding CA properties per CD36 tracklet (Dasgupta et al., 2023).

**Video 1.** **SMI of HaloTag-labeled CD36** ^**WT**^ **(left) and CD36** ^**G12V**^ **(right) labeled with JF549 in TIME-mNGrActin cells.** The video consists of 49 SMI frames (acquired at 10 Hz) within one FSM interval. It excludes the first and last SMI frames of the interval (which coincide with the FSM frames). Each displayed area is 16.2 × 16.2 μm^2^, cropped from the full acquired image (27.62 × 64.8 μm^2^) for visual clarity.

**Video 2.** **FSM of CA visualized as mNeonGreen-Actin speckles in the same TIME-mNGrActin cells as in Video S1 expressing CD36** ^**WT**^ **(left) or CD36** ^**G12V**^ **(right).** Video consists of 11 FSM frames (acquired at 0.2 Hz), i.e., 10 FSM intervals. Each displayed area is 16.2 × 16.2 μm^2^, cropped from the full acquired image (27.62 × 64.8 μm^2^) for visual clarity.

**Video 3.** **SM particle tracking.** Zoomed-in areas of Video S1 showing particle tracks overlaid on the fluorescence images for CD36^WT^ (left) and CD36^G12V^ (right). Random track colors are used to help distinguish between neighboring tracks. Each displayed area is 8.1 × 8.1 μm^2^ for visual clarity.

**Video 4.** **FSM speckle detection and tracking.** Zoomed-in areas of Video S2 showing speckle detections and tracks overlaid on the FSM images, for CD36^WT^ (left) and CD36^G12V^ (right). Circles and triangles represent primary and secondary speckle detections (Mendoza, Michelle C et al. “QFSM to measure actin dynamics.” Current protocols in cytometry vol. Chapter 2 (2012)). Tracks are shown as red lines. Each displayed area is 8.1 × 8.1 μm^2^ for visual clarity.

For the mobility analysis of CD36 tracks, we employed divide-and-conquer moment scaling spectrum (DC-MSS) transient diffusion analysis, where the mobility type of a tracklet was reflected by its MSS slope (Dasgupta et al., 2023; Ferrari et al., 2001; Vega et al., 2018). MSS slopes ≈0.5, ≈0, <0.5, and >0.5 indicated, respectively, free (Brownian) diffusion, immobility, sub-diffusion (e.g., confined diffusion), and super-diffusion. To assess the extent of coupling between CD36 mobility type and CA architecture and dynamics, we performed multivariate regression (MVRG) analysis of tracklet MSS slope on the local CA speckle properties at each tracklet (Dasgupta et al., 2023). MVRG analysis describes the (mathematical) dependence of a tracklet property (such as MSS slope) on the local CA speckle properties of interest. We did the analysis on a per-cell basis, utilizing four subsets of SM tracklets, grouped by their PM location (center or periphery) combined with their local CA speckle density (low or high, i.e., below or above the average speckle density = 1.3 speckles/µm^2^) (Dasgupta et al., 2023). Subdivision into low and high speckle density was necessary to satisfy the linearity assumption of MVRG, as the relationship between certain SM properties and local speckle density exhibited an inverted V-shape, most likely due to the discrete nature of speckles and spatial sampling by SM tracklets (Dasgupta et al., 2023).

**Figure S6.**
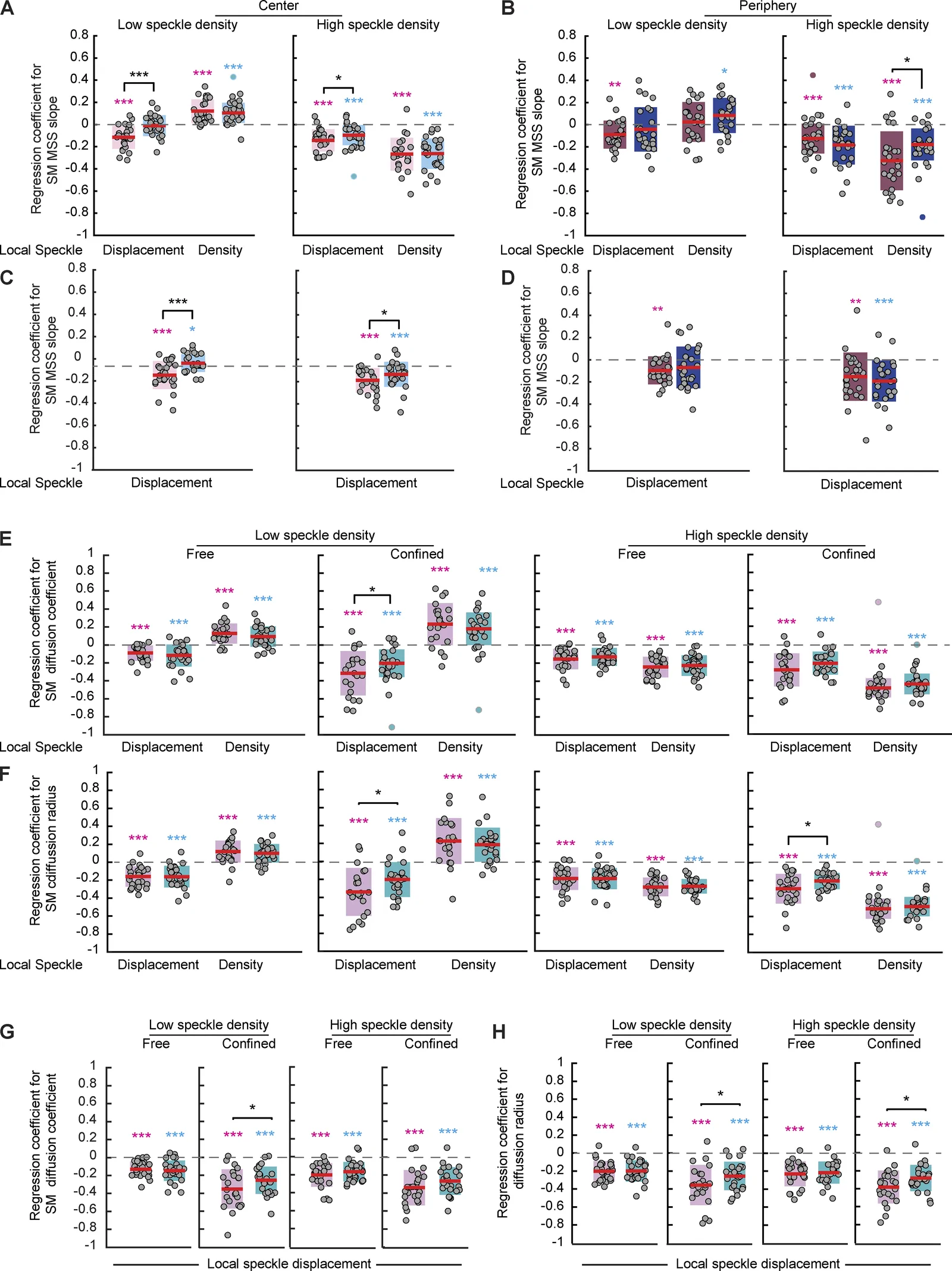
**Regression coefficients from MVRG analysis on local CA speckle displacement and density, and from MVRG analysis on local CA speckle displacement alone. (A and B)** Regression coefficients of CD36^WT^ and CD36^G12V^ MSS slope in the center (A) and periphery (B) regions from MVRG analysis on speckle displacement and density. This is the same MVRG analysis as in Fig. 2, A and B, but showing both regression coefficient components here (while Fig. 2, A and B show only the displacement component). **(C and D)** Regression coefficients of CD36^WT^ and CD36^G12V^ MSS slope in the center (C) and periphery (D) regions from MVRG analysis on speckle displacement alone. **(E and F)** Regression coefficients of CD36^WT^ and CD36^G12V^ diffusion coefficient (E) and diffusion radius (F) on speckle displacement and density. This is the same MVRG analysis as in Fig. 4, A and B, but showing both regression coefficient components here (while Fig. 4, A and B show only the displacement component). **(G and H)** Regression coefficients of CD36^WT^ and CD36^G12V^ diffusion coefficient (G) and diffusion radius (H) from MVRG analysis on speckle displacement alone. All figure details (including significance asterisks) are as in Fig. 2 and Fig. 4. Comparing A vs. C, and B vs. D, and E vs. G and F vs. H shows very similar regression coefficients on displacement whether MVRG is performed on displacement and density or displacement alone. At the same time, including the density in the MVRG analysis yields somewhat cleaner results for the displacement regression coefficients (e.g., the dense-periphery analysis in B vs. D). See Table 5 for the number of cells, the number of experimental repeats, and the number of SM tracklets and speckles per ROI used for analysis.

We performed MVRG analysis of the MSS slope on various CA speckle properties: displacement (reflecting CA network fluctuations), density and intensity (reflecting CA density), and lifetime (reflecting CA network stability) (Dasgupta et al., 2023). Only displacement and density yielded significant MVRG coefficients, as in our previous study (Dasgupta et al., 2023). The MVRG coefficients on density showed the previously observed inverted V-shape (Fig. S6) (Dasgupta et al., 2023). Therefore, we focused our analysis here on the CA speckle displacement component.

**Figure 2.**
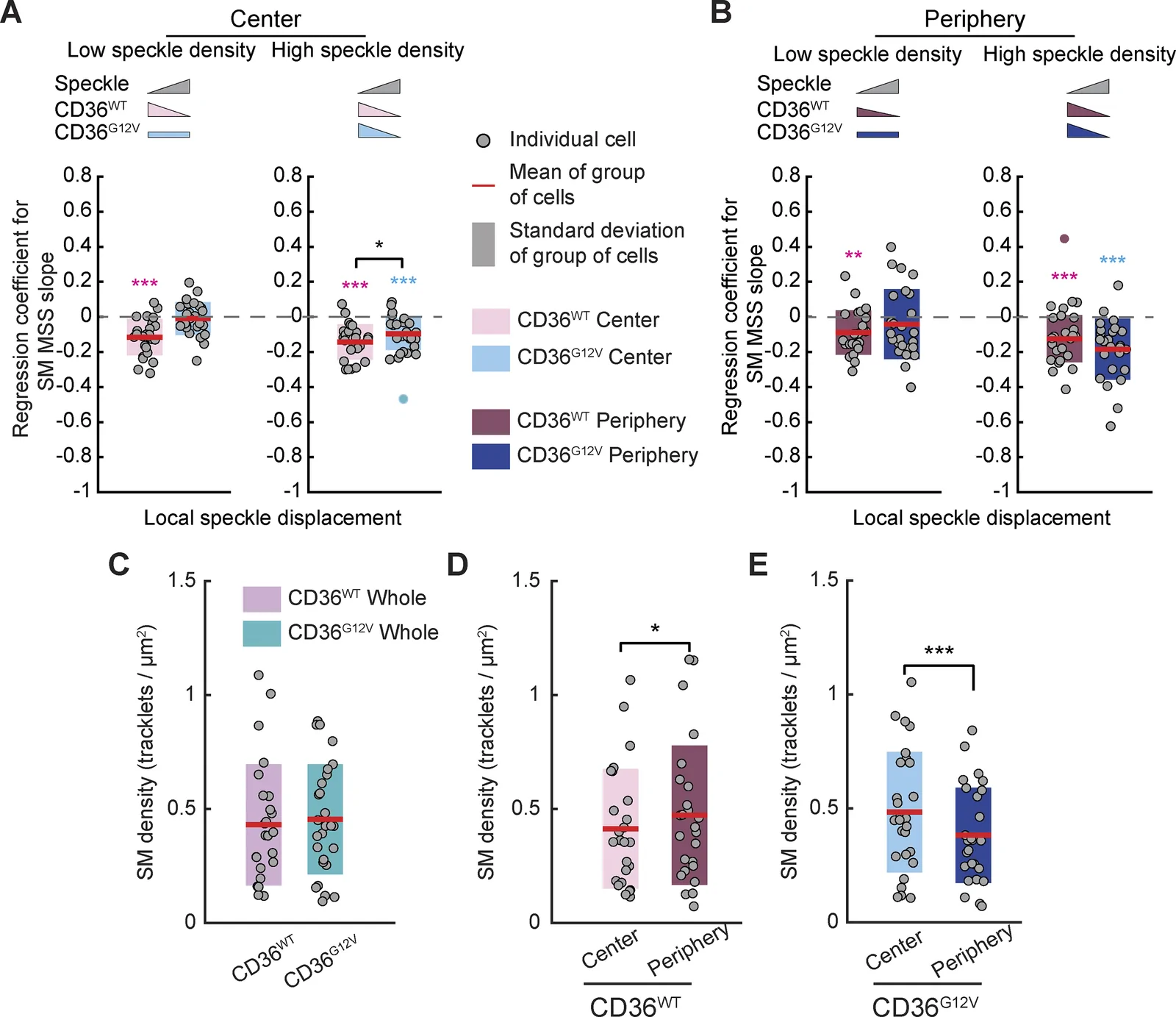
**G12V mutation reduces coupling between CD36 mobility type and local CA speckle displacement and redistributes CD36 from periphery to center. (A and B)** Regression coefficients of CD36^WT^ and CD36^G12V^ MSS slope on local CA speckle displacement, from MVRG analysis on speckle displacement and density (see Fig. S6 for corresponding regression coefficients on density). MVRG analysis was performed separately for the four indicated subgroups of SM tracklets based on subcellular region (center [A] and periphery [B]) combined with local CA speckle density. Circles indicate individual cell measurements; gray circles indicate inliers while non-gray circles indicate outliers. Red lines and shaded bars show mean and standard deviation, respectively, over a group of cells representing WT or mutant. MVRG coefficients significantly different from zero (dashed gray line) are indicated by colored asterisks above the MVRG coefficient plots. Significant differences between WT and mutant are indicated by black asterisks above the bracket. For both, *, **, and *** indicate P values ≤ 0.05, 0.01, and 0.001, respectively. In all tests, P values > 0.05 are not explicitly indicated to reduce clutter. Inset pictograms: visual summary of MVRG results. Triangle slope and direction represent significance and sign (positive or negative) of MVRG coefficient; rectangles indicate nonsignificant MVRG coefficients. **(C)** SM tracklet density for WT and mutant CD36 in the whole imaged region. **(D and E)** SM tracklet density in center and periphery regions for WT (D) and mutant (E) CD36. In C–E, circles, red lines, shaded bars, and black asterisks (or lack thereof) are as in A and B. See Table 5 for number of cells, number of experimental repeats, and number of SM tracklets and speckles per ROI used for analysis.

CD36^WT^ MSS slope showed a significant negative MVRG coefficient on CA speckle displacement for all subgroups (Fig. 2, A and B), indicating negative coupling between CA speckle displacement and PM protein mobility, similar to our earlier findings (Dasgupta et al., 2023). Negative coupling means that CD36^WT^ molecules in areas with more CA fluctuations tend to exhibit more constrained mobility, and vice versa. For CD36^G12V^, the MVRG coefficient was not significant for SMs at low local CA speckle density in both the periphery and center, and its magnitude was reduced at high speckle density in the center (Fig. 2, A and B). The larger change at low speckle density is consistent with our previous finding that differences in CA coupling manifest more readily at low local CA speckle density (Dasgupta et al., 2023).

Of note, the CA speckle signals observed here represent a composite of actin cytoskeletal elements, which could influence CD36 mobility differently (and some may not influence it at all). Despite this heterogeneity, our results show clear relationships between CA dynamics and CD36 mobility, indicating that the molecular link between CD36 and CA is sufficiently strong to be observed even when averaged over a variety of CA architectures and components. Importantly, our results indicate that the G12V mutation weakens the coupling between CD36 mobility type and CA, providing evidence that CD36 interactions with β_1_-integrin and tetraspanins contribute to the molecular link between CD36 and CA.

### CD36^G12V^ localizes less than CD36^WT^ in the actin-rich periphery region of the PM

Another evidence for the reduced coupling between CD36 and CA due to the G12V mutation came from the distribution of CD36 molecules across the PM. In both fixed and live cells, CD36^WT^ preferentially localized in the periphery region of the PM (Fig. 2, C and D; and Fig. S1 C), where CA density was higher (Fig. S1 B). In contrast, CD36^G12V^ preferentially localized in the center region (Fig. 2, C and E; and Fig. S1 H). Since β_1_-integrin and the investigated tetraspanins also predominantly localize in the periphery (Fig. S1, D–G), their interactions with CD36 likely retain CD36^WT^ there, while the loss/reduction of these interactions leads to a redistribution of CD36^G12V^ toward the center. Importantly, these trends are consistent with the hypothesis that the CD36–CA link is weakened by the G12V point mutation, providing further evidence that CD36 interactions with β_1_-integrin and tetraspanins contribute to this link.

### CD36^G12V^ exhibits a higher fraction of confined diffusion than CD36^WT^, albeit the confinement of CD36^G12V^ is weaker

**Figure 3.**
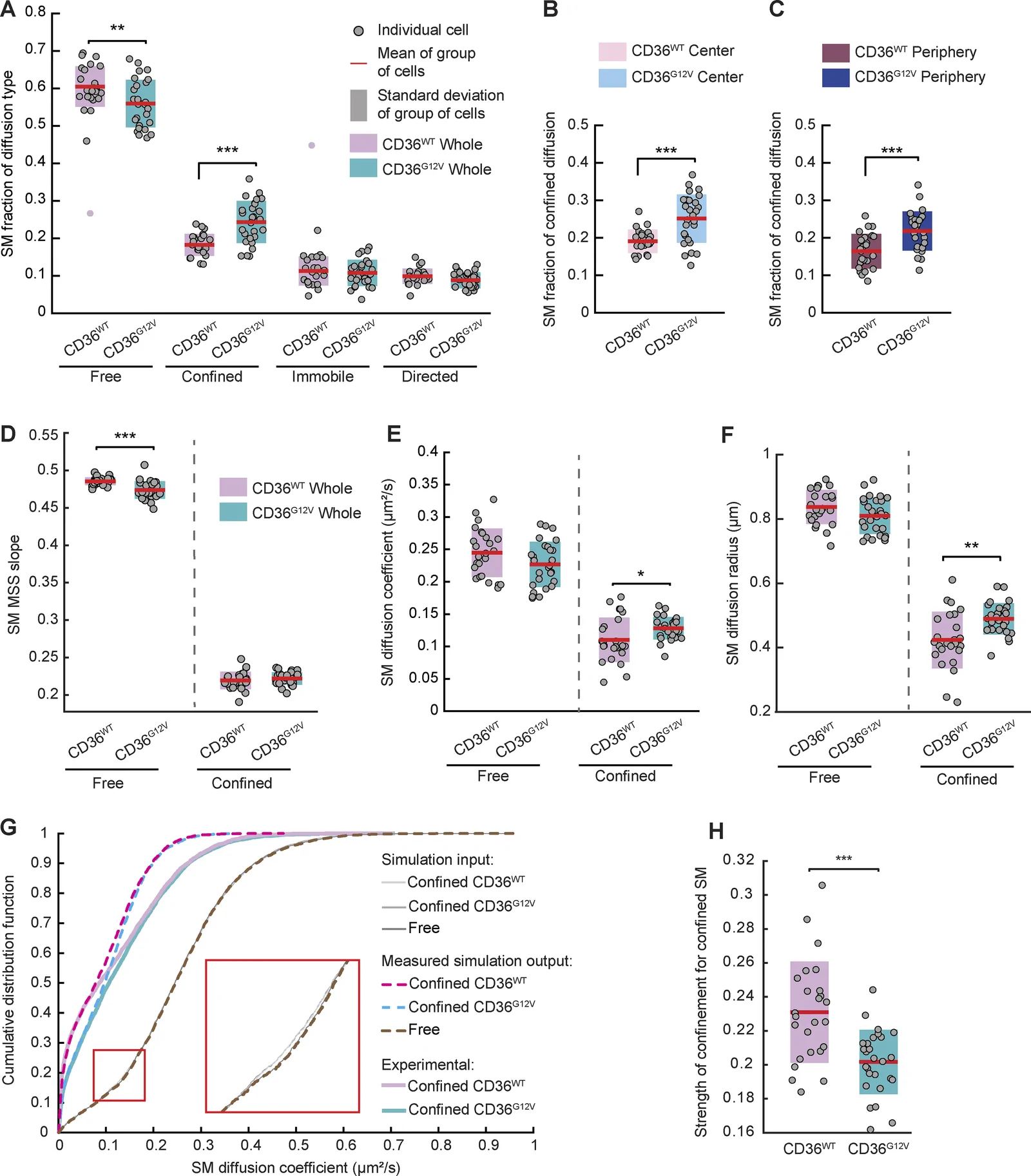
**G12V mutation increases fraction of CD36 molecules undergoing confined diffusion, but with weaker confinement strength. (A)** Fraction of CD36^WT^ and CD36^G12V^ tracklets undergoing the indicated motion types in the whole imaged region. **(B and C)** Fraction of CD36^WT^ and CD36^G12V^ tracklets undergoing confined diffusion in the center (B) and periphery (C) regions. **(D)** MSS slope for free and confined CD36^WT^ and CD36^G12V^ in the whole imaged region. **(E and F)** Diffusion coefficient (E) and diffusion radius (F) for free and confined CD36^WT^ and CD36^G12V^ tracklets in the whole imaged region. **(G)** The cumulative distribution of SM diffusion coefficient as input into simulations (solid gray lines), as measured for simulated free and confined tracks (dashed lines), and as measured experimentally (solid non-gray lines). The red box zooms in on an area for visual aid to distinguish between highly overlapping lines. **(H)** The strength of confinement as derived from the scaling exponent of the mean squared displacement. In all panels but G, circles, red lines, shaded bars, and asterisks (or lack thereof) are as in Fig. 2. See Table 5 for number of cells, number of experimental repeats, and number of SM tracklets and speckles per ROI used for analysis.

What are the consequences of perturbing CD36 interactions with β_1_-integrin and tetraspanins and weakening the CD36–CA link for CD36 mobility? Unexpectedly, we found that the mutant exhibits more confined diffusion than WT. First, a higher fraction of tracklets was classified as confined for CD36^G12V^ than for CD36^WT^ (∼24 vs. ∼18% on average), at the expense of free tracklets (Fig. 3 A). This was in both center and periphery (Fig. 3, B and C). Second, free tracklets themselves had an overall lower MSS slope in the mutant vs. WT (Fig. 3 D), indicating a greater extent of hidden confined diffusion (or immobility) within the free tracklets. This confined diffusion (or immobility) was “hidden” because it happens transiently at a faster timescale than our imaging temporal resolution (0.1 s). Such short-lived instances of confinement (or immobility) would go undetected, but they would reduce the overall MSS slope of the classified tracklets (Ritchie et al., 2005).

Yet the confined CD36^G12V^ tracklets had a larger diffusion coefficient and diffusion radius (reflecting the area within which the molecule’s diffusion is confined) than those of CD36^WT^ (Fig. 3, E and F). Free SM tracklets, on the other hand, showed no difference in their diffusion coefficient or radius between CD36^WT^ and CD36^G12V^ (Fig. 3, E and F). The concomitant increase in the diffusion coefficient and diffusion radius for CD36^G12V^ confined tracklets suggested that these two properties were coupled. This, together with the similar diffusion coefficient of free CD36^WT^ and CD36^G12V^, led us to hypothesize that WT and mutant CD36 have fundamentally the same diffusion coefficient, but that, for the confined subset, different confinement areas between WT and G12V led to different effective diffusion coefficients at our imaging time resolution (0.1 s).

To test this hypothesis, we simulated two sets of confined tracks, both using the diffusion coefficient distribution of free CD36^WT^, combined with the diffusion (i.e., confinement) radius distribution of confined CD36^WT^ or confined CD36^G12V^. As a control, we simulated a set of freely diffusing tracks. In all simulations, track lifetimes ranged from 20 to 40 time points, sampled at 0.1 s, similar to experimental tracklets. We then applied diffusion analysis to the simulated tracks, similar to the experimental tracklets.

Consistent with our hypothesis, the calculated diffusion coefficient of simulated confined tracks was shifted toward higher values for CD36^G12V^ than for CD36^WT^ (Fig. 3 G). For the free diffusion tracks, the calculated diffusion coefficient matched the input (Fig. 3 G). Remarkably, not only did the simulated confined diffusion coefficients follow the expected trend between CD36^WT^ and CD36^G12V^, but the diffusion coefficient values matched those measured experimentally in the lower half of the distribution, which is where the separation between CD36^WT^ and CD36^G12V^ is largest (Fig. 3 G). The discrepancy in the upper half of the distribution (where there is little difference between WT and mutant) probably stems from the simplified nature of the simulations, where simulated confined tracks were confined at all times, while experimental confined tracklets probably contained some hidden, transient free diffusion within them, thus increasing their effective diffusion coefficient.

These results indicate that indeed the differences in confined motion properties between CD36^WT^ and CD36^G12V^ stem from the mutant residing in larger confinement areas than WT. The larger confinement areas of the mutant, in turn, most likely stem from the mutant experiencing a weaker force of confinement than WT, as revealed by estimating the strength of confinement from the scaling exponent of the mean squared displacement (Doliwa and Heuer, 1998) (Fig. 3 H). Overall, these results imply that perturbing CD36 interactions with β_1_-integrin and tetraspanins and weakening the CD36–CA link lead to significant changes in CD36 mobility.

### The diffusion properties of confined CD36^G12V^ molecules are less coupled to CA than those of CD36^WT^

**Figure S7.**
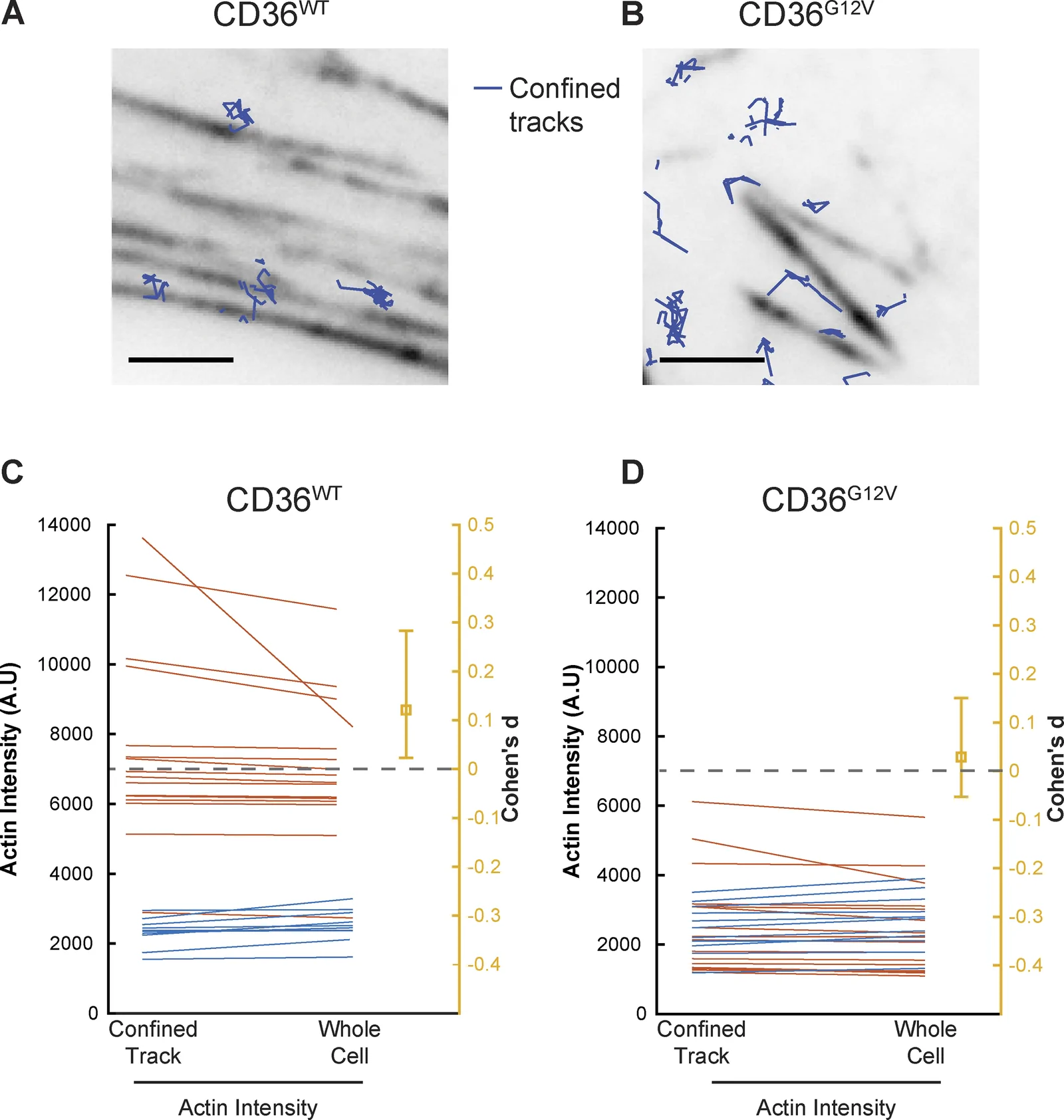
**Confined CD36** ^**WT**^ **tracks tend to be in locations with higher-than-average cortical actin intensity, while confined CD36** ^**G12V**^ **trajectories do not. (A and B)** Representative live cell images of confined single-molecule trajectories of CD36^WT^ (A) or of CD36^G12V^ (B) overlaid on SiR-actin–labeled actin cytoskeleton. Scale bar, 5 μm. For visual clarity, only confined tracks are shown, while all other types (free, immobile, directed, or too short to classify) are not. **(C and D)** Actin intensity (in arbitrary units [A.U.]) measured at confined CD36 tracks or in the whole cell (i.e., average) for CD36^WT^ (C) or CD36^G12V^ (D). Lines connect paired measurements from individual cells, comparing actin intensity measured at confined tracks to whole cell actin intensity. Red/blue lines indicate local enrichment/depletion at confined tracks relative to whole cell levels. Paired, right-tailed Wilcoxon rank-sum test comparing the actin intensity at confined tracks vs. whole cell levels yields P = 0.0873 for CD36^WT^ and P = 0.3706 for CD36^G12V^. The right y-axes show effect size (Cohen’s *d*) of local actin intensity relative to whole cell measurements. The error bar shows the 95% confidence interval obtained from 3,000 bootstrap samples. The 95% confidence interval of CD36^WT^ does not cross zero (dashed gray line), indicating a significant difference between local and whole cell, while the 95% confidence interval of CD36^G12V^ crosses zero, indicating a lack of difference between local and whole cell. *N* = 28 cells from three experimental repeats in C and 28 cells from two experimental repeats in D.

The weaker nature of confinement of CD36^G12V^ compared with CD36^WT^ suggests that different or altered mechanisms underlie mutant confinement compared with WT. Interestingly, SMI of CD36 together with simultaneous imaging of CA in live cells using SiR-actin (providing continuous labelling of actin to assess CA density) showed that confined CD36^WT^ tracklets tended to reside in areas of relatively high CA density compared with the whole cell, while confined CD36^G12V^ did not (Fig. S7). These results further suggest that the mechanisms underlying confined motion in the mutant differ from those in WT, with CA playing a reduced role in the confinement of CD36^G12V^.

To investigate this further, we quantified the coupling between CD36 tracklet diffusion properties and CA dynamics using SMI-FSM and MVRG analysis. In our previous study, we found that confined CD36^WT^ tracklets had stronger coupling (larger MVRG coefficient magnitude) to CA speckle displacement than free CD36^WT^ tracklets (Dasgupta et al., 2023). What about CD36^G12V^? To address this question, we performed MVRG analysis of CD36^WT^ and CD36^G12V^ diffusion properties, namely diffusion coefficient and diffusion radius. We performed the analysis for four subsets of tracklets, as grouped by tracklet diffusion type (free or confined) combined with local CA speckle density (low or high) (Dasgupta et al., 2023).

**Figure 4.**
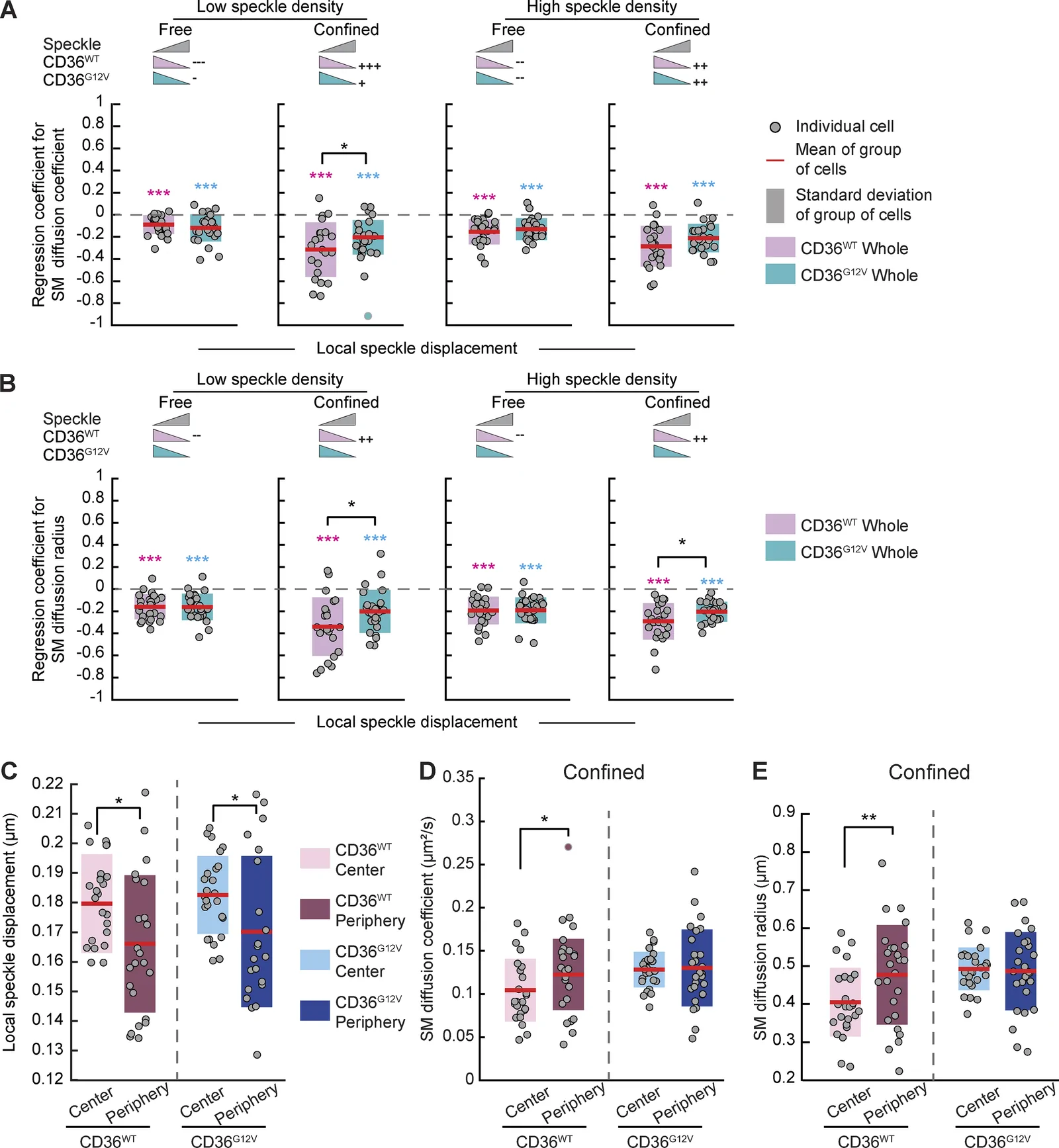
**Confined CD36** ^**G12V**^ **molecules exhibit less coupling to CA speckle displacement than confined CD36** ^**WT**^ **molecules. (A and B)** Regression coefficients of CD36^WT^ and CD36^G12V^ diffusion coefficient (A) and diffusion radius (B) on local CA speckle displacement, from MVRG analysis on speckle displacement and density (see Fig. S6 for corresponding regression coefficients on density). MVRG analysis was performed separately for the four indicated subgroups of SM tracklets based on local speckle density and diffusion type. In addition, next to the pictograms, +, ++, +++/−, --, and --- indicate significant differences (greater/smaller; P value ≤ 0.05, 0.01, and 0.001, respectively) between confined and free MVRG coefficient magnitudes at the same CA density level for each type of molecule. P value > 0.05 is not explicitly indicated to reduce clutter. **(C)** Local CA speckle displacement magnitude at CD36^WT^ and CD36^G12V^ molecules in the center and periphery regions. **(D and E)** Diffusion coefficient (D) and diffusion radius (E) of confined CD36^WT^ and CD36^G12V^ tracklets in the center and periphery regions. Circles, red lines, shaded bars, asterisks (or lack thereof), and pictograms (the latter in A and B) are as in Fig 2. See Table 5 for number of cells, number of experimental repeats, and number of SM tracklets and speckles per ROI used for analysis.

As expected (Dasgupta et al., 2023), the CD36^WT^ diffusion coefficient showed significantly negative MVRG coefficients on CA speckle displacement, with the coefficients of confined tracklets significantly stronger than those of free tracklets (Fig. 4 A and Fig. S6). The MVRG coefficients for diffusion radius paralleled those for diffusion coefficient (Fig. 4 B and Fig. S6). Compared with CD36^WT^, the MVRG coefficients of confined CD36^G12V^ tracklets were significantly weaker—at both low and high local speckle density in the case of the diffusion radius and at low local speckle density in the case of the diffusion coefficient (Fig. 4, A and B). In addition, the difference in coupling strength between confined and free tracklets was abolished in the case of the diffusion radius and reduced in the case of the diffusion coefficient at low local CA speckle density (Fig. 4, A and B). The MVRG coefficients for free tracklets were not affected by the mutation, probably because freely diffusing tracklets were less linked to CA in the first place.

Furthermore, differences in the diffusion properties of confined tracklets between center and periphery were abolished in the mutant. In the WT case, the diffusion coefficient and diffusion radius of confined tracklets in the center were significantly lower than their counterparts in the periphery, concomitant with the higher local CA speckle displacement in the center compared with the periphery (Fig. 4, C–E). These differences between center and periphery were consistent with the negative relationship between diffusion coefficient/radius and CA speckle displacement (Fig. 4, A and B [Dasgupta et al., 2023]). However, in the mutant case, although CD36^G12V^ experienced higher local CA speckle displacement in the center compared with the periphery like WT (Fig. 4 C), the diffusion coefficient and radius of its confined tracklets were similar between the two regions (Fig. 4, D and E).

The reduced coupling between CA dynamics and the diffusion properties of confined tracklets for CD36^G12V^ manifested in both reduced MVRG coefficients and a lack of difference in diffusion properties between the center and periphery, indicating a diminished role of CA in the confinement of CD36^G12V^ compared with CD36^WT^. This further supports the model that disrupting CD36 interactions with β_1_-integrin and tetraspanins weakens its coupling to CA, which implicates them in linking CD36 to CA.

### G12V mutation alters CD36 localization in caveolae and signaling at the PM

As CD36 lacks known signaling motifs, its organization at the PM is thought to be important to bring it together with its signaling partners, whether they be other cell surface receptors or intracellular signaling partners, such as SFKs (Chen et al., 2022; Githaka et al., 2016). CD36 signals through lipid- and protein-based PM domains, such as lipid rafts and caveolae (Febbraio et al., 2001), and through multimolecular complexes composed of integrins, tetraspanins, and other proteins (Chu et al., 2013; Heit et al., 2013; Kazerounian et al., 2011). With its altered dynamic organization, we hypothesized that CD36 signaling and function would be compromised by the G12V mutation.

**Figure 5.**
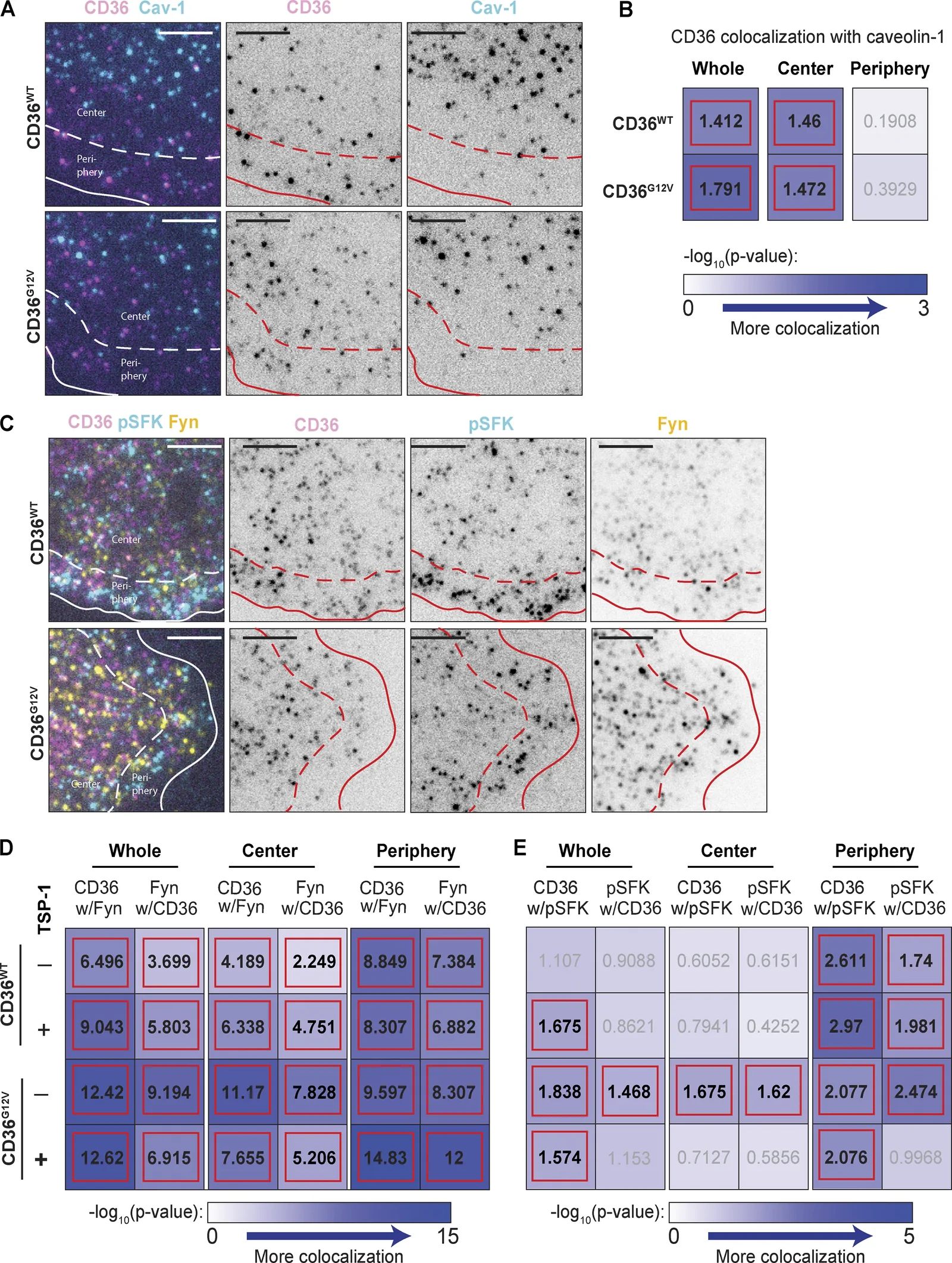
**G12V mutation increases CD36 localization at caveolin-1 structures and abolishes CD36 signaling in response to TSP-1. (A)** Representative two-color fixed-cell images of CD36 (magenta) and caveolin-1 (Cav-1) (cyan) on the surface of a TIME cell imaged via TIRFM, for CD36^WT^ (top) and CD36^G12V^ (bottom). Solid lines show the segmented cell edge, dashed lines specify the boundary between periphery and center regions. Scale bar, 5 μm. **(B)** −log_10_(P value) of test for significance of CD36 (WT or mutant) colocalization with Cav-1 puncta in the whole, center and periphery regions. Blue color intensity of each entry reflects level of significance, as shown in color bar below. Values in black and highlighted by a solid red outline are significant (P value ≤ 0.05; −log_10_(P value) ≥1.301), values in gray are not significant. **(C)** Representative three-color fixed-cell images of CD36 (magenta), pSFK(Y419) (cyan), and Fyn (yellow) on the surface of a TIME cell imaged via TIRFM for CD36^WT^ (top) and CD36^G12V^ (bottom). Solid and dashed lines and scale bars are as in A. **(D and E)** -log_10_(P value) of test for significance of CD36-Fyn colocalization (D) and of CD36-pSFK(Y419) colocalization (E), for WT and mutant CD36 in the whole, center, and periphery regions, in the absence or presence of TSP-1. Entry details are as in B. See Table 4 for number of cells, number of experimental repeats, and number of objects per channel per ROI used for analysis.

**Figure S8.**
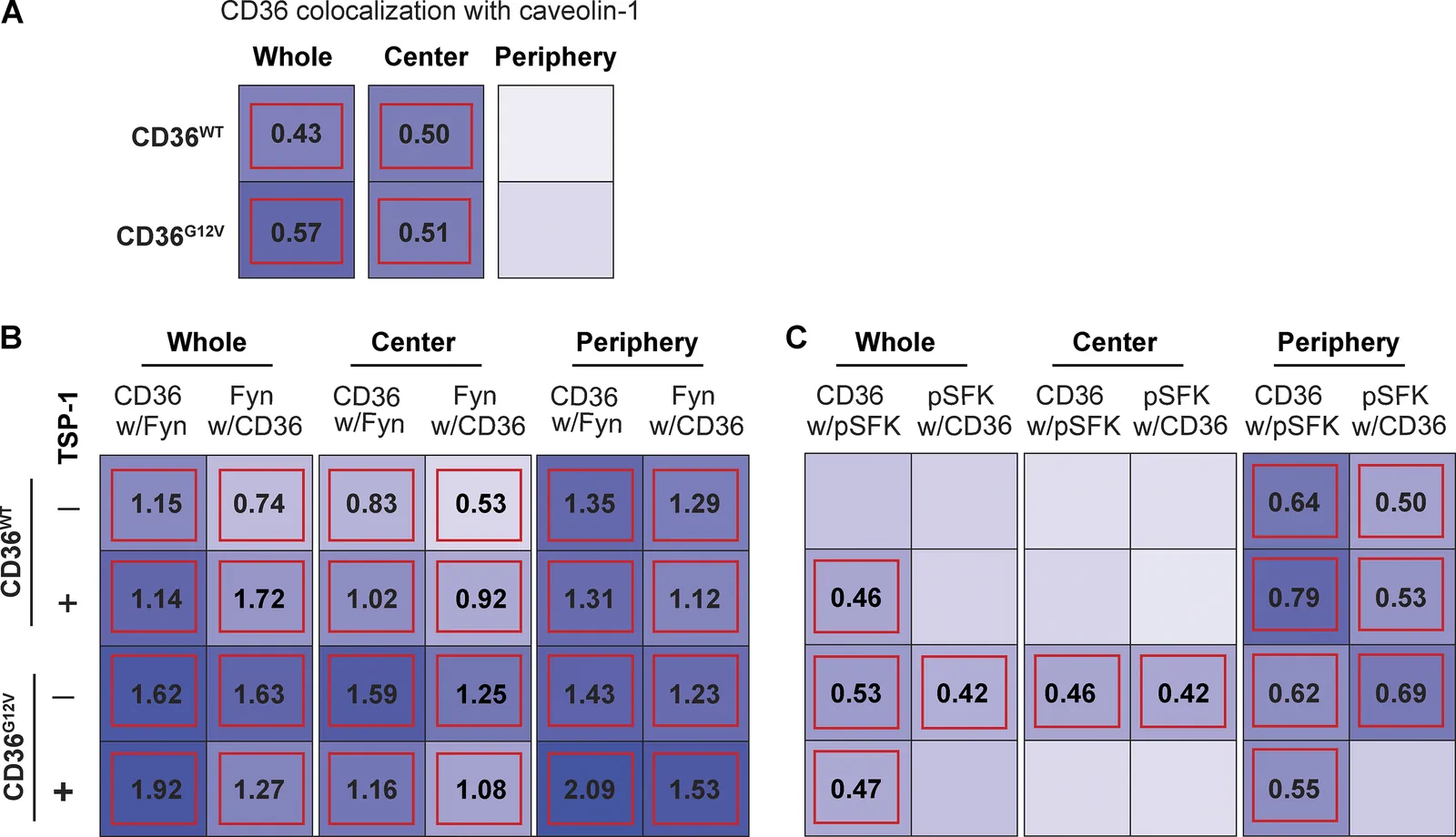
**Effect sizes (Cohen’s *d*) complementing the statistical testing P values of the colocalization experiments presented in Fig. 5. (A–C)** Cohen’s *d* comparing pairwise colocalization measures derived from experimental samples to their coincidental values (nullTR) for CD36-Cav-1 colocalization (A), CD36-Fyn colocalization (B), and CD36-pSFK colocalization (C). Blue color intensity of each table entry reflects the level of significance (P value) as described in Fig. 5. Cohen’s *d* is reported for only those cases where the colocalization is deemed significant via statistical testing (P value ≤ 0.05; as shown in Fig. 5), visually captured by darker shading. Empty entries thus belong to cases without significant colocalization, visually captured by light shading. Cohen’s *d* and statistical significance (-log_10_(P value)) overall correlate well with each other. Cav-1, caveolin-1.

CD36 localization in caveolae, which are highly abundant in vascular endothelial cells (Frank et al., 2003; Luse et al., 2023), has been shown to regulate various CD36 functions, such as fatty acid uptake, LDL endocytosis, and eNOS activation (Gerbod-Giannone et al., 2019; Peche et al., 2023; Uittenbogaard et al., 2000). To investigate whether the G12V mutation affected CD36 localization in caveolae, we co-imaged CD36 with caveolin-1 as a marker for caveolae in fixed cells (Fig. 5 A) and analyzed CD36 colocalization with caveolin-1 puncta. In the “whole” region analysis, CD36^G12V^ colocalization with caveolin-1 was higher than that of CD36^WT^ (Fig. 5 B and Fig. S8 A). However, for both WT and mutant, caveolin-1 was primarily localized in the center region of the PM (mean caveolin-1 density of 0.5 and 0.1 detections/μm^2^ in center and periphery, respectively). Given the redistribution of CD36^G12V^ toward the center (Fig. S1 H), we speculated that this played a role in its enhanced caveolin-1 colocalization. Separate center/periphery analyses confirmed that CD36 colocalization with caveolin-1 was significant only in the center for both WT and mutant, with only minor center-specific differences between mutant and WT (Fig. 5 B and Fig. S8 A). Thus, the higher overall CD36^G12V^–caveolin-1 colocalization was primarily due to the mutant’s redistribution toward the center. These changes upon mutating CD36 emphasize the important role that the dynamic organization of CD36–as mediated by its interactions with β_1_-integrin, tetraspanins, and CA—plays in regulating CD36 association with PM domains, such as caveolae, that in turn regulate CD36 function, such as internalization and signaling (Hao et al., 2020; Silverstein, 2025; Silverstein and Febbraio, 2009).

Next, we investigated whether CD36 signaling was perturbed by the G12V mutation. In MVECs, CD36 binding to its ligand TSP-1 leads to activation of the SFK Fyn (Dawson et al., 1997; Githaka et al., 2016; Jiménez et al., 2000). Therefore, we co-imaged CD36 with an antibody against phosphorylated tyrosine 419 in SFKs (Katasho et al., 2023; Peckham et al., 2016) as well as total Fyn (Fig. 5 C), either in unstimulated cells or in cells stimulated with TSP-1 (10 nM for 10 min). This imaging assay measured SFK activation specifically at CD36 molecules (via CD36-pSFK colocalization [Githaka et al., 2016]) in the context of center vs. periphery PM regions. As expected (Githaka et al., 2016), significant CD36^WT^-Fyn colocalization was present in the basal state, and it was enhanced by TSP-1 (Fig. 5 D and Fig. S8 B). It was stronger in the periphery than in the center, opposite to Fyn abundance (0.55 vs. 0.67 detections/μm^2^ in the periphery vs. the center). Fyn activation at CD36^WT^ (CD36^WT^-pSFK colocalization) also occurred primarily in the periphery (Fig. 5 E and Fig. S8 C), leading to a significant overall response driven by peripheral activation.

The effect of the mutation on CD36 colocalization with Fyn and on signaling was multifaceted. In contrast to WT, CD36^G12V^-Fyn colocalization in the basal state was stronger in the center than in the periphery (Fig. 5 D and Fig. S8 B). Additionally, there was an elevated level of SFK activation at CD36^G12V^ molecules in the center, unlike all other conditions (Fig. 5 E and Fig. S8 C, “Center”). Combined with peripheral Fyn activation, this led to high overall SFK activation at CD36^G12V^ molecules in the basal state (Fig. 5 E and Fig. S8 C). TSP-1 addition did not lead to further activation; rather, it reduced overall SFK activation at CD36^G12V^, abolishing it in the center and reducing it in the periphery (Fig. 5 E and Fig. S8 C). CD36^G12V^-Fyn colocalization reverted to being higher in the periphery, similar to WT (Fig. 5 D and Fig. S8 B). CD36^G12V^ in the presence of TSP-1 showed the highest CD36-Fyn colocalization yet the lowest Fyn activation, strongly indicating the impairment of CD36 signaling in response to TSP-1 due to the G12V mutation.

## Discussion

**Figure 6.**
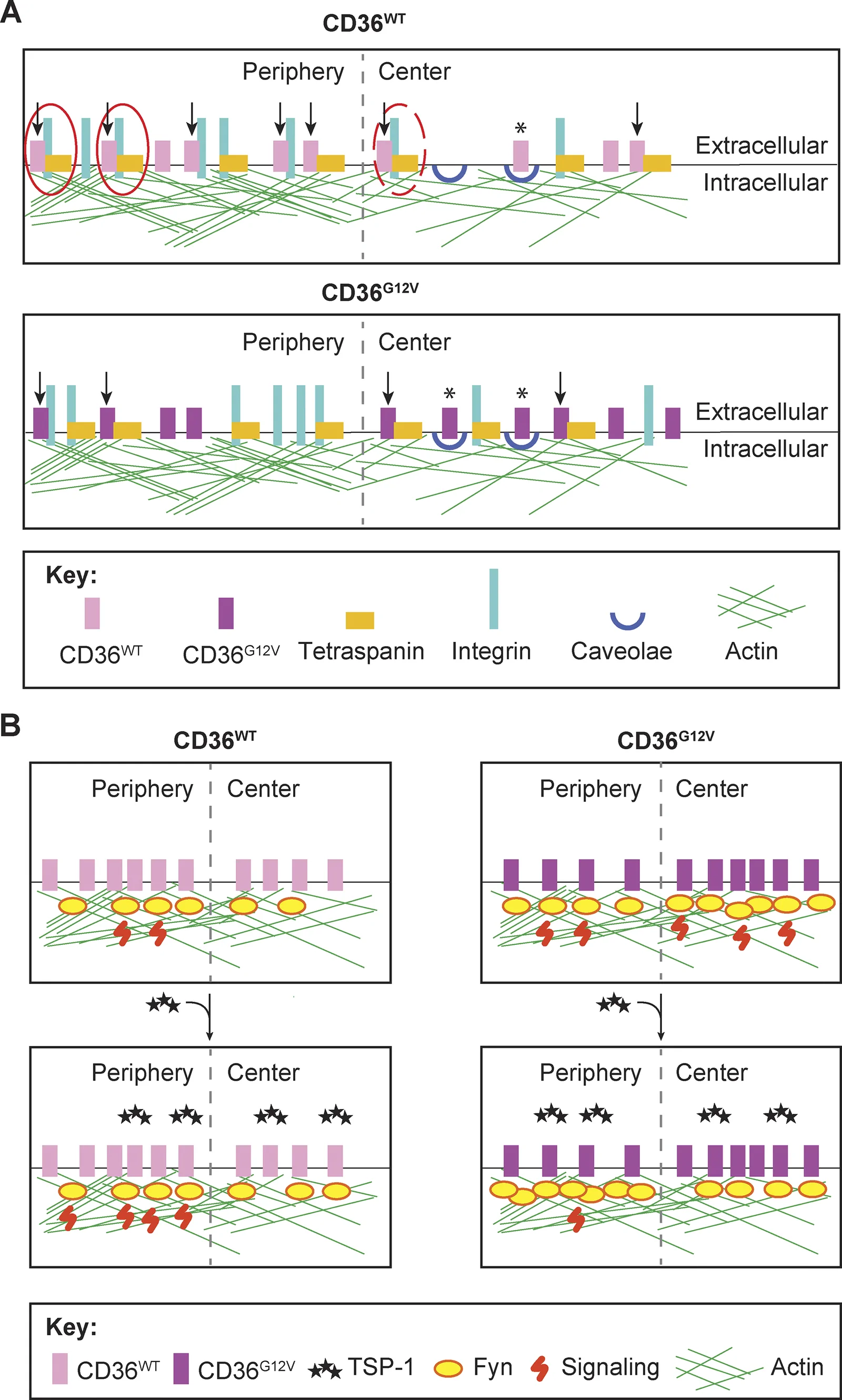
**Schematic models of CD36 localization, interactions, and coupling to CA, as well as signaling via the SFK Fyn. (A)** Illustration of the multi-faceted organization of CD36 at the PM of MVECs. In the WT case, CD36 is more abundant in the periphery region (<4 μm from the cell edge) than in the center region (>4 μm from the cell edge). In the periphery, CD36 associates with multimolecular assemblies containing integrins and tetraspanins, which in turn help link it to CA. In the center region, CD36 associates less with integrins and tetraspanins, and instead localizes more in caveolae. Disruption of CD36’s interactions with integrins and tetraspanins via the G12V point mutation (which weakens interactions and eliminates detectable CD36 association with integrin/tetraspanin multimolecular assemblies) weakens the link between CD36 and CA and shifts CD36 localization from the periphery to the center, leading to increased CD36 localization in caveolae. CD36 mobility also changes, where it exhibits more—but weaker—confinement, due to factors beyond CA (not shown in schematic). Arrows indicate that CD36 associated with integrins and tetraspanins can link to CA. Asterisks denote CD36 localized in caveolae (with probably altered link to CA). Solid/dashed ovals indicate the stronger/weaker three-way assemblies in the periphery/center. **(B)** Illustration of CD36-Fyn interactions and signaling in response to TSP-1. In the basal state, CD36^WT^ is significantly localized with Fyn, particularly in the periphery region, where there is weak tonic Fyn signaling. TSP-1 stimulation leads to stronger Fyn activation and signaling, but still only in the periphery. On the other hand, CD36^G12V^ displays elevated localization with Fyn in the center region, associated with strong Fyn signaling in the basal state. Furthermore, TSP-1 stimulation decreases Fyn activation rather than increasing it, abolishing it in the center and weakening it in the periphery. These results suggest that disruption of CD36’s interaction landscape and organization (including CD36-actin coupling) leads to aberrant, ligand-insensitive signaling and impaired regulatory control.

We have delineated components of the molecular link between the cell surface receptor CD36 and CA in MVECs by using quantitative in situ analysis of CD36 localization, interactions, mobility, and CA coupling along with a CD36 mutation. These components include β_1_-integrin and multiple tetraspanins, with interaction strength varying spatially with distance from the cell edge (Fig. 6 A). The weakened CD36 association with β_1_-integrin and CD9, as well as altered association with CD81 and CD151, by the G12V mutation (Fig. 1), reduce CD36–CA coupling (Fig. 2, Fig. 4, and Fig. 6 A) and are associated with changes in CD36 localization, mobility, and signaling (Figs. 2, 3, 5, and 6).

### Multimolecular assemblies including β_1_-integrin and various tetraspanins link CD36 to CA

To the best of our knowledge, this work is the first to provide evidence that β_1_-integrin and the investigated tetraspanins couple the dynamic organization of CD36 at the PM to CA, adding new function to multimolecular assemblies previously implicated in CD36 signaling (Heit et al., 2013; Kazerounian et al., 2011). The role of β_1_-integrin and CD9 in mediating the coupling is unambiguous: their colocalization and interactions with CD36 are consistently weakened across the PM for the G12V mutant, going hand-in-hand with reduced CD36–CA coupling. The roles of CD81 and CD151 are less straightforward because of the PM region–specific effect of the G12V mutation on their colocalization with CD36. Nevertheless, β_1_-integrin and these tetraspanins are expected to mediate the CD36–CA link collectively, through complexes and tetraspanin-enriched PM domains (Berditchevski, 2001; Charrin et al., 2009; Hemler, 2005; Schmidt et al., 2024).

In terms of CD36 distribution at the PM (Fig. 6 A), we found that CD36 localizes preferentially in the periphery, paralleling the distribution of its interaction partners and density of CA (Fig. 2 and Fig. S1). Disrupting the interactions of CD36 with β_1_-integrin and tetraspanins leads to a redistribution of CD36 away from the periphery (Fig. 2 and Fig. S1). Additionally, CD36 molecules colocalized with β_1_-integrin or CD9 appear to be in areas of higher CA density than CD36 molecules not colocalized with them (Fig. S3, B and C). The preferential localization of β_1_-integrin and the tetraspanins in the periphery might itself be due to the higher density of CA in the periphery, as all of these molecules link to CA (Brakebusch and Fassler, 2003; Sala-Valdés et al., 2006).

It is noteworthy that CD36 colocalization with its investigated interaction partners is stronger in the periphery (Fig. 1 and Fig. 6 A), suggesting that additional factors—other interaction partners, the lipid nano-environment, or CA itself—enhance and/or stabilize the interactions of CD36 with β_1_-integrin and tetraspanins in the periphery (Berditchevski et al., 2002; Charrin et al., 2003; Schmidt et al., 2024). Interestingly, the extent of colocalization between the different tetraspanins and between tetraspanins and β_1_-integrin is also overall stronger in the periphery. This raises the possibility that tetraspanins and integrins form stronger assemblies in the periphery (for the reasons mentioned above), into which CD36 is recruited more successfully. Alternatively, factors that disrupt the interactions of CD36 with β_1_-integrin and/or tetraspanins may be more active in the center.

A case at hand is the anticorrelation between the extent of CD36 colocalization with β_1_-integrin and tetraspanins (more in the periphery, less in the center) and the extent of CD36 localization in caveolae (exclusively in the center) (Fig. 1, Fig. 5, and Fig. 6 A). This is consistent with previous work showing that CD36 participates in at least two multimolecular assemblies, consisting of different proteins and lipids (Kazerounian et al., 2011). Of note, caveolae also link to actin (Echarri and Del Pozo, 2015), providing another mechanism for CD36–CA linking. But, being composed of different molecules, the strength and nature of this link is expected to be different from that provided by β_1_-integrin and tetraspanins.

In terms of CD36 mobility, our results suggest a multifaceted role for CA in regulating CD36 dynamics, probably because of the many links between CA and other factors that themselves regulate CD36 organization. As in our previous work (Dasgupta et al., 2023), our analyses here indicate that confined CD36^WT^ molecules exhibit stronger coupling to CA than freely diffusing CD36^WT^ molecules (Fig. 4). Confined CD36^WT^ molecules are also associated with higher-than-average CA density (Fig. S7). These observations implicate CA in confining CD36 PM movement, either directly or indirectly. Surprisingly, however, when the link between CA and CD36 is disrupted by the G12V mutation—as evidenced by reduced and/or insignificant MVRG coefficients for CD36^G12V^ MSS slope, diffusion coefficient, and diffusion radius on CA speckle displacement (Figs. 2 and 4)—CD36 mobility at the PM does not increase. Instead, a larger fraction of CD36 tracklets exhibits confined diffusion (Fig. 3), albeit with weaker confinement strength (Fig. 3 H). Importantly, the coupling of the confined tracklets to CA is lower in the mutant, becoming comparable to the coupling of free tracklets (Fig. 4). In addition, in contrast to confined CD36^WT^ molecules, confined CD36^G12V^ molecules are not associated with higher-than-average CA density (Fig. S7). These results indicate that confined motion of the mutant is (at least partly) distinct from that of CD36^WT^ and is due to different factors. As above, these factors could be lipid-based or other PM domains, or other interaction partners, many of which are expected to interact with CA.

### CD36 organization, mediated by multimolecular assemblies and CA, is important for CD36 signaling

A particular strength of imaging-based studies is that they reveal subcellular spatial information that is difficult to obtain otherwise. Here, we found that at least the initial steps of CD36 signaling upon binding its ligand TSP-1 are spatially compartmentalized, occurring primarily in the periphery region of the PM (Fig. 5 and Fig. 6 B). In both the absence and presence of ligand, CD36^WT^-Fyn colocalization is much stronger in the periphery than in the center, and CD36^WT^-pSFK colocalization, reflecting Fyn activation at CD36 molecules, occurs primarily in the periphery. The constitutive association of CD36 and Fyn and their weak basal-level signaling are consistent with previous work (Githaka et al., 2016). TSP-1 addition increases CD36-pSFK colocalization only in the periphery, indicating that Fyn activation upon CD36-TSP-1 binding occurs primarily in the periphery region, near the free cell edge. Similarly localized Fyn activation has been observed previously (Kostic and Sheetz, 2006; Mukherjee et al., 2020). It could increase the efficiency of CD36 signaling to regulate cell migration (Chu et al., 2013; Dawson et al., 1997).

There are multiple, potentially coexisting, mechanisms by which Fyn activation would occur primarily in the periphery. The stronger assemblies in the periphery, including stronger Fyn colocalization with CD36 (Fig. 5), could provide proper nanoscale organization and/or allow Fyn enough residence time to get activated upon CD36 binding to TSP-1 (Githaka et al., 2016; Hsieh et al., 2010; Mugler et al., 2012). Alternatively, additional interaction partners, present primarily in the periphery, and/or the lipid nano-environment in the periphery are necessary for Fyn activation. Furthermore, CA—which is denser in the periphery and has a different architecture—could play a role in Fyn activation (Sandilands et al., 2007). Distinguishing between these mechanisms requires elucidating the molecular basis of Fyn activation upon CD36 binding to TSP-1.

The G12V mutant helps shed light on some of these potential mechanisms. In the mutant, in both the center and the periphery, there is a decrease rather than an increase in CD36-pSFK colocalization upon TSP-1 addition, despite elevated levels of CD36-Fyn colocalization (Fig. 5 and Fig. 6 B). This speaks for a mechanism involving multiple molecular players to activate Fyn upon TSP-1 binding to CD36; Fyn colocalization with CD36 is not enough—other molecules, and potentially their nanoscale organization, partly mediated by CA, are needed (Githaka et al., 2016). Of note, the more striking difference in terms of CD36 colocalization with its interaction partners in the periphery between mutant and WT is not the reduction in colocalization per se, but the loss of colocalization interdependence in the mutant (Fig. 1), especially for β_1_-integrin and CD9. This points out the need for multimolecular assemblies for Fyn activation.

Curiously, the mutant shows a high level of basal-state CD36-pSFK colocalization in the center, which then drops upon ligand addition (Fig. 5 and Fig. 6 B). CD36^G12V^ in the basal state is also the only condition where CD36-Fyn colocalization is stronger in the center than in the periphery. It is possible that the increased colocalization of CD36^G12V^ with CD81 in the center (Fig. 1 M) allows CD36^G12V^ to engage with new molecules that bring it together with Fyn and mediate Fyn activation independently of TSP-1. It is also possible that the heavily reduced/abolished colocalization of CD36^G12V^ with β_1_-integrin, CD9, and/or CD151 in the center leads to a loss of downregulators of SFK activity in its vicinity, such as CSK (which interacts with integrins [Maldonado and Leyton, 2023; Obergfell et al., 2002]), resulting in increased Fyn activation. At the same time, TSP-1, which binds both CD36 and β_1_-integrin (Chen et al., 2000), may bring CD36^G12V^ and β_1_-integrin together and restore aspects of the WT nano-environment, thereby reducing Fyn overactivation. Yet peripheral Fyn activation in the mutant upon TSP-1 addition remains lower than WT, again highlighting the multimolecular requirements for Fyn activation. More broadly, the G12V mutant exhibits features of constitutive and ligand-insensitive signaling, highlighting how receptor mis-organization at the PM may decouple signaling from normal regulatory control, a principle that may extend to pathological contexts such as HER2-driven cancers, where altered nanoscale organization can limit the therapeutic disruption of HER2–Src complexes and contribute to drug resistance (Zhang et al., 2011).

In conclusion, our work identifies molecules (the β_1_-integrin subunit and the tetraspanins CD9, CD81, and CD151) and PM domains (tetraspanin-enriched domains and caveolae) that contribute to the molecular link between CD36 on the cell surface and CA. Perturbing the interactions between CD36 and its molecular partners is associated with altered CD36 dynamic organization and signaling. Our study highlights the multifaceted nature of PM-CA crosstalk, due to the involvement of multiple interacting and interdependent players. The players that we have identified—integrins, tetraspanins, and caveolae—interact with many PM components. Therefore, the principles learned from our study about the mechanisms linking CD36 to CA and their role in regulating CD36 dynamic organization and signaling are most likely translatable to many cell surface receptors.

## Materials and methods

### Cell lines and cell culture

Human telomerase-immortalized MVECs (TIME cells, ATCC) and TIME cells stably expressing low levels of mNeonGreen-actin (TIME-mNGrActin; [Dasgupta et al., 2023]), were used for this study. TIME cells at passage 15–55 and TIME-mNGrActin cells at passage 15–24 were grown in ATCC’s vascular cell basal medium supplemented with MVEC growth kit-VEGF (Catalog No. 50-238-2276), 12.5 µg/ml blasticidin (Catalog No. A11139-03; Thermo Fisher Scientific), and 0.1× of antibiotic-antimycotic (Catalog No. MT30004CI; Thermo Fisher Scientific) for 48 h at 37°C + 5% CO_2_ until reaching 80–90% confluency. At these passages, TIME cells express very little endogenous CD36, thus limiting CD36 expression to that from the transfected plasmid (Dasgupta et al., 2023). Early passage (P4-P7) primary human dermal MVECs isolated from neonatal foreskin (HDMECs, Lifeline Cell Technology) were grown in VascuLife VEGF-Mv culture medium for 48 h at 37°C + 5% CO_2_ until reaching 70–90% confluency. Whether for fixed-cell imaging (TIME cells or HDMECs) or live-cell SMI-FSM (TIME or TIME-mNGrActin cells), 7.4 × 10^4^ cells were seeded on fibronectin-coated (10 µg/ml, Millipore Sigma), base/acid cleaned, 0.17-mm (no. 1.5) glass bottom 35-mm dishes with 14-mm glass diameter (MatTek).

### Plasmids and mutagenesis

WT CD36 was fused to HaloTag at its N terminus (CD36^WT^) (Dasgupta et al., 2023). Starting with this plasmid, single-point mutations of the glycine (G) amino acid at the 12th or 16th position to valine (V) (CD36^G12V^, CD36^G16V^) and double-point mutation of the two positions (CD36^G12V/G16V^) were generated by site-directed mutagenesis. For this purpose, the CD36^WT^ plasmid was PCR-amplified with mutation-specific primers (Integrated DNA Technologies; see Table 1 for primer details) and cloned by In-Fusion seamless cloning (Catalog No.: 639648; Takara). The mutations were confirmed by sequencing (Fig. S4) (Plasmidsaurus, Oxford Nanopore Technology).

**Table 1.** CD36 mutation primers

Mutation	Forward primer	Reverse primer
CD36^G12V^	5′-TGT​TGC​TGT​CAT​TGG​TGC​TGT​CCT​GGC​TGT​GTT​TGG-3′	5′-CCA​ATG​ACA​GCA​ACA​GCG​ATG​AGC​CCA​CAG​TTC​C-3′
CD36^G16V^	5′-TGG​GGC​TGT​CAT​TGT​TGC​TGT​CCT​GGC​TGT​GTT​TGG-3′	5′-ACA​ATG​ACA​GCC​CCA​GCG​ATG​AGC​CCA​CAG​TTC​C-3′
CD36^G12V/G16V^	5′-TGT​TGC​TGT​CAT​TGT​TGC​TGT​CCT​GGC​TGT​GTT​TGG-3′	5′-ACA​ATG​ACA​GCA​ACA​GCG​ATG​AGC​CCA​CAG​TTC​C-3′

### Transient transfection of CD36

After 18 h of plating on fibronectin-coated glass-bottom dishes, TIME cells (for fixed-cell imaging) or TIME-mNGrActin cells (for live-cell SMI-FSM) were transfected with 0.25-μg/dish of WT or mutant HaloTag-fused CD36 plasmid (CD36^WT^, CD36^G12V^, CD36^G16V^, or CD36^G12V/G16V^) using TransfeX transfection reagent (ACS-4005; ATCC). The medium containing the transfection reagent was then replaced with complete culture medium 20 h after transfection. Imaging was performed 2 days after transfection. As transient transfection results in heterogeneous protein expression across cells, we selected cells with comparable overall CD36 labeling density during imaging to ensure consistency across experimental conditions and datasets.

### Sample preparation and labelling for fixed-cell imaging for colocalization analysis

For experiments involving CD36 (except for CD36/pSFK(Y419)/Fyn ± TSP-1; see below for details of these experiments), TIME cells transfected with a HaloTag-fused CD36 plasmid (WT or mutant) as described above were incubated for 15 min in complete culture medium containing 15 nM JF549-Halo ligand in the dark. For the CD36 surface expression experiments (Fig. S4), 6 nM JF650-Halo ligand was also added at the same time (although not used for subsequent analysis). Labeling with either Halo ligand was not saturating, but rather covered 20–30% of CD36 molecules. The cells were then given a quick wash with sterile DPBS, followed by 15-min incubation with dye-free complete culture medium. All incubations were performed at 37°C + 5% CO_2_. The cells were then washed three times (quick washes) with sterile DPBS. Then, the cells were fixed with 4% paraformaldehyde (PFA) solution made in PBS (Electron Microscopy Sciences) for 15 min at RT, followed by three washes (5 min each) with wash buffer (HBSS +0.1% normal goat serum [NGS]). For experiments not involving CD36 (three-color imaging of CD9/CD81/CD151), TIME cells were plated on fibronectin-coated glass-bottom dishes for 18 h, and then, without CD36 transfection, they were fixed as described above.

For the CD36/pSFK (Y419)/Fyn ± TSP-1 experiment, the cells were serum-starved for 3 h. After about 2 h of starvation, CD36 (WT or mutant) was labelled with 15 nM JF549-Halo ligand as described above, but in serum-free medium. Then the samples were exposed to either 10 nM TSP-1 in HBSS (+TSP-1) or HBSS alone (-TSP-1, control) for 10 min at 37°C. The cells were then fixed with 4% PFA +0.1% glutaraldehyde for 20 min at 4°C, followed by quenching (0.1% NaBH_4_ in HBSS) for 7 min at RT, and then three washes (5 min each) with washing buffer (HBSS +0.1% NGS).

After fixation, there were one or two rounds of primary + secondary (where needed) antibody labeling, depending on the combination of antibodies and their permeabilization requirements (see Table 2 for details). Each round of labeling was preceded by sample blocking for 15 min or 30 min in blocking buffer (3% BSA and 5% NGS in HBSS). Also, antibodies for labeling, as well as phalloidin, were diluted in blocking buffer. All blocking and labeling were done at RT. The samples were washed three times (5 min each) with wash buffer after each antibody labeling step (whether primary or secondary). Where required, the samples were permeabilized with ice-cold 0.1% Triton X-100 in PBS for 1 min, followed by labelling with antibodies that bind to an intracellular target. Sample permeabilization was always done after labeling all targets accessible without permeabilization. After all rounds of labeling, cells were incubated in imaging buffer (OxyFluor 1%, glucose 0.45%, and Trolox 2 mM) to reduce photobleaching before and during imaging. Labeling details are in Table 2.

**Table 2.** Labeling details for fixed-cell imaging

	Permeabilization	Round 1 of labeling			Round 2 of labeling		
		Blocking (min)	Primary antibody	Secondary antibody	Blocking (min)	Primary antibody	Secondary antibody
CD36 Halo-JF549/β_1_-integrin/CD9	Yes After CD36 and β_1_-integrin Before CD9	30	Anti–β_1_-integrin K20 (IMBULK1B; Beckman Coulter) 1:10,000 1 h	Anti–mouse-AF488 (A-11029; Thermo Fisher Scientific) 1:1,000 15 min	30	Anti–CD9-AF647 (sc-13118) 1:25 30 min	-
CD36 Halo-JF549/β_1_-integrin/CD81	No	30	Anti–β_1_-integrin K20 1:100,000 1 h	Anti–mouse- AF488 1:1,000 15 min	30	Anti–CD81-AF647 (sc-23962) 1:300 30 min	-
CD36 Halo-JF549/β_1_-integrin/CD151	No	30	Anti–β_1_-integrin K20 1:100,000 1 h	Anti–mouse-AF488 1:1,000 15 min	30	Anti–CD151-AF647 (ab199200) 1:250 30 min	-
CD9/CD81/CD151	Yes After CD81 and CD151 Before CD9	30	Anti–CD151-AF647 1:250 and Anti–CD81-AF488 1:100 Both for 30 min	-	30	Anti–CD9-AF546 1:200 30 min	-
CD36 Halo-JF549/CD36 Halo-JF650/anti-CD36 FA6-152 Note: CD36 Halo-JF650 not used in analysis	No	15	Anti–CD36 FA6-152 1:400 30 min	Anti–mouse-AF488 1:1,000 15 min	-	-	-
CD36 Halo-JF549/anti-CD36 FA6-152/caveolin-1 Note: Anti-CD36 FA6-152 not used in analysis	Yes After CD36-Halo and anti-CD36 FA6-152 Before caveolin-1	15	Anti-CD36 FA6-152 (ab17044) 1:400 30 min	Anti–mouse-AF488 1:1,000 15 min	30	Anti-caveolin-1 (ab32577) 1:200 30 min	Anti–Rabbit-AF647 (Invitrogen, A32733) 1:1,000 15 min
CD36 Halo-JF549/pSFK(Y419)/Fyn	Yes After CD36 Before pSFK(Y419) and Fyn	15	Anti–phospho-Src (Tyr419)-AF488 (44–660A1; Thermo Fisher Scientific) 1:500 & Anti-Fyn (ab1881) 1:400 Both for 1 h	Anti–mouse-AF647 (A21236; Thermo Fisher Scientific) 1:1,000 15 min	-	-	-
CD36 Halo-JF549/caveolin-1/phalloidin Note: Only phalloidin channel used from this dataset	Yes After CD36 Before caveolin-1 and phalloidin	30	Anti-caveolin-1 1:200 30 min	Anti–Rabbit-AF647 1:1,000 15 min	15	Phalloidin-AF647 (A22287; Thermo Fisher Scientific) 1:20 15 min	
CD36 Halo-JF549/active β_1_-integrin/CD9 Note: CD9 not used in analysis	Yes After CD36 and active β_1_-integrin Before CD9	60	Anti- active β_1_-integrin 12G10 (ab30394; Abcam) 1:500 1 h	Anti–mouse-AF488 1:1,000 15 min	30	Anti–CD9-AF647 1:25 30 min	
CD36 Halo-JF549/β_1_-integrin/phalloidin	Yes After CD36 and β_1_-integrin Before phalloidin	30	Anti–β_1_-integrin K20 1:100,000 1 h	Anti–Rabbit-AF488 1:1,000 15 min	15	Phalloidin-AF647 1:200 15 min	
CD36 Halo-JF549/CD9/phalloidin	Yes After CD36 Before CD9 and phalloidin	30	Anti–CD9-AF647 1:25 and Phalloidin-AF488 (A12379; Thermo Fisher Scientific) 1:500 30 min				

### Fixed-cell imaging for colocalization analysis

After labeling, the cells were imaged at 37°C using an Olympus IX83 TIRF microscope equipped with a Z-Drift Compensator and a UAPO 100×/1.49 NA oil-immersion TIRF objective (Olympus). The microscope was equipped with an iXon 888 1k × 1k EMCCD Camera (Andor; Oxford Instruments). With an additional 1.6× magnification in place, the pixel size in the recorded image was 81 nm × 81 nm. Using the Olympus cellSens software, excitation light of 640, 561, and 491 nm from an Olympus CellTIRF-4Line laser system was directed to the sample by a TRF8001-OL3 Quad-band dichroic mirror. Fluorescence of different wavelengths was collected, filtered with emission filters of ET520/40m, ET605/52m, and ET705/72m (Chroma), and projected onto different sections of the camera chip by an OptoSplit III 3-channel image splitter (Cairn Research). The different channels were excited and recorded sequentially in the order 640, then 561, then 491, with an exposure time of 99 ms each. Images were acquired with MetaMorph (Molecular Devices). Camera EM gain was set to 100 for all acquisitions. The penetration depth was set to 90 nm via the cellSens software (Olympus).

For every three-channel image, a bright-field snapshot of the imaged cell region was also acquired to aid with manual delineation (if needed) of the region of interest (ROI) mask for the ensuing analysis.

Laser powers for the different fixed-cell experiments are given in Table 3.

**Table 3.** Laser powers at sample position (widefield illumination configuration)

Experiment/Condition	491 nm (mW)	561 nm (mW)	640 nm (mW)
1. All experiments except for the cases listed on lines 2–4	2.4	7.06	2.0
2. CD36^WT^/active β_1_-integrin	2.4	8.8	4.0
3. CD36^WT^/β_1_-integrin/phalloidin	2.4	8.8	3.0
4. CD36^WT^/CD9/phalloidin	1.2	8.8	4.0

### Bead preparation and imaging for registration shift correction in fixed cells

To acquire images of TetraSpeck beads (T7279; Thermo Fisher Scientific) for calculating the registration shift between the 3 channels in fixed cell images (491, 561, and 640), TetraSpeck beads were suspended (1:100) in distilled water using a sonicator-water bath for 15 min. The mixed bead sample was then combined with 10 μl poly-L-Lysine (Newcomer Supply) diluted 1 in 10 μl in nuclease-free water, and then plated on a cleaned MatTek dish (as used for cells but without fibronectin coating) for 30 min at RT in the dark. The bead sample was imaged using the Olympus IX83 TIRF microscope described above. The penetration depth was set to 90 nm via the cellSens software (Olympus). Laser powers at the sample position (in the widefield illumination configuration) were 0.62, 1.76, and 1.43 mW for the 491, 561, and 640 nm lasers, respectively.

### Halo-tag labeling of CD36 for live-cell SMI-FSM

TIME-mNGrActin cells, transfected with WT or mutant CD36 as described above, were incubated for 15 min in complete culture medium with 3 nM JF549-Halo ligand (∼2 days after transfection). Incubation with dye was followed by three quick washes in sterile DPBS. Cells were then incubated for 15 min in dye-free complete culture medium. Incubations were at 37°C + 5% CO_2_. Finally, cells were washed three times (5 min each) with wash buffer (HBSS +1 mM HEPES and 0.1% NGS). Cells were then incubated in imaging buffer (OxyFluor 1%, glucose 0.45%, and Trolox 2 mM) to reduce photobleaching before and during imaging.

### Live-cell SMI-FSM data acquisition

Live TIME-mNGrActin cells expressing CD36^WT^ or CD36^G12V^, both fused to HaloTag and labeled with JF549-Halo ligand as described above, were imaged at 37°C using the Olympus IX83 TIRF microscope described earlier. The videos were acquired with MetaMorph in the stream acquisition mode, with the two channels acquired simultaneously, using the trigger function to control illumination. The 561 channel was used for acquiring SMI videos. These were acquired at 10 Hz for 50 s (501 frames). The 561 channel was triggered to remain open (i.e., continuous illumination) for the entire 501 frames. Simultaneously, the 491 channel was used for acquiring FSM videos. While the 491 channel was acquired also at 10 Hz, the 491 channel was triggered to illuminate the sample only every 50th frame (for 99 ms each). In addition, image acquisition in the 491 channel started 500 frames (50 s) before the start of the 561 channel image acquisition and continued for 500 frames (50 s) after the end of the 561 channel image acquisition (all while illuminating only every 50^th^ frame). Removing the “empty” 491 channel frames (those in the absence of 491 illumination) (using MetaMorph, after video recording) resulted in an FSM time-lapse at 0.2 Hz for 31 FSM frames (150 s), with the middle 11 FSM frames (50 s) corresponding to the 501 SMI frames. The additional FSM frames before and after the simultaneous SMI-FSM period extended the time window of FSM imaging to capture the birth and death of most actin speckles that got matched to SM tracklets in the middle 11 FSM frames, thus allowing accurate speckle lifetime measurement (88% of speckles with known birth and death times). Speckle lifetime reflects the stability of the observed CA network (longer lifetime = more stable network). Temperature and humidity were maintained during imaging using an environmental chamber (Okolab), maintaining cell viability for the duration of the experiments. The penetration depth was set to 80 nm via the cellSens software (Olympus). Laser powers at the sample position (in the widefield illumination configuration) were 3.08 mW and 7 mW for the 491 and 561 nm laser lines, respectively. Every SMI-FSM movie was preceded by a bright-field snapshot of the imaged cell region to visually check cell viability and to aid with manual delineation (if needed) of the ROI mask for the ensuing analysis.

### Cell segmentation (whole, center, and periphery)

In fixed-cell experiments, the cell ROI masks were segmented manually based on the bright-field images and β_1_-integrin channel if available, except in cases where actin was labeled with fluorescent phalloidin. In those cases, ROI masks were segmented automatically based on the phalloidin intensity using the u-segment package (https://github.com/DanuserLab/u-segment [Lee et al., 2015; Noh et al., 2022]). This applied to two experiments. In the experiments to analyze actin density differences between the cell periphery and cell center (Fig. S1 B), the automatic threshold was selected to segment the whole imaged part of a cell. In the experiments to analyze actin enrichment around CD36 colocalized or not with β_1_-integrin or CD9 (Fig. S3, B and C), the automatic threshold was selected to restrict the analysis to the area of the cell with relatively high, contiguous phalloidin signal (i.e., the segmented area corresponded roughly to the periphery region). In live-cell experiments, the cell ROI masks were generated either within the quantitative FSM (QFSM) package at the thresholding and mask refinement steps (steps 2 and 3) or they were hand-drawn if thresholding failed (Dasgupta et al., 2023). Starting with these “whole” ROI masks, the center ROI masks were created by eroding the whole ROI masks with a disk-shaped structuring element of radius 50 pixels (4.05 µm). The “periphery” ROI masks were then created by subtracting the center masks from the whole masks. This process resulted in center and periphery regions being roughly >4 µm and <4 µm, respectively, from the segmented cell edge.

### Punctate object detection in fixed cells and TetraSpeck beads

Punctate objects in fixed-cell and TetraSpeck bead experiments were detected using the “point-source detection” particle detection algorithm in u-track (https://github.com/DanuserLab/u-track) (Aguet et al., 2013; Jaqaman et al., 2008). In brief, the algorithm consists of two steps: (1) a convolution/filtering step to determine pixels likely to contain objects and (2) a Gaussian fitting step to determine the object positions with subpixel localization. With the appropriate, wavelength-dependent standard deviation (1.2, 1.35, and 1.58 pixels for the 491, 561, and 640 channels, respectively), a 2D Gaussian is a good approximation of the microscope’s point spread function (Thomann et al., 2002; Zhang et al., 2007). Largely default parameter values were used, except in some cases the α-value for determining the significance of detected objects (by comparing the fitted Gaussian amplitude with the local background noise distribution), which was chosen based on visual assessment of the detection results with the goal of minimizing false positives (superfluous detections) and false negatives (missed particles). The default α-value was 0.05, and following are the nondefault cases: β_1_-integrin (491 channel in all experiments), 0.01; CD81 (491 channel in the CD9/CD81/CD151 experiment), 0.01; caveolin-1 (640 channel), 0.1; and TetraSpeck beads—561 channel, 10^−6^ and 491 and 640 channels, 0.001). The number of objects detected within the ROI for the colocalization analysis experiments from the fixed-cell datasets is listed in Table 4.

**Table 4.** Dataset size with channel details, number of cells, and number of repeats per experiment, including average number of objects in each channel per ROI

Dataset (Channels 1/2/3, i.e., 561/491/640)	No. of cells	No. of experimental repeats	Channel 1 objects per ROI (average)			Channel 2 objects per ROI (average)			Channel 3 objects per ROI (average)		
			Whole	Center	Periphery	Whole	Center	Periphery	Whole	Center	Periphery
CD36^WT^/β_1_-integrin/CD9	32	4	452	317	135	494	296	199	445	304	142
CD36^G12V^/β_1_-integrin/CD9	36	4	385	293	93	567	350	217	378	257	121
CD36 ^WT^/β_1_-integrin/CD81	48	6	449	321	136	491	304	184	421	293	128
CD36^G12V^/β_1_-integrin/CD81	48	6	377	272	105	523	334	189	377	253	124
CD36^WT^/β_1_-integrin/CD151	32	4	363	232	130	606	363	243	328	193	136
CD36^G12V^/β_1_-integrin/CD151	34	4	315	219	96	598	342	256	362	209	153
CD9/CD81/CD151	32	4	396	311	85	434	317	116	316	229	87
CD36^WT^/caveolin	36	4	556	424	132	-	-	-	426	406	20
CD36^G12V^/caveolin	36	4	574	444	130	-	-	-	424	395	29
CD36^WT^/pSFK(Y419)/Fyn, - TSP1	50	6	490	356	134	613	442	173	517	396	121
CD36^WT^/pSFK(Y419)/Fyn, +TSP1	50	6	518	375	143	689	506	184	550	426	124
CD36^G12V^/pSFK(Y419)/Fyn, - TSP1	50	6	422	320	103	575	413	162	475	363	113
CD36^G12V^/pSFK(Y419)/Fyn, +TSP1	50	6	458	365	93	627	479	149	517	414	104
CD36^WT^/active β_1_-integrin	32	4	583	407	176	1,170	833	337	-	-	-

### Comparison of CD36 expression levels between endogenous and transient overexpression

To quantify CD36 levels under transient expression conditions, TIME cells transfected with Halo-CD36^WT^ (and labeled with 15 nM Halo ligand as described in the “Sample preparation and labelling for fixed-cell imaging” section, although not used for analysis) were used. To quantify endogenous CD36 levels, early-passage HDMECs (P4-P7) (primary MVECs) were used. Samples from both conditions were fixed with 4% PFA made in PBS for 15 min at RT. Samples were then blocked for 15 min in blocking buffer, followed by incubation with the anti-CD36 antibody FA6-152 (1:400, Abcam) for 1 h at RT. After three washes, samples were incubated with Alexa Fluor 488–conjugated secondary antibodies (1:1,000, Thermo Fisher Scientific) for 15 min at RT. Finally, after three subsequent washes, dishes were incubated with imaging buffer to reduce photobleaching. Immunofluorescence (IF) imaging for endogenous expression in HDMECs was then performed using the S-TIRF microscope described by Dasgupta et al. (2023), using a 488 nm laser power of 2.6 mW at the sample position. IF imaging for transient overexpression in TIME cells was performed using the IX83 TIRF microscope described in the “Fixed-cell imaging for colocalization analysis” section above, using a 491 nm laser power of 2.4 mW at the sample position. A bright-field snapshot of the imaged cell region was also acquired to aid with manual delineation of the ROI to obtain cell masks for the ensuing analysis. For both datasets, CD36 puncta were detected using the detection pipeline described in the section “Punctate object detection in fixed cells and TetraSpeck beads”. Detection densities were then calculated and compared between endogenous and overexpression conditions using a two-sample *t* test.

### Registration shift and channel alignment

To increase the accuracy and specificity of the ensuing colocalization analysis, the coordinates of detected objects in the three channels were aligned relative to each other. This was achieved by calculating a registration shift between the three channels, using TetraSpeck bead images acquired on the same day or, at most, in the same week. While in the OptoSplit III setup the registration shift between channels was primarily a constant translation across the imaged area, the TetraSpeck beads density was high enough to allow us to calculate the registration shift as a function of (x,y)-coordinates, increasing the accuracy of the registration shift correction. In general, the registration shift between channels was stable and varied very little from week to week. With the registration shift, the alignment between channels was improved from 2-6 pixels (before registration shift) to 0-2 pixels (after registration shift).

In brief, the TetraSpeck bead detections in the three channels were matched between channels using the linear assignment problem (Jaqaman et al., 2008), using a search radius of 9 pixels between the 491 and 561 channels, and a search radius of 5 pixels between the 640 and 561 channels. The 561 channel was taken as the reference. The matchings from multiple images acquired on the same day (3–5 images) were then utilized together to increase imaged area coverage. Using the matched bead detections, the registration shifts in x and y (Δx and Δy) as a function of position (x and y) were fit using a quadratic polynomial, deemed visually as the most appropriate fit. With this fit, the detections in the fixed-cell multichannel images were then aligned prior to colocalization analysis.

### Conditional colocalization analysis in different PM ROIs

Conditional colocalization analysis of the fixed-cell images was performed as described by Vega-Lugo et al. (2022). In brief, conditional colocalization analysis quantifies the colocalization of one molecular entity (target) with another molecular entity (reference) and how much this colocalization changes based on either’s colocalization with a third molecular entity (condition). As the residual registration shift between channels in the beads experiment after channel alignment was 0–2 pixels (described above), we used a colocalization radius of two pixels (=162 nm) in our analysis. Colocalization analysis was done separately for the whole, center, and periphery ROI masks. For each analysis, only the area of the ROI was used for any randomization and other procedures to assess the significance of the observed colocalization measures (Helmuth et al., 2010; Vega-Lugo et al., 2022).

### Statistical testing for colocalization analysis

All statistical testing was as described previously, with the significance of colocalization shown in the form of -log_10_ (P value) (Vega-Lugo et al., 2022). The P value for assessing significance was calculated as follows, with all tests being a Wilcoxon rank-sum test:

For p(TwR) (two-way colocalization), the data p(TwR) was compared with its corresponding nullTR, which was obtained by replacing target objects with points on a grid. NullTR reflected the coincidental colocalization fraction in the absence of any true relationship between the target and reference molecules. It depended on the density and spatial distribution of the reference objects (Helmuth et al., 2010). Thus, this comparison assessed whether the observed colocalization of target with reference was more than expected by chance. The significance threshold for p(TwR) was set to 0.05.

For P(TwR|TwC), three comparisons were performed:

(i) Comparison with its corresponding nullTR. As with p(TwR), this comparison assessed whether the observed colocalization of the condition-positive target subset with the reference was more than expected by chance.
(ii) Comparison with its corresponding randC, which was obtained by randomizing the condition objects (repeated 100 times). When the condition objects were randomized, the resulting “condition-positive target subset” was purely a randomly selected subset of the full population. Thus, this comparison assessed whether the real data condition-positive target subset was truly different from the general target population.
(iii) Comparison with p(TwR). This comparison assessed whether the colocalization extent of the condition-positive target subset with the reference was statistically different from the colocalization extent of the general target population with the reference.

For p(TwR|TwC) to be significant, the P value for each of these tests had to be <0.017 (using the Dunn–Sidak correction to obtain a total type-I error of 0.05 for the three tests). Note that the reported P value in Table S1 and the figures is the largest (i.e., least significant) of these three P values.

For p^rs^(Tw(RwC), the same three comparisons as above were performed:

(i) Comparison with its corresponding nullTR. As above, this comparison assessed whether the observed colocalization of target with the condition-positive reference subset was more than expected by chance.
(ii) Comparison with its corresponding randC. Analogous to above, this comparison assessed whether the real data condition-positive reference subset was truly different from the general reference population.
(iii) Comparison with p(TwR). This comparison assessed whether the colocalization extent of target with the condition-positive reference subset was statistically different from the colocalization extent of target with the general reference population.

Of note, to enable the above comparisons for the condition-positive reference subset, the conditional colocalization measure p^rs^(Tw(RwC) was a rescaled probability (thus the superscript “rs” in the name). I.e., p^rs^(Tw(RwC) was not a fraction (as was the case for P(TwR) and P(TwR|TwC)), but rather a fraction rescaled to compensate for the different number of reference objects resulting from selecting only those reference objects that were colocalized with the condition. The rescaling was necessary to put the measures to be compared with each other in the above tests on equal footing to make their comparison meaningful (Vega-Lugo et al., 2022).

In addition to the above tests to assess significance, for the conditional colocalization experiments in Fig. 1 and the colocalization experiments in Fig. 5, the effect size comparing the conditional colocalization measures or pairwise colocalization measures derived from experimental data to their respective pairwise colocalization measure or coincidental counterparts was also calculated, using the “robustcohen” algorithm (MATLAB function meanEffectSize ()), which calculates Robust Cohen’s *d* for two samples. The 95% confidence interval was obtained from 3,000 bootstrap samples. The mean effect size is reported in Table S1 and Fig. S8 for the indicated experiments.

### Analysis of actin enrichment at CD36 colocalized or not colocalized with β_1_-integrin or CD9

To quantify the level of actin enrichment at CD36 detections colocalized, or not colocalized, with β_1_-integrin (or CD9), first CD36 and β_1_-integrin (or CD36 and CD9) were detected in the fixed cell images of CD36^WT^/β_1_-integrin/phalloidin (or CD36^WT^/CD9/phalloidin), as described in the section Punctate object detection in fixed cells and TetraSpeck beads. Each CD36 detection was then classified as either colocalized or not with β_1_-integrin (or CD9) based on its distance to the nearest β_1_-integrin (or CD9) detection. Specifically, if its nearest β_1_-integrin (or CD9) detection was closer than the colocalization radius of two pixels, then a CD36 detection was considered colocalized with β_1_-integrin (or CD9) (this is essentially the first step of distance-based colocalization analysis [Vega-Lugo et al., 2022]). With this, the CD36 detections were divided into 2 groups: 1 group colocalized with β_1_-integrin (or CD9) and 1 group not colocalized with β_1_-integrin (or CD9).

Next, actin enrichment analysis was performed for each group as described in the “Intensity enrichment analysis” section of (Githaka et al., 2016). In brief, the local actin intensity around each CD36 detection (after background subtraction) was calculated using a circular area of radius three pixels around each detection, these local actin intensities were averaged for the CD36 detections in each group (i.e., colocalized or not with β_1_-integrin [or CD9]), and then the average local intensity for each group was divided by the global average actin intensity outside of the CD36 detections (i.e., outside the three pixel radius around each detection, after background subtraction) (Githaka et al., 2016). Subtracting 1 from this ratio yielded a local actin enrichment metric with values ∼0, >0, and <0 when the local actin intensity around CD36 detections was, respectively, similar to, greater than (i.e., enrichment), or less than (i.e., depletion) the global average actin intensity. The analyzed area per cell was restricted to regions of uniformly detectable actin intensity as described in the “Cell Segmentation” section of this article (also see cell masks in Fig. S3, B and C). As each cell provided an actin enrichment value for both groups of CD36 detections (colocalized and not colocalized with β_1_-integrin [or CD9]), a paired-sample *t* test was performed to compare actin enrichment values between CD36 detections colocalized with β_1_-integrin (or CD9) and those not colocalized with β_1_-integrin (or CD9).

### Live-cell SMI-FSM analysis

SM tracks were constructed from the SMI streams using u-track (version 2.2.1; https://github.com/DanuserLab/u-track) (Jaqaman et al., 2008). The SM detection and tracking parameters were identical to those used previously (Dasgupta et al., 2023). The tracks output by u-track were then processed to remove artifactual merging and splitting events (in addition to those described previously [Dasgupta et al., 2023], very short merge-to-split events lasting ≤ 2 frames were eliminated as well) and to discard short tracks (duration <10 time points) and tracks outside the cell mask. SM tracks were then further processed into tracklets localized in space and time and in terms of diffusion type for matching with CA speckles (Dasgupta et al., 2023). SM tracklets’ mobility was analyzed with both DC-MSS analysis (Vega et al., 2018) and diffusion mode analysis (da Rocha-Azevedo et al., 2020; Jaqaman et al., 2016), although in this work only the DC-MSS results were used. Properties characterizing each SM tracklet were obtained, including (as used in this work):

• Diffusion MSS slope (characterizes diffusion type).
• Diffusion type (free, confined, immobile, or directed).
• Diffusion coefficient.
• Diffusion radius (describes the area covered by the SM tracklet during its lifetime; equivalent to confinement radius for confined SMs).
• Confinement strength, derived from the scaling exponent of the mean square displacement ($\langle r^{2}\left(\tau \right)\rangle$) following (Doliwa and Heuer, 1998). The confinement strength was calculated as $c=1-4^{\left(\alpha -0.5\right)}$, where α represents the scaling of the root mean square displacement, $\sqrt{\langle r^{2}\left(\tau \right)\rangle }\propto \tau^{\alpha }$. For pure Brownian SM, α=0.5 leading to c=0; for immobile SM, α=0 leading to c=0.5.

On the other hand, FSM time-lapse sequences were analyzed using the QFSM software package (https://github.com/DanuserLab/QFSM) (Danuser and Waterman-Storer, 2006) following the instructions by Mendoza et al. (2012), and processed to remove “ghost speckles” and artifactual movement, as done previously (Dasgupta et al., 2023). Individual CA speckle properties (e.g., position, displacement, intensity, and lifetime) were then calculated from the speckle tracks output by QFSM software (Dasgupta et al., 2023). After matching CA speckles to SM tracklets temporally and spatially, we calculated the local speckle properties around each SM tracklet by averaging the individual speckle properties (Dasgupta et al., 2023).

For quantifying the mathematical dependence of SM tracklet properties on the matched local speckle properties, linear MVRG (using the MATLAB function mvregress()) was performed on an individual cell/ROI (movie) basis. SM tracklet properties were taken as the response (“dependent”) variables, and the associated local speckle properties were taken as the design matrix (“independent variables”). Each property was normalized by its sample mean and standard deviation. MVRG was performed only for complete datasets (SM tracklets with locally matched speckles) with a minimum of 11 data points. MVRG coefficients from individual cells within each experimental condition were grouped for statistical significance testing and comparison across conditions. Outliers were identified using the MATLAB function isoutlier (data, “gesd”), which detects multiple outliers in approximately normally distributed data (Dasgupta et al., 2023). The number of SM tracklets and speckle tracks within the ROI for each live-cell SMI-FSM dataset is listed in Table 5.

**Table 5.** SMI-FSM dataset size, including average number of SM and speckle datapoints per ROI

Dataset	No. of cells	No of repeats	SM tracklets per ROI (average)			Speckle detection per frame per ROI (average)		
			Whole	Center	Periphery	Whole	Center	Periphery
CD36^WT^	25	8	2,340	1,629	708	696	462	205
CD36^G12V^	27	7	2,386	1,890	495	720	489	216

### Statistical testing for SMI-FSM

All statistical tests were performed as previously described by Dasgupta et al. (2023).

In brief, to compare SM or speckle properties, or MVRG coefficients, between CD36^WT^ and CD36^G12V^, a two-sample *t* test was performed. To compare SM or speckle properties, or MVRG coefficients, between the free and confined subsets of SM tracklets in a cell, or between the center and periphery regions of a cell, a paired-sample *t* test was performed. Data normality was assessed using the MATLAB function lillietest(), which uses the Lilliefors test to determine whether the sample data are consistent with a normal distribution. For all tests, comparisons with P values ≤ 0.05, 0.01, or 0.001 were indicated in the figures using *, **, or ***, respectively, while comparisons with a P value > 0.05 were not marked (to reduce clutter), unless noted otherwise in figure legends.

To determine whether there was a significant relationship between an SM tracklet property and an associated CA speckle property, a one-sample *t* test was performed, with the null hypothesis that the single-cell MVRG coefficients followed a normal distribution with mean zero. A relationship was considered significant if the *t* test yielded a P value ≤ 0.05. To assist with visualizing these relationships, the MVRG coefficient plots were annotated with overhead triangle pictograms, where the triangle slopes represent the significance and sign of the MVRG coefficients. Nonsignificant MVRG coefficients were represented with rectangles.

### Live cell simultaneous SiR-actin and SMI data acquisition and analysis

Live TIME cells expressing HaloTag-CD36^WT^ or HaloTag-CD36^G12V^ were labeled with 3 nM JF549-Halo ligand as described in the “Halo labeling of CD36 for live-cell SMI-FSM” section. During the incubation in dye-free medium, to label actin, samples were incubated with a combination of 100 nM SiR-Actin and 10 μM Verapamil (SiR-Actin Kit, CY-SC001, Cytoskeleton) in complete media for an additional 1 h 45 min, as per the manufacturer’s instructions. Samples were then prepared for imaging as described in the “Live-cell SMI-FSM data acquisition” section and imaged at 37°C using the Olympus IX83 TIRF microscope described earlier. As with SMI-FSM imaging, the videos were acquired with MetaMorph in the stream acquisition mode, with the two channels acquired simultaneously, using the trigger function to control illumination. The 561 channel was used for acquiring SMI videos, and the 640 channel was used to acquire SiR-labelled actin videos. The 561 channel was acquired at 10 Hz for 30 s (301 frames). Similar to the 491 channel in the SMI-FSM section, the 640 channel was also acquired at 10 Hz, but triggered to illuminate the sample only every 50^th^ frame (for 99 ms each), resulting in a time-lapse at 0.2 Hz for 7 SiR-Actin frames. Laser powers at the sample position (in the widefield illumination configuration) were 8.8 and 4 mW for the 561 and 640 nm laser lines, respectively. Every movie was preceded by a brightfield snapshot of the imaged cell region to visually check cell viability and to aid with manual delineation (if needed) of the ROI mask for the ensuing analysis.

SM tracks were constructed from the SMI streams and their diffusion classified (into confined, free, immobile, and directed) as described in the “Live-cell SMI-FSM analysis” section. The constructed tracks were overlaid on a maximum intensity projection of the actin frames. To obtain the actin intensity at confined tracks, the actin intensity was read at every point of a confined track, and then the average actin intensity at confined tracks was calculated. This was compared with the whole-cell actin intensity using a paired, right-tailed Wilcoxon signed-rank test. In addition, the mean effect size (comparing intensity at confined tracks to whole cell intensity) was calculated using Cohen’s *d* measurement with 3,000 bootstraps to predict the 95% confidence interval.

### PLA: Experiments and analysis

#### Whole cell PLA

TIME cells grown on 18-mm-type 1.5 fibronectin-coated coverslips (Neuvitro Corporation, ref GG-18-15-Fibronectin) were transiently transfected with Halo-CD36^WT^ or Halo-CD36^G12V^, fixed the next day with 4% PFA (Electron Microscopy Sciences) at RT, and then processed for PLA following the manufacturer’s instructions (Millipore Sigma). Briefly, cells were first permeabilized with PBS containing 3% BSA +0.1% Triton X-100 for 30 min and then blocked with Duolink blocking solution for 45 min at 37°C. Primary antibodies diluted in the Duolink blocking solution were added to each coverslip for 1 h at RT. Antibody pairs used for PLA are listed in Table 6. Coverslips were washed twice for 5 min with Wash Buffer A at RT. PLA donkey anti-rabbit PLUS and donkey anti-mouse MINUS probes were diluted 1:5 in Duolink blocking solution, and 40 μl was added to coverslips for 1 h at 37°C. Next, cells were washed twice with buffer A for 5 min each at RT. For probe ligation, Ligation Stock Buffer was diluted 1:5 in double-distilled water, and Ligase from the PLA kit was added at a 1:40 dilution in ligase buffer. 40 μl of the diluted ligase solution was added to each coverslip and incubated at 37°C for 30 min. Following this incubation, coverslips were washed twice with Wash Buffer A for 5 min at RT. For amplification, Amplification Stock Buffer was diluted 1:5 in double-distilled water, and Polymerase from the PLA kit was added 1:80 to the diluted amplification solution. 40 μl of the diluted amplification buffer was added to each coverslip and incubated at 37°C for 100 min. Coverslips were washed twice with Wash Buffer B (100 mM NaCl, 35 mM Tris-base, and Tris-HCl 215 mM, pH 7.4) for 10 min at RT. For identification of the transfected cells, a secondary donkey anti-rabbit coupled to Alexa Fluor 488 was added in blocking buffer for 30 min at RT. Coverslips were quickly rinsed twice with Wash Buffer B and then postfixed using 4% PFA at RT for 10 min. Finally, cells were washed with 1:100 Wash Buffer B for 1 min and mounted on slides with Prolong Glass Antifade mounting media (Thermo Fisher Scientific).

**Table 6.** Antibodies used for whole cell PLA experiments

Primary antibodies	Target	Species	Concentration	Provider	Ref
Anti-CD36 (Clone131.2)	CD36 extracellular domain	Mouse	1:1,000	Gift from Dr. Tandon	N/A
Anti-Integrin β-1 antibody [12G10]	Active ITGB1	Mouse	1:1,000	Abcam	ab30394
Anti-Integrin β-1 antibody [K20]	Total ITGB1	Mouse	1:1,000	Santa Cruz	sc-18887
Anti-mitofilin antibody [2E4AD5]	Mitofilin	Mouse	1:1,000	Abcam	ab110329
Anti-HT antibody	HaloTag	Rabbit	1:1,000	Promega	G9281
Anti-GFP antibody	GFP	Rabbit	1:1,000	Abcam	ab290

Image acquisition was performed on a spinning-disk confocal microscope WaveFx1 (Yokogawa CSU-10 on an Evident IX81 base installed by Quorum Technologies) in the Cell Imaging Centre of the Faculty of Medicine and Dentistry, University of Alberta, Edmonton. Fluorescent dyes were excited using a laser merge module from Spectral Applied Physics (Richmond Hill) equipped with 491, 561, and 643 nm solid-state lasers. Image acquisitions were performed using the appropriate emission filters (Chroma) mounted on a filter wheel (Sutter Instrument) through an oil-immersion 60× 1.42 numerical aperture objective with an EMCCD ImageEM camera (Hamamatsu Photonics) using Volocity software (Quorum Technologies).

Cells expressing Halo-CD36^WT^ or Halo-CD36^G12V^ were identified using the Alexa Fluor 488 signal and segmented. The PLA dots in the 561 nm channel were segmented using Otsu’s thresholding method. The level of PLA-positive reaction was then calculated as the area of all the PLA dots segmented within a cell divided by the cell area, giving the percent of cell area occupied by PLA dots. A rank-based nonparametric ANOVA Kruskal–Wallis test was used to assess if any median in the group was different from the rest. This was followed by a pairwise two-sided Wilcoxon rank sum test to compare pairs within the group. The resultant P value was evaluated for significance by comparison with the significance threshold calculated using the Dunn–Sidak correction for a total type I error of 0.05.

#### Cell surface PLA

TIME cells transiently transfected with Halo-CD36^WT^ or Halo-CD36^G12V^ were labelled with Halo ligand conjugated to JF549 (for CD36 detection) and then fixed using 4% PFA as described in the “Sample preparation and labelling for fixed-cell imaging for colocalization analysis” section. PLA was then carried out using Duolink In Situ Detection Reagents (DUO92013; Millipore Sigma), together with phalloidin staining, as follows. Postfixation, samples were washed with PBS and incubated in Duolink blocking solution for 60 min at 37°C in a humidified chamber. Samples were incubated with primary antibodies against CD36 (FA6-152, ab17044; Abcam) at 1:400 dilution or against β_1_-integrin (K20, Beckman Coulter, IMBULK1B) at 1:10,000 dilution in Duolink Antibody Diluent for 1 h at RT. This was followed by two 5-min washes in Wash Buffer A (DUO82049). Samples were then permeabilized using 0.1% Triton X-100 (1 min, RT), followed by 3 washes (1 min each), followed by another round of blocking in Duolink blocking solution for 60 min at 37°C in a humidified chamber. Next, all samples were incubated with Anti-HaloTag pAb (G9281; Promega) at 1:500 dilution in Duolink Antibody Diluent for 1 h at RT. Samples were next incubated with Duolink PLA Probes (donkey anti-mouse [against CD36 or β_1_-integrin] and donkey anti-rabbit [against HaloTag]) along with Alexa Fluor 488–conjugated Phalloidin for actin staining at 1:500 dilution (A12379; Thermo Fisher Scientific) for 60 min at 37°C in a humidified chamber. Ligation and signal amplification steps were carried out as described in the whole cell PLA section. Finally, samples were incubated in imaging buffer (OxyFluor 1%, glucose 0.45%, and Trolox 2 mM) to reduce photobleaching before and during imaging. Within each sample (dish), images were acquired of both cells expressing CD36 or not expressing CD36, as identified by HaloTag labeling of Halo-CD36^WT^ or Halo-CD36^G12V^. Image acquisition was performed using the IX83 Olympus TIRF microscope described in the Fixed-cell imaging for colocalization analysis section, using laser powers of 1.2, 8.8 and 2.4 mW at the sample for the 491, 561, and 640 channels, respectively.

CD36 (HaloTag-JF549) and PLA (640 channel) signals were detected in both CD36-expressing and non-expressing cells, as described in the section Punctate object detection in fixed cells and TetraSpeck beads, but with an α-value of 0.001 for PLA signal detection. The phalloidin signal was used to manually identify the cell mask. PLA signal quantification was then calculated as the ratio of PLA detection density within the cell mask in CD36-expressing cells to the average PLA detection density within the cell mask measured in CD36–non-expressing cells from the same dish. A two-sample *t* test was performed to compare the PLA signals of CD36^WT^–β_1_-integrin interactions to those of CD36^G12V^–β_1_-integrin interactions.

### Simulation of free diffusion and diffusion in confinement areas of different sizes

Three sets of 4,305,000 SM tracks each were simulated. Each set consisted of 4,100 different diffusion parameters (diffusion coefficient and confinement radius, the latter only in the confined diffusion case) × 21 lifetimes (20–40 frames) × 50 repeats. One set (the control) consisted of free diffusion, while two sets consisted of confined diffusion. All simulations utilized the diffusion coefficient distribution of free CD36^WT^ tracklets. The two confined diffusion datasets utilized the confinement radius distribution of confined CD36^WT^ tracklets for one and that of confined CD36^G12V^ tracklets for the other.

2D Brownian motion was simulated as a 2D random walk, with each step in the x- and y-directions drawn from a normal distribution with mean 0 and standard deviation equal to $\sqrt{2D∆t}$. D = the diffusion coefficient of a particular simulation. Δt = the simulation timestep = 0.1 s (equivalent to experimental data). For confined diffusion, the particle was simulated to undergo this random walk inside a circular area, with radius R = confinement radius of a particular simulation. The particle was retained inside the circular area by bouncing it off the circle boundary if a step would take it across the boundary.

### Online supplemental material

Fig. S1 shows the expression level of CD36 and the density distribution of the various imaged molecules and intensity of actin in TIME cells. Fig. S2 shows a detailed example of conditional colocalization analysis (CD36 colocalization with β_1_-integrin conditional on CD9). Fig. S3 shows CD36 colocalization with activated β_1_-integrin and actin enrichment analysis for CD36 molecules colocalized or not colocalized with β_1_-integrin or CD9. Fig. S4 shows sequence confirmation and surface expression of different CD36 mutants. Fig. S5 shows the interactions of CD36 with β_1_-integrin and their disruption by the G12V mutation using PLA. Fig. S6 shows regression coefficients from MVRG analysis on local CA speckle displacement and density, and from MVRG analysis on local CA speckle displacement alone. Fig. S7 shows the comparison of actin intensity at confined CD36 tracks vs. whole cell actin intensity. Fig. S8 shows Cohen’s *d* values for the experiments presented in Fig. 5. Table S1 shows the following measurements underlying the colocalization networks shown in Fig. 1: colocalization and conditional colocalization measures, -log_10_(P values) evaluating the significance of the various colocalization and conditional colocalization measures, and mean effect size quantifying the magnitude of the observed colocalization and conditional colocalization relationships. Video 1 shows two SMI movies, one of CD36^WT^ and one of CD36^G12V^. Video 2 shows two FSM movies of actin, corresponding to the SMI movies shown in Video 1. Video 3 shows SM particle tracking. Video 4 shows FSM speckle detection and tracking.

## Supplementary Material

Review History

Table S1 shows the measurements underlying the colocalization networks shown in Fig. 1.

## Data Availability

The data generated for this work are available from the corresponding authors upon reasonable request. The software used for data analysis is previously published and publicly available on GitHub: Colocalization analysis: https://github.com/kjaqaman/conditionalColoc. SMI-FSM analysis: https://github.com/kjaqaman/SMI-FSM. Particle tracking: https://github.com/DanuserLab/u-track. Quantitative fluorescent speckle microscopy: https://github.com/DanuserLab/QFSM. Segmentation: https://github.com/DanuserLab/u-segment.

## References

[bib1] Aguet, F., C.N. Antonescu, M. Mettlen, S.L. Schmid, and G. Danuser. 2013. Advances in analysis of low signal-to-noise images link dynamin and AP2 to the functions of an endocytic checkpoint. Dev. Cell. 26:279–291. 10.1016/j.devcel.2013.06.019 23891661 PMC3939604

[bib2] Alam, M.S. 2018. Proximity ligation assay (PLA). Curr. Protoc. Immunol. 123:e58. 10.1002/cpim.58 30238640 PMC6205916

[bib3] Bailey, R.L., J.M. Herbert, K. Khan, V.L. Heath, R. Bicknell, and M.G. Tomlinson. 2011. The emerging role of tetraspanin microdomains on endothelial cells. Biochem. Soc. Trans. 39:1667–1673. 10.1042/BST20110745 22103505

[bib4] Bamberger, M.E., M.E. Harris, D.R. McDonald, J. Husemann, and G.E. Landreth. 2003. A cell surface receptor complex for fibrillar beta-amyloid mediates microglial activation. J. Neurosci. 23:2665–2674. 10.1523/JNEUROSCI.23-07-02665.2003 12684452 PMC6742111

[bib5] Berditchevski, F. 2001. Complexes of tetraspanins with integrins: More than meets the eye. J. Cell Sci. 114:4143–4151. 10.1242/jcs.114.23.4143 11739647

[bib6] Berditchevski, F., E. Odintsova, S. Sawada, and E. Gilbert. 2002. Expression of the palmitoylation-deficient CD151 weakens the association of alpha 3 beta 1 integrin with the tetraspanin-enriched microdomains and affects integrin-dependent signaling. J. Biol. Chem. 277:36991–37000. 10.1074/jbc.M205265200 12110679

[bib7] Brakebusch, C., and R. Fassler. 2003. The integrin-actin connection, an eternal love affair. EMBO J. 22:2324–2333. 10.1093/emboj/cdg245 12743027 PMC156003

[bib8] Byron, A., J.D. Humphries, J.A. Askari, S.E. Craig, A.P. Mould, and M.J. Humphries. 2009. Anti-integrin monoclonal antibodies. J. Cell Sci. 122:4009–4011. 10.1242/jcs.056770 19910492 PMC3329622

[bib9] Charrin, S., S. Jouannet, C. Boucheix, and E. Rubinstein. 2014. Tetraspanins at a glance. J. Cell Sci. 127:3641–3648. 10.1242/jcs.154906 25128561

[bib10] Charrin, S., F. le Naour, O. Silvie, P.E. Milhiet, C. Boucheix, and E. Rubinstein. 2009. Lateral organization of membrane proteins: Tetraspanins spin their web. Biochem. J. 420:133–154. 10.1042/BJ20082422 19426143

[bib11] Charrin, S., S. Manié, C. Thiele, M. Billard, D. Gerlier, C. Boucheix, and E. Rubinstein. 2003. A physical and functional link between cholesterol and tetraspanins. Eur. J. Immunol. 33:2479–2489. 10.1002/eji.200323884 12938224

[bib12] Chen, H., M.E. Herndon, and J. Lawler. 2000. The cell biology of thrombospondin-1. Matrix Biol. 19:597–614. 10.1016/s0945-053x(00)00107-4 11102749

[bib13] Chen, Y., J. Zhang, W. Cui, and R.L. Silverstein. 2022. CD36, a signaling receptor and fatty acid transporter that regulates immune cell metabolism and fate. J. Exp. Med. 219:e20211314. 10.1084/jem.20211314 35438721 PMC9022290

[bib14] Chu, L.Y., D.P. Ramakrishnan, and R.L. Silverstein. 2013. Thrombospondin-1 modulates VEGF signaling via CD36 by recruiting SHP-1 to VEGFR2 complex in microvascular endothelial cells. Blood. 122:1822–1832. 10.1182/blood-2013-01-482315 23896411 PMC3765061

[bib15] Coffey, G.P., R. Rajapaksa, R. Liu, O. Sharpe, C.C. Kuo, S.W. Krauss, Y. Sagi, R.E. Davis, L.M. Staudt, J.P. Sharman, . 2009. Engagement of CD81 induces ezrin tyrosine phosphorylation and its cellular redistribution with filamentous actin. J. Cell Sci. 122:3137–3144. 10.1242/jcs.045658 19654214 PMC2729262

[bib16] da Rocha-Azevedo, B., S. Lee, A. Dasgupta, A.R. Vega, L.R. de Oliveira, T. Kim, M. Kittisopikul, Z.A. Malik, and K. Jaqaman. 2020. Heterogeneity in VEGF Receptor-2 mobility and organization on the endothelial cell surface leads to diverse models of activation by VEGF. Cell Rep. 32:108187. 10.1016/j.celrep.2020.108187 32997988 PMC7541195

[bib17] Danuser, G., and C.M. Waterman-Storer. 2006. Quantitative fluorescent speckle microscopy of cytoskeleton dynamics. Annu. Rev. Biophys. Biomol. Struct. 35:361–387. 10.1146/annurev.biophys.35.040405.102114 16689641

[bib18] Dasgupta, A., H.T. Ngo, D. Tschoerner, N. Touret, B. da Rocha-Azevedo, and K. Jaqaman. 2023. Multiscale imaging and quantitative analysis of plasma membrane protein-cortical actin interplay. Biophys. J. 122:3798–3815. 10.1016/j.bpj.2023.08.007 37571825 PMC10541498

[bib19] Dawson, D.W., S.F. Pearce, R.Q. Zhong, R.L. Silverstein, W.A. Frazier, and N.P. Bouck. 1997. CD36 mediates the in vitro inhibitory effects of thrombospondin-1 on endothelial cells. J. Cell Biol. 138:707–717. 10.1083/jcb.138.3.707 9245797 PMC2141641

[bib20] Delorme, V., M. Machacek, C. DerMardirossian, K.L. Anderson, T. Wittmann, D. Hanein, C. Waterman-Storer, G. Danuser, and G.M. Bokoch. 2007. Cofilin activity downstream of Pak1 regulates cell protrusion efficiency by organizing lamellipodium and lamella actin networks. Dev. Cell. 13:646–662. 10.1016/j.devcel.2007.08.011 17981134 PMC2170459

[bib21] Doliwa, B., and A. Heuer. 1998. Cage effect, local anisotropies, and dynamic heterogeneities at the glass transition: A computer study of hard spheres. Phys. Rev. Lett. 80:4915–4918. 10.1103/physrevlett.80.4915

[bib22] Echarri, A., and M.A. Del Pozo. 2015. Caveolae – mechanosensitive membrane invaginations linked to actin filaments. J. Cell Sci. 128:2747–2758. 10.1242/jcs.153940 26159735

[bib23] Ehringer, W.D., S. Yamany, K. Steier, A. Farag, F.J. Roisen, A. Dozier, and F.N. Miller. 1999. Quantitative image analysis of F-actin in endothelial cells. Microcirculation. 6:291–303. 10654280

[bib24] Febbraio, M., D.P. Hajjar, and R.L. Silverstein. 2001. CD36: a class B scavenger receptor involved in angiogenesis, atherosclerosis, inflammation, and lipid metabolism. J. Clin. Invest. 108:785–791. 10.1172/JCI14006 11560944 PMC200943

[bib25] Ferrari, R., A.J. Manfroi, and W.R. Young. 2001. Strongly and weakly self-similar diffusion. Physica D. 154:111–137. 10.1016/s0167-2789(01)00234-2

[bib26] Frank, P.G., S.E. Woodman, D.S. Park, and M.P. Lisanti. 2003. Caveolin, caveolae, and endothelial cell function. Arterioscler Thromb. Vasc. Biol. 23:1161–1168. 10.1161/01.ATV.0000070546.16946.3A 12689915

[bib27] Freeman, S.A., A. Vega, M. Riedl, R.F. Collins, P.P. Ostrowski, E.C. Woods, C.R. Bertozzi, M.I. Tammi, D.S. Lidke, P. Johnson, . 2018. Transmembrane pickets connect cyto- and pericellular skeletons forming barriers to receptor engagement. Cell. 172:305–317.e10. 10.1016/j.cell.2017.12.023 29328918 PMC5929997

[bib28] Garcia-Parajo, M.F., A. Cambi, J.A. Torreno-Pina, N. Thompson, and K. Jacobson. 2014. Nanoclustering as a dominant feature of plasma membrane organization. J. Cell Sci. 127:4995–5005. 10.1242/jcs.146340 25453114 PMC4260763

[bib29] Garcia-Parajo, M.F., and S. Mayor. 2024. The ubiquitous nanocluster: A molecular scale organizing principle that governs cellular information flow. Curr. Opin. Cell Biol. 86:102285. 10.1016/j.ceb.2023.102285 38056142 PMC7617173

[bib30] Geiger, B., A. Bershadsky, R. Pankov, and K.M. Yamada. 2001. Transmembrane extracellular matrix-cytoskeleton crosstalk. Nat. Rev. Mol. Cell Biol. 2:793–805. 10.1038/35099066 11715046

[bib31] Gerbod-Giannone, M.C., L. Dallet, G. Naudin, A. Sahin, M. Decossas, S. Poussard, and O. Lambert. 2019. Involvement of caveolin-1 and CD36 in native LDL endocytosis by endothelial cells. Biochim. Biophys. Acta Gen. Subj. 1863:830–838. 10.1016/j.bbagen.2019.01.005 30768959

[bib32] Gerhardt, H., M. Golding, M. Fruttiger, C. Ruhrberg, A. Lundkvist, A. Abramsson, M. Jeltsch, C. Mitchell, K. Alitalo, D. Shima, and C. Betsholtz. 2003. VEGF guides angiogenic sprouting utilizing endothelial tip cell filopodia. J. Cell Biol. 161:1163–1177. 10.1083/jcb.200302047 12810700 PMC2172999

[bib33] Githaka, J.M., A.R. Vega, M.A. Baird, M.W. Davidson, K. Jaqaman, and N. Touret. 2016. Ligand-induced growth and compaction of CD36 nanoclusters enriched in Fyn induces Fyn signaling. J. Cell Sci. 129:4175–4189. 10.1242/jcs.188946 27694211

[bib34] Goswami, D., K. Gowrishankar, S. Bilgrami, S. Ghosh, R. Raghupathy, R. Chadda, R. Vishwakarma, M. Rao, and S. Mayor. 2008. Nanoclusters of GPI-anchored proteins are formed by cortical actin-driven activity. Cell. 135:1085–1097. 10.1016/j.cell.2008.11.032 19070578 PMC7616455

[bib35] Gowrishankar, K., S. Ghosh, S. Saha, R. C, S. Mayor, and M. Rao. 2012. Active remodeling of cortical actin regulates spatiotemporal organization of cell surface molecules. Cell. 149:1353–1367. 10.1016/j.cell.2012.05.008 22682254

[bib36] Goyette, J., D.J. Nieves, Y. Ma, and K. Gaus. 2019. How does T cell receptor clustering impact on signal transduction? J. Cell Sci. 132:jcs226423. 10.1242/jcs.226423 30745330

[bib37] Gutiérrez-Martínez, E., S. Benet Garrabé, N. Mateos, I. Erkizia, J.A. Nieto-Garai, M. Lorizate, K.J.E. Borgman, C. Manzo, F. Campelo, N. Izquierdo-Useros, . 2023. Actin-regulated Siglec-1 nanoclustering influences HIV-1 capture and virus-containing compartment formation in dendritic cells. Elife. 12:e78836. 10.7554/eLife.78836 36940134 PMC10065798

[bib38] Hao, J.W., J. Wang, H. Guo, Y.Y. Zhao, H.H. Sun, Y.F. Li, X.Y. Lai, N. Zhao, X. Wang, C. Xie, . 2020. CD36 facilitates fatty acid uptake by dynamic palmitoylation-regulated endocytosis. Nat. Commun. 11:4765. 10.1038/s41467-020-18565-8 32958780 PMC7505845

[bib39] Heit, B., H. Kim, G. Cosío, D. Castano, R. Collins, C.A. Lowell, K.C. Kain, W.S. Trimble, and S. Grinstein. 2013. Multimolecular signaling complexes enable Syk-mediated signaling of CD36 internalization. Dev. Cell. 24:372–383. 10.1016/j.devcel.2013.01.007 23395392 PMC3586299

[bib40] Helmuth, J.A., G. Paul, and I.F. Sbalzarini. 2010. Beyond co-localization: Inferring spatial interactions between sub-cellular structures from microscopy images. Bmc Bioinformatics. 11:372. 10.1186/1471-2105-11-372 20609242 PMC2919515

[bib41] Hemler, M.E. 2005. Tetraspanin functions and associated microdomains. Nat. Rev. Mol. Cell Biol. 6:801–811. 10.1038/nrm1736 16314869

[bib42] Hsieh, M.-y., S. Yang, M.A. Raymond-Stinz, J.S. Edwards, and B.S. Wilson. 2010. Spatio-temporal modeling of signaling protein recruitment to EGFR. BMC Syst. Biol. 4:57. 10.1186/1752-0509-4-57 20459599 PMC2877007

[bib43] Huang, W., M. Febbraio, and R.L. Silverstein. 2011. CD9 tetraspanin interacts with CD36 on the surface of macrophages: A possible regulatory influence on uptake of oxidized low density lipoprotein. PLoS One. 6:e29092. 10.1371/journal.pone.0029092 22216174 PMC3244426

[bib44] Huang, W., R. Li, J. Zhang, Y. Cheng, D.P. Ramakrishnan, and R.L. Silverstein. 2023. A CD36 transmembrane domain peptide interrupts CD36 interactions with membrane partners on macrophages and inhibits atherogenic functions. Transl Res. 254:68–76. 10.1016/j.trsl.2022.10.005 36377115 PMC10863465

[bib45] Humphries, J.D., A. Byron, and M.J. Humphries. 2006. Integrin ligands at a glance. J. Cell Sci. 119:3901–3903. 10.1242/jcs.03098 16988024 PMC3380273

[bib46] Ivaska, J., and J. Heino. 2011. Cooperation between integrins and growth factor receptors in signaling and endocytosis. Annu. Rev. Cell Dev. Biol. 27:291–320. 10.1146/annurev-cellbio-092910-154017 21663443

[bib47] Jaqaman, K., J.A. Galbraith, M.W. Davidson, and C.G. Galbraith. 2016. Changes in single-molecule integrin dynamics linked to local cellular behavior. Mol. Biol. Cell. 27:1561–1569. 10.1091/mbc.E16-01-0018 27009207 PMC4865314

[bib48] Jaqaman, K., and S. Grinstein. 2012. Regulation from within: The cytoskeleton in transmembrane signaling. Trends Cell Biol. 22:515–526. 10.1016/j.tcb.2012.07.006 22917551 PMC3754899

[bib49] Jaqaman, K., H. Kuwata, N. Touret, R. Collins, W.S. Trimble, G. Danuser, and S. Grinstein. 2011. Cytoskeletal control of CD36 diffusion promotes its receptor and signaling function. Cell. 146:593–606. 10.1016/j.cell.2011.06.049 21854984 PMC3160624

[bib50] Jaqaman, K., D. Loerke, M. Mettlen, H. Kuwata, S. Grinstein, S.L. Schmid, and G. Danuser. 2008. Robust single-particle tracking in live-cell time-lapse sequences. Nat. Methods. 5:695–702. 10.1038/nmeth.1237 18641657 PMC2747604

[bib51] Jiménez, B., O.V. Volpert, S.E. Crawford, M. Febbraio, R.L. Silverstein, and N. Bouck. 2000. Signals leading to apoptosis-dependent inhibition of neovascularization by thrombospondin-1. Nat. Med. 6:41–48. 10.1038/71517 10613822

[bib52] Katasho, R., T. Nagano, T. Iwasaki, and S. Kamada. 2023. Nectin-4 regulates cellular senescence-associated enlargement of cell size. Sci. Rep. 13:21602. 10.1038/s41598-023-48890-z 38062106 PMC10703872

[bib53] Kazerounian, S., M. Duquette, M.A. Reyes, J.T. Lawler, K. Song, C. Perruzzi, L. Primo, R. Khosravi-Far, F. Bussolino, I. Rabinovitz, and J. Lawler. 2011. Priming of the vascular endothelial growth factor signaling pathway by thrombospondin-1, CD36, and spleen tyrosine kinase. Blood. 117:4658–4666. 10.1182/blood-2010-09-305284 21378271 PMC3099580

[bib54] Kim, C., F. Ye, and M.H. Ginsberg. 2011. Regulation of integrin activation. Annu. Rev. Cell Dev. Biol. 27:321–345. 10.1146/annurev-cellbio-100109-104104 21663444

[bib55] Kostic, A., and M.P. Sheetz. 2006. Fibronectin rigidity response through Fyn and p130Cas recruitment to the leading edge. Mol. Biol. Cell. 17:2684–2695. 10.1091/mbc.e05-12-1161 16597701 PMC1474803

[bib56] Kusumi, A., T.K. Fujiwara, R. Chadda, M. Xie, T.A. Tsunoyama, Z. Kalay, R.S. Kasai, and K.G. Suzuki. 2012. Dynamic organizing principles of the plasma membrane that regulate signal transduction: Commemorating the fortieth anniversary of singer and Nicolson’s fluid-mosaic model. Annu. Rev. Cell Dev Biol. 28:215–250. 10.1146/annurev-cellbio-100809-151736 22905956

[bib57] Lee, K., H.L. Elliott, Y. Oak, C.T. Zee, A. Groisman, J.D. Tytell, and G. Danuser. 2015. Functional hierarchy of redundant actin assembly factors revealed by fine-grained registration of intrinsic image fluctuations. Cell Syst. 1:37–50. 10.1016/j.cels.2015.07.001 26273703 PMC4530526

[bib58] Lehtimaki, J.I., E.K. Rajakyla, S. Tojkander, and P. Lappalainen. 2021. Generation of stress fibers through myosin-driven reorganization of the actin cortex. Elife. 10:e60710. 10.7554/eLife.60710 33506761 PMC7877910

[bib59] Li, J.H., P. Santos-Otte, B. Au, J. Rentsch, S. Block, and H. Ewers. 2020. Directed manipulation of membrane proteins by fluorescent magnetic nanoparticles. Nat. Commun. 11:4259. 10.1038/s41467-020-18087-3 32848156 PMC7450064

[bib60] Luse, M.A., M.G. Jackson, Z.J. Juśkiewicz, and B.E. Isakson. 2023. Physiological functions of caveolae in endothelium. Curr. Opin. Physiol. 35:100701. 10.1016/j.cophys.2023.100701 37873030 PMC10588508

[bib61] Maher, J.M., J.C. Markey, and D. Ebert-May. 2013. The other half of the story: Effect size analysis in quantitative research. CBE Life Sci. Educ. 12:345–351. 10.1187/cbe.13-04-0082 24006382 PMC3763001

[bib62] Maldonado, H., and L. Leyton. 2023. CSK-mediated signalling by integrins in cancer. Front. Cell Dev. Biol. 11:1214787. 10.3389/fcell.2023.1214787 37519303 PMC10382208

[bib63] McGilvray, I.D., L. Serghides, A. Kapus, O.D. Rotstein, and K.C. Kain. 2000. Nonopsonic monocyte/macrophage phagocytosis of plasmodium falciparum-parasitized erythrocytes: A role for CD36 in malarial clearance. Blood. 96:3231–3240. 11050008

[bib64] Mendoza, M.C., S. Besson, and G. Danuser. 2012. Quantitative fluorescent speckle microscopy (QFSM) to measure actin dynamics. Curr. Protoc. Cytom:Chapter 2:Unit2.18. 10.1002/0471142956.cy0218s62 PMC3688286 23042526

[bib65] Mendoza, M.C., M. Vilela, J.E. Juarez, J. Blenis, and G. Danuser. 2015. ERK reinforces actin polymerization to power persistent edge protrusion during motility. Sci. Signal. 8:ra47. 10.1126/scisignal.aaa8859 25990957 PMC4830495

[bib66] Miao, W.M., E. Vasile, W.S. Lane, and J. Lawler. 2001. CD36 associates with CD9 and integrins on human blood platelets. Blood. 97:1689–1696. 10.1182/blood.v97.6.1689 11238109

[bib67] Morgan, J.L. 2025. Alternative to the statistical mass confusion of testing for “no effect”. J. Cell Biol. 224:e202403034. 10.1083/jcb.202403034 40699150 PMC12286597

[bib68] Mugler, A., A.G. Bailey, K. Takahashi, and P.R. ten Wolde. 2012. Membrane clustering and the role of rebinding in biochemical signaling. Biophys. J. 102:1069–1078. 10.1016/j.bpj.2012.02.005 22404929 PMC3296021

[bib69] Mukherjee, A., R. Singh, S. Udayan, S. Biswas, P.P. Reddy, S. Manmadhan, G. George, S. Kumar, R. Das, B.M. Rao, and A. Gulyani. 2020. A Fyn biosensor reveals pulsatile, spatially localized kinase activity and signaling crosstalk in live mammalian cells. Elife. 9:e50571. 10.7554/eLife.50571 32017701 PMC7000222

[bib70] Noh, J., T. Isogai, J. Chi, K. Bhatt, and G. Danuser. 2022. Granger-causal inference of the lamellipodial actin regulator hierarchy by live cell imaging without perturbation. Cell Syst. 13:471–487.e8. 10.1016/j.cels.2022.05.003 35675823 PMC9271366

[bib71] O’Shea, J.J., and P.J. Murray. 2008. Cytokine signaling modules in inflammatory responses. Immunity. 28:477–487. 10.1016/j.immuni.2008.03.002 18400190 PMC2782488

[bib72] Obergfell, A., K. Eto, A. Mocsai, C. Buensuceso, S.L. Moores, J.S. Brugge, C.A. Lowell, and S.J. Shattil. 2002. Coordinate interactions of Csk, Src, and Syk kinases with αIIbβ3 initiate integrin signaling to the cytoskeleton. J. Cell Biol. 157:265–275. 10.1083/jcb.200112113 11940607 PMC2199242

[bib73] Peche, V.S., T.A. Pietka, M. Jacome-Sosa, D. Samovski, H. Palacios, G. Chatterjee-Basu, A.C. Dudley, W. Beatty, G.A. Meyer, I.J. Goldberg, and N.A. Abumrad. 2023. Endothelial cell CD36 regulates membrane ceramide formation, exosome fatty acid transfer and circulating fatty acid levels. Nat. Commun. 14:4029. 10.1038/s41467-023-39752-3 37419919 PMC10329018

[bib74] Peckham, H., L. Giuffrida, R. Wood, D. Gonsalvez, A. Ferner, T.J. Kilpatrick, S.S. Murray, and J. Xiao. 2016. Fyn is an intermediate kinase that BDNF utilizes to promote oligodendrocyte myelination. Glia. 64:255–269. 10.1002/glia.22927 26449489

[bib75] Pepino, M.Y., O. Kuda, D. Samovski, and N.A. Abumrad. 2014. Structure-function of CD36 and importance of fatty acid signal transduction in fat metabolism. Annu. Rev. Nutr. 34:281–303. 10.1146/annurev-nutr-071812-161220 24850384 PMC4329921

[bib76] Ponti, A., M. Machacek, S.L. Gupton, C.M. Waterman-Storer, and G. Danuser. 2004. Two distinct actin networks drive the protrusion of migrating cells. Science. 305:1782–1786. 10.1126/science.1100533 15375270

[bib77] Ponti, A., P. Vallotton, W.C. Salmon, C.M. Waterman-Storer, and G. Danuser. 2003. Computational analysis of F-actin turnover in cortical actin meshworks using fluorescent speckle microscopy. Biophys. J. 84:3336–3352. 10.1016/S0006-3495(03)70058-7 12719263 PMC1302894

[bib78] Primo, L., C. Ferrandi, C. Roca, S. Marchio, L. di Blasio, M. Alessio, and F. Bussolino. 2005. Identification of CD36 molecular features required for its in vitro angiostatic activity. FASED J. 19:1713–1715. 10.1096/fj.05-3697fje 16037098

[bib79] Ritchie, K., X.Y. Shan, J. Kondo, K. Iwasawa, T. Fujiwara, and A. Kusumi. 2005. Detection of non-Brownian diffusion in the cell membrane in single molecule tracking. Biophys. J. 88:2266–2277. 10.1529/biophysj.104.054106 15613635 PMC1305276

[bib80] Saini, A., E.G. Cardona, H. Huang, S.M. Khaing, K. Klein, K. Jaqaman, O. Julien, and N. Touret. 2025. Investigation of CD36 interactome provides insights into multimolecular complexes necessary for anti-angiogenic signalling. bioRxiv. 10.1101/2025.07.15.664851 (Preprint posted July 16, 2025).

[bib81] Sala-Valdés, M., A. Ursa, S. Charrin, E. Rubinstein, M.E. Hemler, F. Sánchez-Madrid, and M. Yáñez-Mó. 2006. EWI-2 and EWI-F link the tetraspanin web to the actin cytoskeleton through their direct association with ezrin-radixin-moesin proteins. J. Biol. Chem. 281:19665–19675. 10.1074/jbc.m602116200 16690612

[bib82] Sandilands, E., V.G. Brunton, and M.C. Frame. 2007. The membrane targeting and spatial activation of Src, Yes and Fyn is influenced by palmitoylation and distinct RhoB/RhoD endosome requirements. J. Cell Sci. 120:2555–2564. 10.1242/jcs.003657 17623777

[bib83] Schaphorst, K.L., F.M. Pavalko, C.E. Patterson, and J.G. Garcia. 1997. Thrombin-mediated focal adhesion plaque reorganization in endothelium: Role of protein phosphorylation. Am. J. Respir. Cell Mol Biol. 17:443–455. 10.1165/ajrcmb.17.4.2502 9376119

[bib84] Schmidt, S.C., A. Massenberg, Y. Homsi, D. Sons, and T. Lang. 2024. Microscopic clusters feature the composition of biochemical tetraspanin-assemblies and constitute building-blocks of tetraspanin enriched domains. Sci. Rep. 14:2093. 10.1038/s41598-024-52615-1 38267610 PMC10808221

[bib85] Schneider, D., and D.M. Engelman. 2004. Involvement of transmembrane domain interactions in signal transduction by alpha/beta integrins. J. Biol. Chem. 279:9840–9846. 10.1074/jbc.M312749200 14681217

[bib86] Shigeta, M., N. Sanzen, M. Ozawa, J. Gu, H. Hasegawa, and K. Sekiguchi. 2003. CD151 regulates epithelial cell-cell adhesion through PKC- and Cdc42-dependent actin cytoskeletal reorganization. J. Cell Biol. 163:165–176. 10.1083/jcb.200301075 14557253 PMC2173453

[bib87] Silverstein, R.L. 2025. Just say no to CD36 expression: A regulatory pathway with implications in many chronic diseases. Arterioscler Thromb. Vasc. Biol. 45:1087–1089. 10.1161/ATVBAHA.125.322894 40469036 PMC12197822

[bib88] Silverstein, R.L., and M. Febbraio. 2009. CD36, a scavenger receptor involved in immunity, metabolism, angiogenesis, and behavior. Sci. Signal. 2:re3. 10.1126/scisignal.272re3 19471024 PMC2811062

[bib89] Svitkina, T.M. 2020. Actin cell cortex: Structure and molecular organization. Trends Cell Biol. 30:556–565. 10.1016/j.tcb.2020.03.005 32278656 PMC7566779

[bib90] Takeda, Y., A.R. Kazarov, C.E. Butterfield, B.D. Hopkins, L.E. Benjamin, A. Kaipainen, and M.E. Hemler. 2007. Deletion of tetraspanin Cd151 results in decreased pathologic angiogenesis in vivo and in vitro. Blood. 109:1524–1532. 10.1182/blood-2006-08-041970 17023588 PMC1794066

[bib91] Teese, M.G., and D. Langosch. 2015. Role of GxxxG motifs in transmembrane domain interactions. Biochemistry. 54:5125–5135. 10.1021/acs.biochem.5b00495 26244771

[bib92] Thomann, D., D.R. Rines, P.K. Sorger, and G. Danuser. 2002. Automatic fluorescent tag detection in 3D with super-resolution: Application to the analysis of chromosome movement. J. Microsc. 208:49–64. 10.1046/j.1365-2818.2002.01066.x 12366597

[bib93] Thorne, R.F., J.F. Marshall, D.R. Shafren, P.G. Gibson, I.R. Hart, and G.F. Burns. 2000. The integrins alpha3beta1 and alpha6beta1 physically and functionally associate with CD36 in human melanoma cells. Requirement for the extracellular domain of CD36. J. Biol. Chem. 275:35264–35275. 10.1074/jbc.M003969200 10956645

[bib94] Toribio, V., and M. Yáñez-Mó. 2022. Tetraspanins interweave EV secretion, endosomal network dynamics and cellular metabolism. Eur. J. Cell Biol. 101:151229. 10.1016/j.ejcb.2022.151229 35500468

[bib95] Treanor, B., D. Depoil, A. Gonzalez-Granja, P. Barral, M. Weber, O. Dushek, A. Bruckbauer, and F.D. Batista. 2010. The membrane skeleton controls diffusion dynamics and signaling through the B cell receptor. Immunity. 32:187–199. 10.1016/j.immuni.2009.12.005 20171124 PMC2984614

[bib96] Truong Quang, B.-A., and P.-F. Lenne. 2014. Membrane microdomains: From seeing to understanding. Front. Plant Sci. 5:18. 10.3389/fpls.2014.00018 24600455 PMC3927121

[bib97] Uittenbogaard, A., P.W. Shaul, I.S. Yuhanna, A. Blair, and E.J. Smart. 2000. High density lipoprotein prevents oxidized low density lipoprotein-induced inhibition of endothelial nitric-oxide synthase localization and activation in caveolae. J. Biol. Chem. 275:11278–11283. 10.1074/jbc.275.15.11278 10753938

[bib98] van Deventer, S., A.B. Arp, and A.B. van Spriel. 2021. Dynamic plasma membrane organization: A complex symphony. Trends Cell Biol. 31:119–129. 10.1016/j.tcb.2020.11.004 33248874

[bib99] Vega-Lugo, J., B. da Rocha-Azevedo, A. Dasgupta, and K. Jaqaman. 2022. Analysis of conditional colocalization relationships and hierarchies in three-color microscopy images. J. Cell Biol. 221:e202106129. 10.1083/jcb.202106129 35552363 PMC9111757

[bib100] Vega, A.R., S.A. Freeman, S. Grinstein, and K. Jaqaman. 2018. Multistep track segmentation and motion classification for transient mobility analysis. Biophys. J. 114:1018–1025. 10.1016/j.bpj.2018.01.012 29539390 PMC5883548

[bib101] Verkhovsky, A.B., O.Y. Chaga, S. Schaub, T.M. Svitkina, J.J. Meister, and G.G. Borisy. 2003. Orientational order of the lamellipodial actin network as demonstrated in living motile cells. Mol. Biol. Cell. 14:4667–4675. 10.1091/mbc.e02-10-0630 13679520 PMC266781

[bib102] Vicente-Manzanares, M., and F. Sánchez-Madrid. 2018. Targeting the integrin interactome in human disease. Curr. Opin. Cell Biol. 55:17–23. 10.1016/j.ceb.2018.05.010 30006051

[bib103] Vignaud, T., C. Copos, C. Leterrier, M. Toro-Nahuelpan, Q. Tseng, J. Mahamid, L. Blanchoin, A. Mogilner, M. Thery, and L. Kurzawa. 2021. Stress fibres are embedded in a contractile cortical network. Nat. Mater. 20:410–420. 10.1038/s41563-020-00825-z 33077951 PMC7610471

[bib104] Wilkinson, B., J. Koenigsknecht-Talboo, C. Grommes, C.Y.D. Lee, and G. Landreth. 2006. Fibrillar beta-amyloid-stimulated intracellular signaling cascades require Vav for induction of respiratory burst and phagocytosis in monocytes and microglia. J. Biol. Chem. 281:20842–20850. 10.1074/jbc.M600627200 16728400

[bib105] Wilson, B.S., J.M. Oliver, and D.S. Lidke. 2011. Spatio-temporal signaling in mast cells. Adv. Exp. Med. Biol. 716:91–106. 10.1007/978-1-4419-9533-9_6 21713653 PMC3285534

[bib106] Wong, H.S., V. Jaumouillé, S.A. Freeman, S.A. Doodnauth, D. Schlam, J. Canton, I.M. Mukovozov, A. Saric, S. Grinstein, and L.A. Robinson. 2016. Chemokine signaling enhances CD36 responsiveness toward oxidized low-density lipoproteins and accelerates foam cell formation. Cell Rep. 14:2859–2871. 10.1016/j.celrep.2016.02.071 26997267

[bib107] Yadunandanan Nair, N., V. Samuel, L. Ramesh, A. Marib, D.T. David, and A. Sundararaman. 2022. Actin cytoskeleton in angiogenesis. Biol. Open. 11:bio058899. 10.1242/bio.058899 36444960 PMC9729668

[bib108] Yauch, R.L., A.R. Kazarov, B. Desai, R.T. Lee, and M.E. Hemler. 2000. Direct extracellular contact between integrin alpha(3)beta(1) and TM4SF protein CD151. J. Biol. Chem. 275:9230–9238. 10.1074/jbc.275.13.9230 10734060

[bib109] Zhang, B., J. Zerubia, and J.-C. Olivo-Marin. 2007. Gaussian approximations of fluorescence microscope point-spread function models. Appl. Opt. 46:1819–1829. 10.1364/ao.46.001819 17356626

[bib110] Zhang, F., J. Kotha, L.K. Jennings, and X.A. Zhang. 2009. Tetraspanins and vascular functions. Cardiovasc. Res. 83:7–15. 10.1093/cvr/cvp080 19251723 PMC2695701

[bib111] Zhang, S., W.C. Huang, P. Li, H. Guo, S.B. Poh, S.W. Brady, Y. Xiong, L.M. Tseng, S.H. Li, Z. Ding, . 2011. Combating trastuzumab resistance by targeting SRC, a common node downstream of multiple resistance pathways. Nat. Med. 17:461–469. 10.1038/nm.2309 21399647 PMC3877934

[bib112] Zuidscherwoude, M., F. Göttfert, V.M.E. Dunlock, C.G. Figdor, G. van den Bogaart, and A.B. van Spriel. 2015. The tetraspanin web revisited by super-resolution microscopy. Sci. Rep. 5:12201. 10.1038/srep12201 26183063 PMC4505338

