# Supplementary material for: Integrins and tetraspanins mediate CD36-actin coupling and regulate CD36 organization and signaling
Source: J Cell Biol. 2026 Aug 21;225(10):e202504156. doi: 10.1083/jcb.202504156 (PMC13496012; doi:10.1083/jcb.202504156)
Supplement: Supplementary file 1 [file jcb_202504156_review_history.pdf]

JCB

Integrins and tetraspanins mediate CD36-actin coupling and regulate CD36 organization and signaling

Soma Jana, Huong-Tra Ngo, Han Huang, Jaime Guerrero, Jesus Vega-Lugo, Nicolas Touret, Aparajita Dasgupta, and Khuloud Jaqaman

Corresponding Author(s): Khuloud Jaqaman, The University of Texas Southwestern Medical Center and Aparajita Dasgupta, The University of Texas Southwestern Medical Center

Review Timeline:

Submission Date:	2025-04-25
Editorial Decision:	2025-06-06
Revision Received:	2026-04-16
Editorial Decision:	2026-05-21
Revision Received:	2026-06-08

Monitoring Editor: Satyajit Mayor

Scientific Editor: Gabriele Stephan

Transaction Report:

(Note: With the exception of the correction of typographical or spelling errors that could be a source of ambiguity, letters and reports are not edited. The original formatting of letters and referee reports may not be reflected in this compilation.)

DOI: https://doi.org/10.1083/jcb.202504156

June 6, 2025

Re: JCB manuscript #202504156

Khuloud Jaqaman
The University of Texas Southwestern Medical Center

Dear Dr. Jaqaman,

Thank you for submitting your manuscript entitled "Integrins and tetraspanins mediate CD36-actin coupling and regulate CD36 organization and signaling". The manuscript has been evaluated by expert reviewers, whose reports are appended below. Unfortunately, after an assessment of the reviewer feedback, our editorial decision is against publication in JCB.

You will see that all reviewers acknowledge that this is an interesting study; however, they all voice several concerns and indicate that some conclusions are not sufficiently supported by the data. All the reviewers think the manuscript requires more detailed clarifications on data analysis or methodologies regarding colocalization analysis, quantifications and density distribution as well as MVRG analysis. In particular, reviewer #1 suggests providing experimental evidence for the existence of tetraspanin complexes in nanodomains and recommends further elucidating the mechanism underlying the coupling to cortical actin. Both reviewers #1 and #2 suggest alternative approaches to demonstrate molecular interactions between b1-integrin-tetraspanin complexes and CD36. Reviewer #3 criticizes density analyses and recommends complementing the data from colocalization analyses with non-imaging approaches. I feel that the points raised by the reviewers are more substantial than can be addressed in a typical revision period. If you wish to expedite publication of the current data, it may be best to pursue publication at another journal.

Given interest in the topic, I would be open to resubmission to JCB of a significantly revised and extended manuscript that fully addresses the reviewers' concerns and is subject to further peer-review. If you would like to resubmit this work to JCB, please contact the journal office to discuss an appeal of this decision or you may submit an appeal directly through our manuscript submission system. Please note that priority and novelty would be reassessed at resubmission. We would be happy to discuss the reviewer comments further once you've had a chance to consider the points raised in this letter.

Regardless of how you choose to proceed, we hope that the comments below will prove constructive as your interesting study progresses. You can contact the journal office with any questions at cellbio@rockefeller.edu.

Thank you for thinking of JCB as an appropriate place to publish your work.

Sincerely,

Satyajit Mayor
Monitoring Editor
Journal of Cell Biology

Gabriele Stephan
Scientific Editor
Journal of Cell Biology

Reviewer #1:

In this manuscript, Jana et al apply several advanced imaging techniques in fixed and living cells combined with different data analysis algorithms to resolve interactions between CD36, b1-integrins and different tetraspanins. The hypothesis of the authors is that Interactions between CD36, b1 integrins and tetraspanins nanodomains mediate the link between CD36 and cortical actin. By taking advantage of a point mutation of CD36 which abolishes the interaction of CD36 with the tetraspanin CD9, the authors then investigate how the extent of this mutation (and thus interaction with CD9) perturbs the interaction with integrin b1 and cortical actin. There are several studies recently performed by some of the authors in this work and by other groups that have already revealed a role for cortical actin regulating the spatiotemporal organization of receptors located at the cell membrane, even for those with no direct interaction with actin. Thus, although the concept is not new, the work presented in this manuscript is interesting and it nicely adds to the emerging view that multi-molecular interactions are crucial to initiate signalling and contribute to receptor function. There are however a number of statements and conclusions not fully supported by the experimental data provided so far, requiring further experiments before the paper could be considered for publication. In addition, many more details should be provided regarding the data analysis methodology, in order to make this manuscript more

accessible to the biologist readership.

Major comments that require revision:
1) Page 4: the authors should carefully explain how the conditional colocalization analysis is performed, rather than simply refereeing to the paper by Vega-Lugo et al.
2) Density distribution and degree of co-localization: It is not clear whether the experiments shown in Figure 1, and supplementary figures S1 & S2 have been performed at saturating labelling densities or sub-labeling conditions to identify individual puncta in the diffraction limited TIRF images. In particular, the degree of colocalization is largely affected by the labeling densities of the molecules involved. How the expression levels of the different proteins involved and their labeling have been taken into account for the analysis? From the quantification of Fig. S1, there is higher expression levels of all the molecules investigated at the periphery and thus a "simple" colocalization analysis would render higher coincidence colocalization of these partners just based on densities, and not as a result of any active process going on.
3) Along these lines, I find Figures 1F,G and Figure 1M,N extremely difficult to understand and the figure caption does not help much. Although the plots shown in Figure S2 are intended to provide the quantification of CD36 colocalization with the different partners, the explanations provided in the caption and/or methods are quite confusing. For instance, the title of Figure S2A refers to CD36wt colocalization with b1 integrin, but the x-axis shows "overall", CD36 w/CD9 and b1-integrin w/CD9 ... what does this exactly means? What is overall?, what are nullTR, randC? How does the statistical significance has been calculated?. Same comments apply to the rest of the panels.
4) Page 5: the authors give for granted that these proteins exist in nanodomains without providing experimental evidence for this. This should be validated with additional experiments & analysis. In particular, labeling of tetraspanins without inducing artifacts is quite challenging... In addition what do the authors mean exactly by the term "cooperativity". Along these lines, there is also no evidence of b1-tetraspanins nanodomains? Are they complexes? or are these two separate nanodomains in close proximity to each other? Is this what the authors refer to as cooperativity?
5) The expression levels of the G12V are approximately two-fold lower than that of CD36, in particular at the cell periphery (Fig. S1). How does this impact the degree of co-localization between the different partners?
6) Page 7: More detailed explanations on the MVRG analysis should be provided.
7) Page 7, lines 187-189: I don´t agree with this statement. The mutation results shown in Fig. 2A,B indicate that the weakened coupling with CA is mediated by a reduced interaction with CD9, but it DO NOT provide evidence that the complexes contribute to the link between CD36 and CA. If I understand correctly Fig. 2A,B the differences between the WT and the mutant are more pronounced at low speckle density and in the cell center, while no differences between WT and mutant are observed at the cell periphery. Yet, the enrichment of integrins occurs preferentially at the cell periphery. Thus, whether integrins (nanodomains) are involved in this process is not granted by the results provided here, on the contrary.
8) Page 8, lines 195-200. The reasoning is likely to be true, but it would need to be shown in a more robust and quantitative manner. The involvement of b1 integrins, for instance, is not demonstrated at all here. One way to assess the role of integrins and tetraspanins in this process would be to perform dual color SPT experiments between b1 and CD36wt and between b1 and the mutant. Alternatively, high-density multi-color SPT experiments as recently implemented by Mateos et al, ACS Nano 2024 could directly assess real-time interactions between different partners at the single molecule level. The authors could follow a similar approach or at least discuss the advantage and limitations of their methodology as compared to these new approaches (including the work from Niederauer et al. Nat. Comm. 2023: Dual-color DNA-PAINT single-particle tracking enables extended studies of membrane protein interactions).
9) An important experiment to be performed here is identifying the role of integrins in this process (related to the discussion provided in page 13), as integrins could provide the link between the PM and the CA, only when active. Thus, comparison of the results obtained under conditions that keep b1 active and non-active would clarify the mechanism of coupling to CA.

Minor comments:

1) Page 3, lines 57-60: The paper by Gutierrez-Martinez eLife 2023 provides further proof of receptors lacking specific actin binding motifs and still their spatiotemporal organization is influenced by the cortical actin. It might be worth citing.
2) As the data provided are quite complex to interpret, it is imperative that the captions associated to the figures are explained in much better detail.

Reviewer #2:

In this manuscript, Jana et al. investigate the organization of CD36 at the plasma membrane, with a particular focus on its colocalization with integrins and tetraspanins, as well as its association with the cortical actin cytoskeleton. The study uses a previously published conditional colocalization analysis by the same group to examine the spatial distribution of these molecules, alongside speckle analysis to assess the dynamic behavior of CD36 in relation to the actin cytoskeleton. A mutant form of CD36 (G12V), predicted to exhibit reduced binding to CD9, is used to evaluate the effects of altered CD36-tetraspanin interactions on both its distribution and actin association. The authors propose a previously uncharacterized connection between these molecular components and present data on downstream signaling events affected by perturbation of this interaction.

Overall, the manuscript provides new observations on the organization of membrane-associated proteins. Yet, the main conclusions rely exclusively on two experimental approaches (colocalization and speckle analyses) that, by itself, do not provide sufficient evidence to fully support the claims of the authors. Also, several choices with respect to parameter selection and data analysis are made without adequate justification. The impact of alternative parameter selections is also not addressed. The manuscript is very technical and it is not always evident how the reported findings translate to broader molecular behavior in cells. Moreover, conclusions about molecular interactions are based on a single CD36 mutation that is not thoroughly characterized. It remains unclear to what extent this mutant retains interactions with other proteins, something that is also not clarified in the current manuscript. Lastly, the absence of functional data on cellular behaviour limits the advance in understanding of how altered CD36 behavior may influence cellular outcomes.

The following comments focus selectively on the colocalization analysis, but comparable concerns apply to the speckle analysis section. Also for the speckle analysis, several choices, such as the classification of high versus low speckle density, are not well substantiated and complicate data interpretation. Furthermore, the use of non-intuitive parameters (e.g., the MSS slope derived from MVRG analysis) introduces challenges for deriving clear biological meaning from the results.

Colocalization analysis (Figure 1/5):

The first section of the manuscript aims to study the "interactions of CD36 with integrins and tetraspanins in their cellular context". For this, the authors use a previously published conditional colocalization analysis from the same group to quantify the organization of these membrane proteins. Although sophisticated, there are many open questions related to this conditional colocalization analysis and related choices from the authors that complicate the interpretation of the conditional colocalizaton analysis.

First, the authors use a colocalization radius of 2 pixels (162 nm). The rationale for selecting this specific radius is not clearly stated, nor is it discussed how selecting a different radius would affect the outcome of the analysis. The manuscript states that the 2-pixel radius increases "the accuracy of the analysis," but the basis for this claim and the supporting data are not provided.

Second, the authors have chosen to separate their analysis between the center and the periphery of the cell (a band of ~4um from the edge). The two reasons that the authors give are "to avoid artifactual colocalization due to large-scale molecule co-distribution" and that "the periphery had substantively different actin cytoskeleton architecture than the center". Both arguments, however, raise questions about the strength of the analysis and the reason why 4um is chosen as a cutoff.

Specifically, the first argument suggests that the density of the investigated molecules could influence the colocalization values. Yet, the authors for example report stronger colocalization in the periphery as compared to the center, but if density affects colocalization, the interpretation of this difference is unclear. Also, the authors indicate that the distribution for CD36 changes with the G12V mutation but it is not discussed how this altered distribution could influence the colocalization values. It is therefore not obvious what higher colocalization (p-)values mean for the actual proximity of the two/three proteins. Also, since the CD36 is introduced by overexpression, it raises the question of how a specific expression level was chosen and how it was ensured that cells with similar expression values were picked. And how does the CD36 WT/CD36 G12V overexpression compare to native CD36 levels?

The second argument considers actin concentrations towards the periphery but this also lacks sufficient clarification. The image in Figure S1F does indeed show a higher actin intensity but not all of the observed actin is cortical actin. In the image, actin fibers and other actin related structures are observed at the periphery that would not necessarily affect CD36 diffusion or directly couple to CD36. Aside from these specific issues with the presented arguments, the reason for precisely selecting 4 μm as the boundary is not explained and whether it corresponds to a clear threshold in actin and/or molecular distribution is not addressed. Furthermore, and importantly, the presented colocalization alone does not allow the investigation of molecular interactions. At best, it indicates spatial proximity. To demonstrate molecular interactions more directly, complementary approaches such as dual-color single-particle tracking, fluorescence fluctuation spectroscopy, or biochemical methods like co-immunoprecipitation would be required.

Lastly, the statistics and presentation of the colocalization values require further clarification. While significant p-values suggest that the observed distributions differ from random expectations, it is unclear whether and how these p-values can be compared across conditions. For example, the authors state that the G12V mutation lowers the interaction of CD36 with CD9. Yet, both in the center and the periphery, CD36-CD9 interactions remain significant. It is therefore unclear how the authors reach the conclusion that the colocalization is less. The same is true for for example the Caveolin localization where the conclusion that the G12V mutant colocalizes more seems to be based on an p-value increase from 1.4 to 1.7. Furthermore, other remarkable findings, for example the increased colocalization between B1 integrin and CD81 in the CD36G12V conditions, are not discussed but again, raise questions about the approach since it is not immediately obvious why this would occur. Finally, the p-values for for example the CD36/B1 colocalization change quite considerably depending on the colabel (6.5, 2.9, 5.4), and it is unclear how that should be interpreted or what the origin of these differences are.

Reviewer #3:

Summary: This study investigates the role of the association of CD36 with tetraspanins and integrins on CD36 localization and signalling on cells. Mutating a single residue (G12V) is sufficient to reduce CD36 interactions with several tetraspanins and integrins, transitions CD36 to larger confinement regions, and impairs SFK activation following ligand stimulation. This study relies primarily on high-resolution and single molecule imaging approaches to probe these phenomena. While interesting, much of this work is predicated on a specific model of CD36-actin interaction that is unsupported, and alternative interpretations are unexplored. In addition, some conclusions are drawn that are not supported by the existing data.

Major Issues:
1. The observation in figure 1, where CD36-CD9/151-β1 interactions are decreased, while CD36-CD81-β1 interactions are increased, in the G12V mutant is interesting and forms the basis of many subsequent experiments. However, the degree of change is not apparent or quantifiable in the colocalization networks. Additional qualification of the existing dataset should be added to more specifically demonstrate these changes (e.g. faction of total CD36 present in each complex in WT vs mutant cells). Additionally, given the central aspect of this finding to the remainder of the study, an additional approach using a non-imaging method (e.g. co-IP) would further help quantify the magnitude of this change.

2. Figure 1 & 2C-D: is molecular density truly higher at the periphery than in the center, or is this an artefact of using TIRF imaging with central regions of the cells not in contact with the coverslip and therefore out of range of the evanescent field? Comparing fixed, non-permeabilized cells in TIRF versus epifluorescence, or in TIRF fields of different depths, could resolve this question. Since this affects multiple figures and conclusions, it is critical that this be clarified. Similarly, is this spatial arrangement unique to the baso-lateral surface, or is it maintained on the apical surface as well?

3. In Figures 2 the authors look at the impact of G12V on the association of CD36 with actin, finding a decrease, but counter-intuitively find an increase in CD36 undergoing confined diffusion in Figure 3. The authors link this to changes in the coupling of CD36 to the underlying actin cytoskeleton, but the analyses supporting this used speckle microscopy which cannot detect broader changes in the cytoskeleton. Alternative hypotheses are that 1) CD36-tetraspanin interactions alter the underlying cytoskeleton to increase local actin density, or 2) CD36-tetraspanin complexes preferentially localize to regions of the cell with higher-density cortical actin (and therefore smaller confinement regions). Tetraspanins interact with ERM proteins, so it is plausible that these interactions may serve to concentrate CD36-tetraspannin complexes in regions of higher actin density. Some sort of measurement of actin and tetraspanin density at the locations of confined WT vs G12V CD36 would further delineate potential mechanism(s) accounting for this observation.

4. The claim that the "confined state in the mutant spans a larger confinement area, suggesting a weaker "force of confinement."" is unsupported. Methods to quantify the strength of confinement zones have been published (e.g. Doliwa, B. & Heuer, A. Phys. Rev. Lett. 1998), and could be performed on the existing dataset to test this hypothesis. Likewise, the experiments suggested in point #3 could help delineate the mechanism(s) of confinement.

5. The conclusion that there is "high levels of overall SFK activation at CD36 molecules in the basal state for the G12V mutant" needs to be better supported. Again, some form of quantification (e.g. faction of total CD36 co-localized with phospho-SFK, nearest-neighbor analysis, etc) would allow for direct statistical comparisons of changes in the association of CD36 with pSFK.

6. In the provided images the density of CD36 in the peripheral region appears to be consistently higher in the wild-type than in the G12V mutant. Is this accurate, or just an incidental artefact of the images that were chosen for the paper? If it is a consistent phenotype, how does it affect the colocalization measure used?

Minor Issues:
1. Table S1 spans multiple pages. It would be helpful if the column headers could be repeated at the top of each page.

2. The authors claim to use a pan phospho-SFK antibody (Thermo-Fisher 44-660A1), but this product appears to be specific to Src and not the pan-SFK family. Is there a citation or other source supporting the pan-SFK specificity of this antibody?

3. The localization of the G12V mutant to caveoli suggests that it may undergo accelerated recycling/internalization. If so, this would indicate a role for the tetraspanin/integrin interactions in stabilizing CD36 on the cell surface. Can this be assessed directly?

4. "Abstract" is spelled incorrectly on page 2.

Thank you and the reviewers for the thoughtful feedback on our manuscript, and for giving us the opportunity to resubmit our work to be considered for publication in JCB. We appreciate the points raised by the reviewers and have revised our manuscript accordingly. We feel that the manuscript has become significantly stronger thanks to the changes and additions prompted by the reviewers' comments. Below we provide a point-by-point response to the reviewers' comments (reviewer text in blue, our response in black). In addition, to aid in the evaluation of the revised manuscript, we have highlighted where possible within the manuscript the changes in yellow.

Here we also provide a summary of the major changes to the manuscript in response to the reviewers' comments:

1. We performed complementary experiments to validate our colocalization analysis results and further probe the role of CD36-β_1-integrin-tetraspanin interactions in linking CD36 to cortical actin and regulating its confinement. These experiments include:
    a. Proximity ligation assay (PLA), which confirmed reduced CD36-β1-integrin interactions in the G12V mutant (Fig. S5).
    b. Three-color imaging of CD36, β1-integrin or CD9, and actin, which showed that CD36 associated with these partners is located in regions richer with actin than CD36 not associated with these partners (Fig. S3).
    c. Single-molecule imaging of CD36 together with continuous labeling of actin (using SiR-actin) in live cells, which showed that confined WT CD36 tracks are in regions of higher-than-average actin, but confined mutant CD36 tracks are not, consistent with our conclusion that actin plays a diminished role (if any) in regulating CD36 mobility in the presence of the G12V mutation.
2. We performed additional analysis of the confinement strength of WT and mutant CD36, using the method of Doliwa, B. & Heuer, A. Phys. Rev. Lett. 1998, as suggested by the reviewers.
3. We have expanded explanations of our approaches and clarified key concepts throughout the manuscript (Results and Materials & Methods) so that this manuscript is now more self-contained, reducing the need for readers to refer to previous publications regarding colocalization analysis (Vega-Lugo et al., 2022) and SMI-FSM (Dasgupta et al., 2023). These revisions also clarify how factors such as molecular density, expression level, and labeling efficiency are accounted for in the analysis.
4. We revised our use of terms such as "nanodomains" and "complexes" to more accurately reflect the scope of our data. We now primarily use the term "interactions". We also explicitly define the term "cooperativity" within the context of conditional colocalization analysis.
5. To better quantify the differences in the extent of colocalization for different molecular pairs or triplets between WT and mutant, we now complement -log10(*p*-values), which describe the significance of colocalization, with effect size measurements, specifically Cohen's d. Together, the -log10(*p*-values) and Cohen's d provide a more quantitative and interpretable basis for comparing colocalization across conditions.

Reviewer #1:

In this manuscript, Jana et al apply several advanced imaging techniques in fixed and living cells combined with different data analysis algorithms to resolve interactions between CD36, b1-integrins and different

tetraspanins. The hypothesis of the authors is that Interactions between CD36, b1 integrins and tetraspanins nanodomains mediate the link between CD36 and cortical actin. By taking advantage of a point mutation of CD36 which abolishes the interaction of CD36 with the tetraspanin CD9, the authors then investigate how the extent of this mutation (and thus interaction with CD9) perturbs the interaction with integrin b1 and cortical actin. There are several studies recently performed by some of the authors in this work and by other groups that have already revealed a role for cortical actin regulating the spatiotemporal organization of receptors located at the cell membrane, even for those with no direct interaction with actin. Thus, although the concept is not new, the work presented in this manuscript is interesting and it nicely adds to the emerging view that multi-molecular interactions are crucial to initiate signalling and contribute to receptor function. There are however a number of statements and conclusions not fully supported by the experimental data provided so far, requiring further experiments before the paper could be considered for publication. In addition, many more details should be provided regarding the data analysis methodology, in order to make this manuscript more accessible to the biologist readership.

Major comments that require revision:

1) Page 4: the authors should carefully explain how the conditional colocalization analysis is performed, rather than simply refereeing to the paper by Vega-Lugo et al.

We agree that a more detailed explanation would improve accessibility of the manuscript. Accordingly, we have added a more detailed description of the method and its interpretation in the results section (lines 103-122) as well as in the legend of Fig. S2.

2) Density distribution and degree of co-localization: It is not clear whether the experiments shown in Figure 1, and supplementary figures S1 & S2 have been performed at saturating labelling densities or sub-labeling conditions to identify individual puncta in the diffraction limited TIRF images. In particular, the degree of colocalization is largely affected by the labeling densities of the molecules involved. How the expression levels of the different proteins involved and their labeling have been taken into account for the analysis? From the quantification of Fig. S1, there is higher expression levels of all the molecules investigated at the periphery and thus a "simple" colocalization analysis would render higher coincidence colocalization of these partners just based on densities, and not as a result of any active process going on.

The reviewer is correct that the density of molecules affects their colocalization extent. We are aware of this, and our colocalization method takes this into account in the control measure termed “nullTR.” NullTR is a measure of the coincidental colocalization fraction in the absence of any true relationship between the target and reference molecules (the molecular pair under investigation). To estimate NullTR, target points are represented by points on a grid, which thus calculates the extent of colocalization expected by chance, given the density and distribution of reference points. This is also why we often report the extent of colocalization in terms of “-log10(p-value)”, derived from comparing the colocalized fraction of the experimental data to the coincidental colocalized fraction nullTR. The colocalized fraction derived from the experimental data is not meaningful on its own; its interpretation requires comparison to the coincidental colocalized fraction (nullTR). We have explained this procedure in more detail in the revised manuscript (Lines 103-118, Results section and Lines 802-803, Methods section).

To answer the reviewer’s first question, the labeling is not at saturating densities (lines 609-610). Again, even more so requiring the comparison to nullTR to decide whether an observed colocalization fraction is

significant or not. We estimate that, for CD36 at least, the labeling fraction is 20-30% (estimated from dual labeling of CD36 with two independent labels; see Fig. S4C in the revised manuscript for example). We have now explicitly stated how the interpretation of conditional colocalization analysis is not affected by differences in object density between experiments (lines 113-115).

3) Along these lines, I find Figures 1F,G and Figure 1M,N extremely difficult to understand and the figure caption does not help much. Although the plots shown in Figure S2 are intended to provide the quantification of CD36 colocalization with the different partners, the explanations provided in the caption and/or methods are quite confusing. For instance, the title of Figure S2A refers to CD36wt colocalization with b1 integrin, but the x-axis shows "overall", CD36 w/CD9 and b1-integrin w/CD9 ... what does this exactly means? What is overall?, what are nullTR, randC? How does the statistical significance has been calculated?. Same comments apply to the rest of the panels.

We apologize for the confusion in the original figures and accompanying explanations. In response, we have substantially revised both the figure legends and the manuscript text to improve clarity. We have expanded the explanation of the conditional colocalization analysis, including adding definitions of the plotted quantities such as "overall," "nullTR," and "randC," as well as a detailed description of how statistical significance is calculated (See Fig.S2 legend, Results - lines 103–118; Methods section "Statistical testing for colocalization analysis"). We hope that these revisions improve the interpretability of Figs. 1F, G, M, N and Fig. S2 (which shows only the case of CD36 colocalization with β_1-integrin, conditional on CD9).

4) Page 5: the authors give for granted that these proteins exist in nanodomains without providing experimental evidence for this. This should be validated with additional experiments & analysis. In particular, labeling of tetraspanins without inducing artifacts is quite challenging... In addition what do the authors mean exactly by the term "cooperativity". Along these lines, there is also no evidence of b1-tetraspanins nanodomains? Are they complexes? or are these two separate nanodomains in close proximity to each other? Is this what the authors refer to as cooperativity?

We agree that our original wording may have given the impression that we were resolving these multimolecular assemblies at a higher resolution than allowed by our current methods. Instead, our intent was to place our findings in the context of prior evidence that tetraspanins, which facilitate CD36-β_1-integrin association, are known to form "tetraspanin enriched microdomains (or nanodomains)." In this study, we investigated CD36's association with β_1-integrin and tetraspanins based on evidence of physical interactions provided by multiple previous studies, cited in our manuscript. Several groups used methods such as co-IP to demonstrate interactions between CD36 and β_1-integrin and between CD36 and various tetraspanins, including in vascular endothelial cells (Heit et al., 2013; Huang et al., 2011; Kazerounian et al., 2011; Miao et al., 2001; Primo et al., 2005; Thorne et al., 2000). These interactions are now demonstrated further in the work from the Touret lab ((Saini et al., 2025) BioRXiv preprint). Beta1-integrin-tetraspanin interactions have been shown previously as well (Berditchevski, 2001; Charrin et al., 2009; Yauch et al., 2000; Zhang et al., 2009) (also cited in our manuscript).

Having said this, the concept of nanodomain is not necessary for our findings. Therefore, we have reworded sentences that previously used the terms "nanodomain" or "complex" to now use the term "interactions" and occasionally the term "molecular assemblies" (where needed, and clearly defined; please see lines 63 and 145-150).

As for the term cooperativity, in our study it reflects our results that when CD36 is with one molecule type (e.g., β_1-integrin), it is highly likely to be also with another molecule type (e.g., CD9). Our conditional colocalization analysis indicated that the CD36 molecules that are colocalized with β_1-integrin are more likely to be also colocalized with CD9 than the overall population of CD36 molecules (i.e., with or without β_1-integrin), and the CD36 molecules that are colocalized with CD9 are more likely to be colocalized with β_1-integrin than overall population of CD36 (i.e., with or without CD9). In other words, being with one molecule increases the chance of being with another molecule, thus the term cooperativity, suggesting a 3-way complex. We now define this concept more explicitly in the revised manuscript (lines 103–108). Importantly, we do not assume a specific spatial organization (e.g., pre-formed nanodomains or spatially proximal interactions), and our conclusions are compatible with multiple possible arrangements, including multimolecular complexes or closely associated molecular populations.

5) The expression levels of the G12V are approximately two-fold lower than that of CD36, in particular at the cell periphery (Fig. S1). How does this impact the degree of co-localization between the different partners?

The density concern is similar to Point 2 above. Please see the response at Point 2.

The overall expression level of wildtype and the mutant are actually rather comparable (see Fig. S1C, H of the submitted manuscript). Their densities are quite different in the periphery region, which can lead to the concern about the effect of density on our colocalization analysis, but again please see the response to Point 2 above explaining how density is taken into account.

6) Page 7: More detailed explanations on the MVRG analysis should be provided.

We have now provided more detailed information on both how the data for MVRG analysis are obtained (see lines 218-231) and our interpretation of this analysis (lines 251-254). Further, the sections Live-cell SMI-FSM analysis and Statistical testing for SMI-FSM provide now further details for understanding and interpreting our MVRG analysis, see lines 866-876.

7) Page 7, lines 187-189: I don´t agree with this statement. The mutation results shown in Fig. 2A,B indicate that the weakened coupling with CA is mediated by a reduced interaction with CD9, but it DO NOT provide evidence that the complexes contribute to the link between CD36 and CA. If I understand correctly Fig. 2A,B the differences between the WT and the mutant are more pronounced at low speckle density and in the cell center, while no differences between WT and mutant are observed at the cell periphery. Yet, the enrichment of integrins occurs preferentially at the cell periphery. Thus, whether integrins (nanodomains) are involved in this process is not granted by the results provided here, on the contrary.

In line with our response to previous comments, we have modified the terminology used in our manuscript regarding complexes, to better reflect the scope of our data. We now largely use the term interactions, without the need for invoking complexes or nanodomains.

Regarding the interpretation of Fig. 2A, B, we would like to respectfully correct the reviewer's statement. The coupling between CD36 and actin speckle displacement is fully abolished in both center and periphery for the mutant, in regions of low speckle density. Therefore, the effects of the mutation are felt all throughout the cell, including in the periphery where integrins are enriched. To improve clarity of Fig. 2A, we have revised the figure, by removing the statistical comparison between $CD36^{WT}$ and $CD36^{G12V}$ in the sparse-center condition. In this case, the mean of the single cell regression coefficients (SM MSS slope) for

the G12V mutant is not significantly different from zero, which already indicates a loss of coupling with cortical actin (while the mean for WT is significantly different from zero). This modification ensures consistency in the presentation of statistical analyses across datasets, for results involving MVRG analysis.

Please also see our response to the next comment regarding the role of β_1-integrin and its interactions with CD36.

8) Page 8, lines 195-200. The reasoning is likely to be true, but it would need to be shown in a more robust and quantitative manner. The involvement of b1 integrins, for instance, is not demonstrated at all here. One way to assess the role of integrins and tetraspanins in this process would be to perform dual color SPT experiments between b1 and CD36wt and between b1 and the mutant. Alternatively, high-density multi-color SPT experiments as recently implemented by Mateos et al, ACS Nano 2024 could directly assess real-time interactions between different partners at the single molecule level. The authors could follow a similar approach or at least discuss the advantage and limitations of their methodology as compared to these new approaches (including the work from Niederauer et al. Nat. Comm. 2023: Dual-color DNA-PAINT single-particle tracking enables extended studies of membrane protein interactions).

We thank the reviewer for these suggestions of advanced multi-color single-particle tracking approaches to observe molecular complexes in live cells. However, implementing such approaches in our system presents significant technical challenges. In particular, labeling β_1-integrin for live cell single molecule imaging without perturbing its function is non-trivial and may itself alter integrin mediated interactions and signaling (Lakoduk et al., 2020). Furthermore, robust dual-color SPT analysis would require substantial methodological development, including new analysis pipelines, which is beyond the scope of the current study.

In the interest of time and in the hope of keeping our manuscript succinct, we performed alternative complementary experiments and analyses to support our conclusions, especially regarding the role of beta1-integrin. First, working with the Touret lab, we have performed proximity ligation assay (PLA) between CD36 and β_1-integrin for the G12V mutant, and verified that the G12V mutation indeed reduces CD36-β_1-integrin interactions, as suspected from our colocalization analysis results (Fig. S5). PLA probes interactions at a higher spatial resolution than two-color TIRF imaging, thus validating our observation with the colocalization analysis. Second, we have performed 3-color imaging experiments of CD36, β_1-integrin and actin, as well as CD36, CD9 and actin (actin stained with phalloidin). Using these data, we tested whether the CD36 molecules colocalized with β_1-integrin (or CD9) are more likely to be in actin-rich areas than CD36 molecules not colocalized with β_1-integrin (or CD9), and found that indeed this is the case (Fig. S3B, C). These results support a role for β_1-integrin and its interactions with CD36 in mediating the link between CD36 and cortical actin. We thank the reviewer for prompting us to perform these experiments and analyses, which further validated our original observations and conclusions.

9) An important experiment to be performed here is identifying the role of integrins in this process (related to the discussion provided in page 13), as integrins could provide the link between the PM and the CA, only when active. Thus, comparison of the results obtained under conditions that keep b1 active and non-active would clarify the mechanism of coupling to CA.

To address this concern, we performed colocalization experiments for CD36 and activated β_1-integrin, using an antibody that recognizes the active form of β_1-integrin [12G10]. We found that CD36 exhibits significant colocalization with activated β_1-integrin (Fig.S3A). We also probed CD36-active β_1-integrin interactions using PLA, and found that $CD36^{WT}$-active β_1-integrin resulted in significantly higher PLA signal compared to PLA signal from $CD36^{G12V}$-active β_1-integrin (Fig.S5, and (Saini et al., 2025)). Please note that the cells in our experiments are plated on fibronectin-coated dishes, which activates β_1-integrin.

Minor comments:

1) Page 3, lines 57-60: The paper by Gutierrez-Martinez eLife 2023 provides further proof of receptors lacking specific actin binding motifs and still their spatiotemporal organization is influenced by the cortical actin. It might be worth citing.

Thank you. We have cited this paper.

2) As the data provided are quite complex to interpret, it is imperative that the captions associated to the figures are explained in much better detail.

We have modified the captions and legends of both main and supplementary figures; we hope this makes them more accessible.

Reviewer #2:

In this manuscript, Jana et al. investigate the organization of CD36 at the plasma membrane, with a particular focus on its colocalization with integrins and tetraspanins, as well as its association with the cortical actin cytoskeleton. The study uses a previously published conditional colocalization analysis by the same group to examine the spatial distribution of these molecules, alongside speckle analysis to assess the dynamic behavior of CD36 in relation to the actin cytoskeleton. A mutant form of CD36 (G12V), predicted to exhibit reduced binding to CD9, is used to evaluate the effects of altered CD36-tetraspanin interactions on both its distribution and actin association. The authors propose a previously uncharacterized connection between these molecular components and present data on downstream signaling events affected by perturbation of this interaction.

Overall, the manuscript provides new observations on the organization of membrane-associated proteins. Yet, the main conclusions rely exclusively on two experimental approaches (colocalization and speckle analyses) that, by itself, do not provide sufficient evidence to fully support the claims of the authors. Also, several choices with respect to parameter selection and data analysis are made without adequate justification. The impact of alternative parameter selections is also not addressed. The manuscript is very technical and it is not always evident how the reported findings translate to broader molecular behavior in cells. Moreover, conclusions about molecular interactions are based on a single CD36 mutation that is not thoroughly characterized. It remains unclear to what extent this mutant retains interactions with other proteins, something that is also not clarified in the current manuscript. Lastly, the absence of functional data on cellular behaviour limits the advance in understanding of how altered CD36 behavior may influence cellular outcomes.

Please see our detailed responses below to address these concerns point by point as they come up. Briefly we have employed proximity ligation assay to verify CD36-β_1-integrin interactions and their disruption by

the G12V mutation (Fig. S5). Also, as discussed in the introductory section of this response document and in the context of reviewer 1 (as well as in the detailed responses below), the work from the Touret lab ((Saini et al., 2025),BioRXiv preprint) demonstrates many of the same interactions. In addition, we revised parts of the manuscript to make it less technical. As for functional data, we focused on the effects of the mutation on the initial steps of CD36 signaling in order to keep the manuscript succinct. The Touret lab manuscript just mentioned also shows similar effects, in their case through shRNA-mediated depletion of CD9 or β_1-integrin.

The following comments focus selectively on the colocalization analysis, but comparable concerns apply to the speckle analysis section. Also for the speckle analysis, several choices, such as the classification of high versus low speckle density, are not well substantiated and complicate data interpretation. Furthermore, the use of non-intuitive parameters (e.g., the MSS slope derived from MVRG analysis) introduces challenges for deriving clear biological meaning from the results.

We apologize for not better explaining the rationale behind certain key parameters and thresholds, which have been established in previous publications, in this manuscript. We have made every effort to make our analysis choices and rationales clearer in the revised manuscript, for both the colocalization analysis and the MVRG analysis. Please see lines 239-242 for the justification of speckle density threshold, and lines 228 to 232 for a description of the receptor property MSS slope.

Colocalization analysis (Figure 1/5): 1. The first section of the manuscript aims to study the "interactions of CD36 with integrins and tetraspanins in their cellular context". For this, the authors use a previously published conditional colocalization analysis from the same group to quantify the organization of these membrane proteins. Although sophisticated, there are many open questions related to this conditional colocalization analysis and related choices from the authors that complicate the interpretation of the conditional colocalizaton analysis.

First, the authors use a colocalization radius of 2 pixels (162 nm). The rationale for selecting this specific radius is not clearly stated, nor is it discussed how selecting a different radius would affect the outcome of the analysis. The manuscript states that the 2-pixel radius increases "the accuracy of the analysis," but the basis for this claim and the supporting data are not provided.

We appreciate the reviewer’s concern, and we explain in more detail now in the manuscript how the smaller the colocalization radius, the more specific is the colocalization analysis (we also use the term specificity now, and not accuracy). Please see lines 783-785. Briefly, it is desirable for colocalization analysis to use a smaller colocalization radius, as this reduces the chance of false positive colocalization events. Thus, 2 pixels is more specific than 3 pixels. However, at the same time, if there are registration shifts between the channels (as is the case in our data, which we correct for but unfortunately not 100%), using a too small colocalization radius might lead to false negatives. This is why for our data a 1-pixel colocalization radius is insufficient, and the 2-pixel radius provides the best balance, minimizing false positives and false negative.

To demonstrate this, here we show the results of conditional colocalization analysis for one triplet of molecules (CD36, CD9 and β1-integrin) in the periphery region using three colocalization radii, namely 1, 2 and 3 pixels. The below table shows the -log10(p-value) for p(TwR) (colocalization of target with reference regardless of condition), p(TwR|TwC) (colocalization of condition-positive target with reference), and p^{rs}(TwRwC) (colocalization of target with condition-positive reference), using the three colocalization

radii. -log10(p-value) shown in blue surpass the significance threshold (as done in the manuscript). NaN entries indicate not enough data to calculate the indicated conditional colocalization measures.

Target	Reference	Condition	-Log 10 pValues								
			pTwR			p_TwR_TwC			p^rs_RwC		
			1 pixel	2 pixel	3 pixel	1 pixel	2 pixel	3 pixel	1 pixel	2 pixel	3 pixel
CD36 WT	β1	CD9	**2.55**	**6.52**	**7.42**	NaN	**3.03**	**5.53**	NaN	**1.97**	1.61
β1	CD36 WT	CD9	**1.68**	**2.20**	**2.03**	NaN	0.30	0.47	NaN	**2.08**	**2.25**
CD36 WT	CD9	β1	**1.73**	**3.01**	**6.40**	NaN	**3.48**	**5.32**	NaN	0.58	0.46
CD9	CD36 WT	β1	**1.63**	**2.20**	**2.67**	NaN	0.02	0.09	NaN	**4.44**	**2.36**
CD9	β1	CD36 WT	**11.19**	**11.19**	**11.2**	NaN	0.25	0.19	NaN	0.95	0.77
β1	CD9	CD36 WT	**10.98**	**11.19**	**10.7**	NaN	1.54	1.66	NaN	1.17	0.79

As can be seen in the table, the results for p(TwR) are consistent throughout all radii. As for the conditional measures p(TwR|TwC) and p^{rs}(TwRwC), these are consistent between 2 and 3 pixels, but suffer when performing the analysis using a 1-pixel colocalization radius due to insufficient data. These trends are consistent with our previous work (Vega-Lugo et al., 2022).

As our analysis across multiple colocalization radii is consistent with our previous work and did not alter any of the main conclusions, we have chosen to include these results only in the rebuttal rather than in the main article.

2. Second, the authors have chosen to separate their analysis between the center and the periphery of the cell (a band of ~4um from the edge). The two reasons that the authors give are "to avoid artifactual colocalization due to large-scale molecule co-distribution" and that "the periphery had substantively different actin cytoskeleton architecture than the center". Both arguments, however, raise questions about the strength of the analysis and the reason why 4um is chosen as a cutoff.

We explain now in more detail our choice of 4 um to separate between periphery and center (see lines 119 to 129 and 513 to 517). In a nutshell, 4 um was based on earlier work (Delorme et al., 2007) and our own previous work (Dasgupta et al., 2023), where we saw differences between the two regions (periphery, defined as < 4 um from the cell edge, and center, defined as the rest of the imaged surface area) in terms of cortical actin and CD36 behavior. In addition, from a biological perspective, the cell edge represents a "free" edge that is not in contact with neighboring cells, and may resemble the leading edge of spreading, migrating, or sprouting endothelial cell, processes relevant to angiogenesis. These considerations suggest

that the peripheral region is likely to exhibit distinct actin architecture and molecular organization. We have now included these reasonings explicitly in the revised manuscript. In fact, in our work, continuous staining of actin via phalloidin shows that, in many cells, this 4 um band from the cell edge shows qualitatively different actin structures than the rest of the imaged surface (Fig. S1B). In some cells, this band is thicker, but rarely is it thinner. Therefore, we selected 4 µm as a practical compromise, keeping the periphery region as distinct from the center region as possible. We have also updated Fig. S1B to better illustrate the different actin architecture within the periphery region.

We would also like to clarify that 4 um is not meant as a sharp cutoff between two distinct and nonoverlapping regions. It is well possible that the change in behaviors and relationships occurs gradually with distance from the cell edge. Nonetheless, spatial heterogeneity is clearly present, and this segmentation allows us to capture these differences in a systematic manner. To further assess the robustness of this choice, we repeated some of the key colocalization analyses ($CD36^{WT}$, β_1-integrin and CD9) using a range of cutoffs for the periphery region (2-6 um), to investigate how that would affect our results. The results are shown in the following figure.

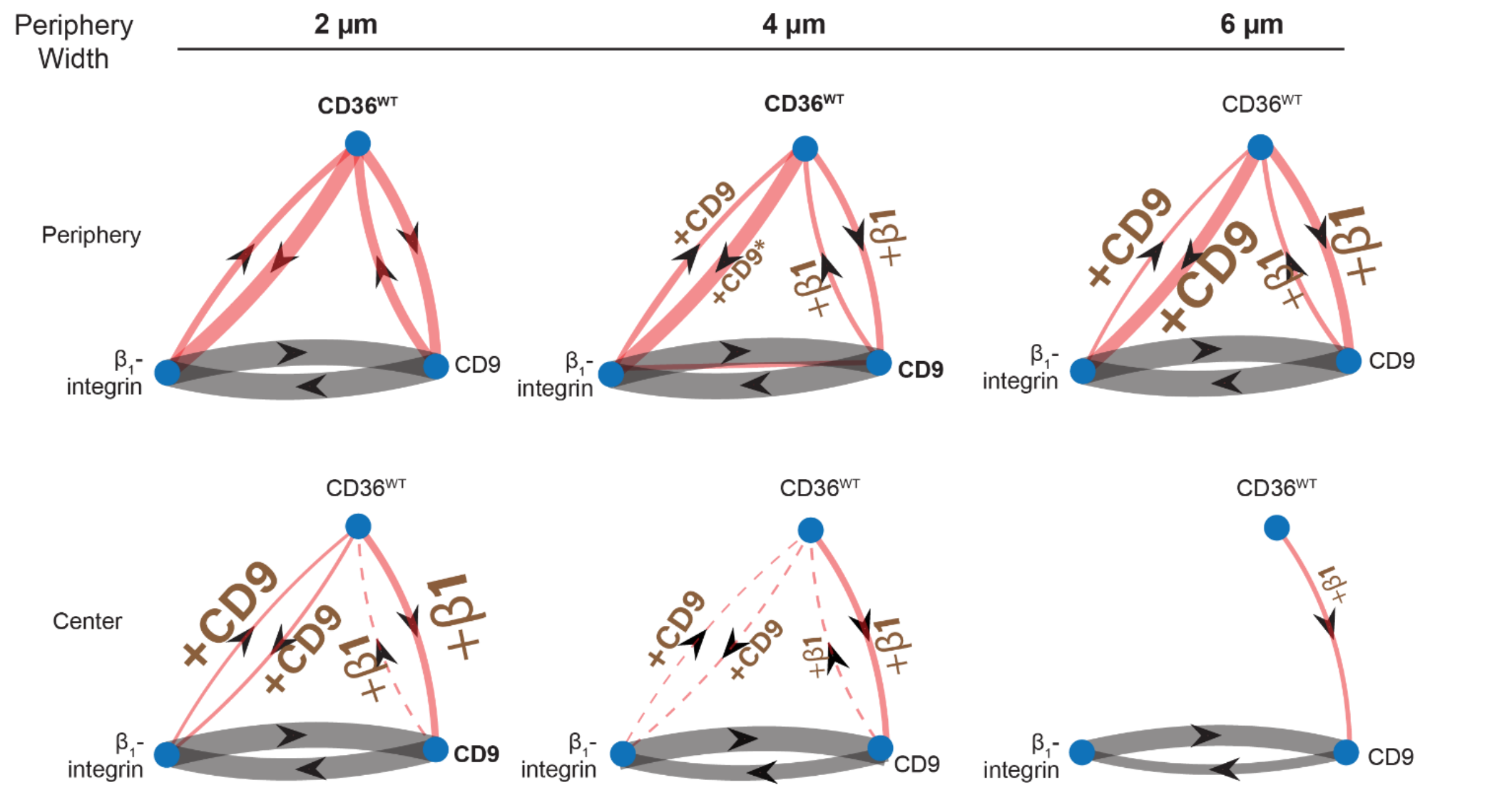

Legend: **CD36 colocalization networks with β1-integrin and CD9 in peripheral regions of different widths.** Schematic representation of CD36^{WT} colocalization networks at increasing peripheral widths (2 µm, 4 µm, and 6 µm, respectively), both in the periphery region (top row) and in the central region (bottom row). Colocalization analysis was performed using a 2-pixel colocalization radius for all cases. Nodes represent CD36^{WT}, β_1-integrin, and CD9. Solid arrows indicate significant colocalization relationships, pointing from target molecule to reference molecule ($p \leq 0.05$). Network arrows involving/not involving CD36^{WT} are shown in red/gray, for visual clarity. Arrow thickness reflects the relative strength of colocalization. Labels adjacent to arrows (e.g., +CD9 or +β_1) indicate conditional enhancement, meaning colocalization between two molecules is significantly increased within the subset of CD36 detections colocalized with the indicated molecule. Across panels, absence of an arrow indicates no significant colocalization detected under the tested condition. Peripheral width defines the distance from the segmented cell edge used to classify detections as periphery or center populations.

As can be seen in the figure, pairwise colocalization relationships in the periphery region are preserved across all periphery widths, while the influence of the third conditional molecule (or evidence of cooperativity) goes from negligible to being more pronounced as the periphery region expands. The latter is likely due to increased sampling as the periphery width increases (in particular, the 2 um periphery region might provide too little data to robustly assess conditional colocalization; equivalent to our observation in Point 1 above when using a too small colocalization radius). Interestingly, in the center region, both pairwise and conditional relationships decrease as the peripheral region expands and the center region correspondingly shrinks. This is most likely because the interactions between these molecules is strongest near the cell edge, and it decreases further away from the edge. All in all, these

results using different periphery widths are consistent with our conclusion from our original analysis. Therefore, for the sake of manuscript succinctness, we decided to include them only in the response letter.

We would also like to explain in more detail what we mean by the statement "to avoid artifactual colocalization due to large-scale molecule co-distribution." In fact, the issue is not artifactual colocalization, but rather inflated significance of any observed colocalization. The reason for the inflated significance due to large scale molecule co-distribution is best explained with an example. Let's take two molecular entities, both of which primarily reside within a subcellular area (such as the periphery region). If the colocalization analysis is done over the whole cell, in which case the calculation of nullTR replaces the target points with points on a grid throughout the whole cell, this would lead to a low fraction of coincidental colocalization, which would overestimate the significance of the colocalized fraction in the data. However, if the colocalization analysis is focused on the area where both molecules are enriched, then the coincidental fraction would not be low, and the comparison between data and nullTR – and thus the estimated colocalization significance – would be more relevant and less inflated. To avoid any confusion, we have now reworded the section describing our choice of 4 um to separate between periphery and center to focus primarily on the biological reason, as that is sufficient motivation to analyze the two regions separately, even if they had no density differences (see lines 119 to 129 and 512 to 516)

3. Specifically, the first argument suggests that the density of the investigated molecules could influence the colocalization values. Yet, the authors for example report stronger colocalization in the periphery as compared to the center, but if density affects colocalization, the interpretation of this difference is unclear. Also, the authors indicate that the distribution for CD36 changes with the G12V mutation but it is not discussed how this altered distribution could influence the colocalization values. It is therefore not obvious what higher colocalization (p-)values mean for the actual proximity of the two/three proteins. Also, since the CD36 is introduced by overexpression, it raises the question of how a specific expression level was chosen and how it was ensured that cells with similar expression values were picked. And how does the CD36 WT/CD36 G12V overexpression compare to native CD36 levels?

The density question is similar to the concern raised by reviewer 1. Please see our responses above at Points 2 and 5 of Reviewer 1.

Regarding the expression level of CD36, Fig. S1C, H of the revised manuscript show the density of detected CD36 spots in the imaged cells, which reflects their expression levels. These plots show that the expression levels vary by a small amount between the cells selected for imaging, for both wildtype and mutant CD36. As for how this expression level compares to endogenous CD36 expression levels, we have now compared the expression levels of CD36 by immunofluorescence and found that the expression levels were comparable between the CD36WT in TIME cells and endogenous CD36 in Human dermal microvascular endothelial cells (HDMECs) (both labeled using the same anti-CD36 antibody) (new Fig. S1A). This has now been explicitly stated in the revised manuscript – section "Comparison of CD36 expression levels between endogenous and transient overexpression" in the materials and methods section, also see lines 95 to 97 and 600 to 602. Note that microvascular endothelial cells in culture lose their CD36 expression upon passaging (see., e.g., Fig. S3 in Dasgupta et al. BJ 2023), and the TIME cells used in our experiments have lost almost all endogenous CD36 expression. Therefore, it is reasonable to compare our exogenous CD36 expression levels to those of endogenous CD36 in early passage HDMECs.

4. The second argument considers actin concentrations towards the periphery, but this also lacks sufficient clarification. The image in Figure S1F does indeed show a higher actin intensity but not all of the observed actin is cortical actin. In the image, actin fibers and other actin related structures are observed at the periphery that would not necessarily affect CD36 diffusion or directly couple to CD36. Aside from these specific issues with the presented arguments, the reason for precisely selecting 4 µm as the boundary is not explained and whether it corresponds to a clear threshold in actin and/or molecular distribution is not addressed.

It is possible that some of the observed actin is not cortical, although the actin cortex is thought to be 100-200 nm thick in mammalian cells (Chugh and Paluch, 2018), which is thicker than the region imaged by TIRF illumination (which has a penetration depth of ~90 nm). Stress fibers are part of the cortex as well (Vignaud et al., 2021). Having said that, even some of the cortical actin might not be affecting CD36 or might not be coupled to it. This is exactly what we quantify using our SMI-FSM analysis. And our observation is that there is some coupling between CD36 mobility and actin speckle behavior in the wildtype case, and this coupling is reduced for the G12V mutant.

As for the choice of the 4 um boundary between periphery and center, please see the response at Point 2 above (of this reviewer).

5. Furthermore, and importantly, the presented colocalization alone does not allow the investigation of molecular interactions. At best, it indicates spatial proximity. To demonstrate molecular interactions more directly, complementary approaches such as dual-color single-particle tracking, fluorescence fluctuation spectroscopy, or biochemical methods like co-immunoprecipitation would be required.

We appreciate the reviewer's concern, and we performed proximity ligation assay (PLA) to demonstrate more directly CD36-β1-integrin interactions and their disruption by the CD36 mutation (Fig. S5). Please note that PLA has a higher spatial resolution (40 nm) compared to colocalization analysis (~200 nm). For more details, please see our response to comment 8 by Reviewer 1. At the same time, as discussed at comment 4 of Reviewer 1, earlier studies, cited in our manuscript, used methods such as co-IP to demonstrate interactions between CD36 and β1-integrin and between CD36 and various tetraspanins (such as CD9), including in vascular endothelial cells (Heit et al., 2013; Huang et al., 2011; Kazerounian et al., 2011; Miao et al., 2001; Primo et al., 2005; Thorne et al., 2000). These earlier studies were in fact the starting point for our work, as stated in the manuscript introduction. These interactions have also been demonstrated further in the work from the Touret lab (using proximity ligation assay and shRNA-mediated depletion; Saini et al. 2025 [BioRXiv]).

6. Lastly, the statistics and presentation of the colocalization values require further clarification. While significant p-values suggest that the observed distributions differ from random expectations, it is unclear whether and how these p-values can be compared across conditions. For example, the authors state that the G12V mutation lowers the interaction of CD36 with CD9. Yet, both in the center and the periphery, CD36-CD9 interactions remain significant. It is therefore unclear how the authors reach the conclusion that the colocalization is less. The same is true for for example the Caveolin localization where the conclusion that the G12V mutant colocalizes more seems to be based on an p-value increase from 1.4 to 1.7. Furthermore, other remarkable findings, for example the increased colocalization between B1 integrin and CD81 in the CD36G12V conditions, are not discussed but again, raise questions about the approach

since it is not immediately obvious why this would occur. Finally, the p-values for for example the CD36/B1 colocalization change quite considerably depending on the colabel (6.5, 2.9, 5.4), and it is unclear how that should be interpreted or what the origin of these differences are.

We have expanded and revised the manuscript to strengthen our ability to compare colocalization extents between different conditions, regions, etc. To complement the statistical testing of the colocalization experiments we now also report effect sizes (Cohen's d) for the colocalization analyses (Maher et al., 2013; Morgan, 2025). Specifically, for each conditional colocalization measure or pairwise colocalization measure derived from experimental data, we compare it to its respective pairwise or coincidental counterpart using the "robustcohen" algorithm (MATLAB function, meanEffectSize()). This has been incorporated into the manuscript in table S1 and Fig.S8, and described throughout the manuscript as needed (for e.g., see lines 802-808).

As the reviewer has correctly inferred, we have taken the -log10(p-value) as our measure for how far an observed colocalization is from what is expected by chance. The higher the -log10(p-value) value, the further is the colocalization from chance, and the more significant it is. Based on this, we reached the conclusions mentioned by the reviewer, e.g., that CD36-CD9 colocalization is lower for the mutant (in the center, -log10(p-value) = 2.7 for WT and 1.4 for mutant; in the periphery, -log10(p-value) = 3 for WT and 1.8 for mutant). This interpretation is now further supported by the effect size analysis: for CD36-CD9 colocalization, Cohen's d decreases from a strong effect of 0.83 and 0.85 in WT (center and periphery, respectively) to a medium effect of 0.50 and 0.70 in the mutant, consistent with a reduction in colocalization strength. Similarly, for CD36-caveolin colocalization, while the increase in -log10(p-value) from WT (1.4) to mutant (1.8) seems modest, the corresponding effect size shows a substantial corresponding change in the same direction, increasing from a small effect of 0.43 in WT to a moderate effect of 0.57 in the mutant (Fig. S8). We agree that p-values alone are not sufficient to convey the magnitude of these differences, and for this reason we have added the effect size analysis to provide a another basis for comparison across conditions. Categorization of effect sizes into small, medium and large is as per benchmarks suggested by Cohen (Cohen, 1988) and followed widely.

As for the variation in the CD36-β1-integrin colocalization -log10(-p-value) between the 3 datasets, we thank the reviewer for bringing this to our attention. The odd one out among this trio is the value from the CD36/β1-integrin /CD81 dataset (2.9). One likely contributor is experimental variability across independently acquired datasets, including possible antibody related variation. Of course, in anticipation of such experimental complexities, in every dataset WT and mutant data were prepared and acquired on the same day, using identical procedures and imaging conditions. Therefore, even if there is day-to-day variation and other such variations, WT and mutant would be comparable. In addition, because CD36-β_1-integrin colocalization was measured independently in three datasets (with CD9, CD81, or CD151 as the third label), the values reported in Fig. 1F, G, M, and N represent the average across these datasets, reducing the impact of any single outlying value on the overall conclusions.

Reviewer #3:

Summary: This study investigates the role of the association of CD36 with tetraspanins and integrins on CD36 localization and signalling on cells. Mutating a single residue (G12V) is sufficient to reduce CD36 interactions with several tetraspanins and integrins, transitions CD36 to larger confinement regions, and

impairs SFK activation following ligand stimulation. This study relies primarily on high-resolution and single molecule imaging approaches to probe these phenomena. While interesting, much of this work is predicated on a specific model of CD36-actin interaction that is unsupported, and alternative interpretations are unexplored. In addition, some conclusions are drawn that are not supported by the existing data.

Major Issues:

1. The observation in figure 1, where CD36-CD9/151-β1 interactions are decreased, while CD36-CD81-β1 interactions are increased, in the G12V mutant is interesting and forms the basis of many subsequent experiments. However, the degree of change is not apparent or quantifiable in the colocalization networks. Additional qualification of the existing dataset should be added to more specifically demonstrate these changes (e.g. faction of total CD36 present in each complex in WT vs mutant cells). Additionally, given the central aspect of this finding to the remainder of the study, an additional approach using a non-imaging method (e.g. co-IP) would further help quantify the magnitude of this change.

We agree that the differential effect of the G12V mutation on CD36 colocalization with different tetraspanins is an interesting observation and warrants further clarification and quantification. We would like to note that the behavior is more complex than summarized. The reviewer's description applies primarily to the center region. In the periphery region, the effect of the mutation on CD36 colocalization with CD81 and CD151 is the opposite of its effect in the center (while the effect on CD9 is consistent throughout both regions). However, while intriguing, this differential effect is difficult to interpret at this stage with our data. Therefore we focused our interpretations and explanations on the consistent effect, namely, the reduced colocalization of CD36 with CD9 and β_1-integrin in the mutant across the cell.

Having said the above, the reviewer's understandable request to demonstrate interactions and their perturbation through an independent method echoes the concerns of Reviewers 1 and 2. As mentioned in response to Reviewers 1 and 2, we have now performed proximity ligation assay (PLA) to assess CD36-β_1-integrin interactions (Fig. S5). This method probes molecular proximity at a higher spatial resolution (~40 nm) than multi-channel TIRF. We observe a significant reduction in PLA signal for $CD36^{G12V}$-β_1-integrin compared to $CD36^{WT}$-β_1-integrin, consistent with our colocalization analysis. These results were interpreted in the context of appropriate positive and negative controls. In addition, recent work from the Touret lab ((Saini et al., 2025)BioRxiv preprint) independently supports the existence of CD36-β1-integrin interactions. Please see detailed responses at Points 4 and 8 of Reviewer 1 and Point 5 of Reviewer 2.

To better quantify the magnitude of changes in colocalization, we have expanded our analysis in two ways. First, we now report colocalization fractions, in addition to the -log10(p-value) which determines significance. Second, we include effect size measurements (Cohen's d), calculated by comparing experimental colocalization measures to their corresponding nullTR (coincidental) distributions for pairwise analysis, and by comparing conditional colocalization measures to their pairwise counterparts to assess the influence of a third molecule. These results are now presented in Table S1 and Fig. S8. In the revised manuscript, we have further emphasized that these fractions are only meaningful in the context of the coincidental fraction, especially in situations (like ours) where not all copies of a molecule are labeled. Please see response at Point 2 of Reviewer 1 for further details about this issue.

2. Figure 1 & 2C-D: is molecular density truly higher at the periphery than in the center, or is this an artefact of using TIRF imaging with central regions of the cells not in contact with the coverslip and therefore out

of range of the evanescent field? Comparing fixed, non-permeabilized cells in TIRF versus epifluorescence, or in TIRF fields of different depths, could resolve this question. Since this affects multiple figures and conclusions, it is critical that this be clarified. Similarly, is this spatial arrangement unique to the baso-lateral surface, or is it maintained on the apical surface as well?

The higher molecule density at the periphery for wildtype CD36, β_1-integrin and the investigated tetraspanins is highly unlikely to be an artifact of imaging. Multiple other molecules in our images do not exhibit a higher density in the periphery region, even though they are co-imaged in the same cell with molecules that show this periphery enrichment. This includes caveolin, Fyn and the mutated form of CD36. All of these show enrichment in the center region, again while being imaged (in the same exact cell) with other molecules that show enrichment in the periphery region.

The question regarding the baselateral vs. apical surface is an interesting one, but we feel is beyond the scope of our current work. This comparison will be very interesting in a future study.

3. In Figures 2 the authors look at the impact of G12V on the association of CD36 with actin, finding a decrease, but counter-intuitively find an increase in CD36 undergoing confined diffusion in Figure 3. The authors link this to changes in the coupling of CD36 to the underlying actin cytoskeleton, but the analyses supporting this used speckle microscopy which cannot detect broader changes in the cytoskeleton. Alternative hypotheses are that 1) CD36-tetraspanin interactions alter the underlying cytoskeleton to increase local actin density, or 2) CD36-tetraspanin complexes preferentially localize to regions of the cell with higher-density cortical actin (and therefore smaller confinement regions). Tetraspanins interact with ERM proteins, so it is plausible that these interactions may serve to concentrate CD36-tetraspannin complexes in regions of higher actin density. Some sort of measurement of actin and tetraspanin density at the locations of confined WT vs G12V CD36 would further delineate potential mechanism(s) accounting for this observation.

We appreciate the reviewer's concern that speckles are not straightforward reporters on actin density (even though there is a correlation, based on our previous analysis – please see Fig. S2 in Dasgupta et al. BJ 2023). We thus performed additional single-molecule imaging/single particle tracking experiments of CD36 (wildtype and mutant) in cells where actin is continuously labeled using SiR-Actin. This approach reports directly on actin density, but of course not dynamics (the main advantage of speckles). With this, we investigated whether confined CD36 trajectories occur in areas of higher actin density. We found that indeed wildtype CD36 confinement occurs in areas of higher cortical actin (CA) density compared to whole cell CA density, but mutant CD36 confinement is indifferent to CA density (Fig. S7, lines 326-334, and the section "Live Cell simultaneous Sir-Actin and SMI data acquisition and analysis" in materials and methods.). These results support our original conclusion that CA plays a reduced role in the confinement of $CD36^{G12V}$ compared to $CD36^{WT.}$

4. The claim that the "confined state in the mutant spans a larger confinement area, suggesting a weaker "force of confinement."" is unsupported. Methods to quantify the strength of confinement zones have been published (e.g. Doliwa, B. & Heuer, A. Phys. Rev. Lett. 1998), and could be performed on the existing dataset to test this hypothesis. Likewise, the experiments suggested in point #3 could help delineate the mechanism(s) of confinement.

We thank the reviewer for this suggestion. We have now quantified the strength of confinement using the suggested approach (Doliwa and Heuer, 1998) for each confined single molecule tracklet and found that

the mutation resulted in $CD36^{G12V}$ experiencing weaker confinement strength compared to WT (Fig. 3H) (lines 865-869) . These results support our original interpretation that the larger confinement areas observed for the mutant reflect weaker confinement. In addition, when considered together with the continuous actin labeling experiments described above, our data suggest that the confined motion of the CD36 mutant differs from that of the WT not only in its nature but also potentially in the underlying mechanism of confinement, with contributions beyond actin-based constraints.

5. The conclusion that there is "high levels of overall SFK activation at CD36 molecules in the basal state for the G12V mutant" needs to be better supported. Again, some form of quantification (e.g. faction of total CD36 co-localized with phospho-SFK, nearest-neighbor analysis, etc) would allow for direct statistical comparisons of changes in the association of CD36 with pSFK.

To strengthen this point, we have expanded our quantitative analysis by including effect size measurements (Cohen's d) in addition to significance testing (in Table S1). We also report now the colocalized fractions in Table S1, although, as noted in the revised manuscript (Lines 112–114), direct comparison of raw colocalization fractions between WT and mutant is not by itself meaningful, because these fractions must be interpreted relative to their corresponding coincidental colocalization values. In the unstimulated condition, the pairwise colocalization between $CD36^{G12V}$ and phospho-SFK is significantly greater than expected by chance, with a -log10(p-value) of 1.8 (corresponding to p = 0.0158). The corresponding effect size is moderate (Cohen's d = 0.53, with confidence interval not spanning zero; Fig. S8), supporting the conclusion that basal phospho-SFK enrichment around the mutant is elevated relative to chance expectations. In contrast, the mutant under TSP-1 stimulation shows smaller effect sizes for pSFK enrichment around CD36 (0.47), in agreement with ess pSFK activation around the mutant upon stimulation.

6. In the provided images the density of CD36 in the peripheral region appears to be consistently higher in the wild-type than in the G12V mutant. Is this accurate, or just an incidental artefact of the images that were chosen for the paper? If it is a consistent phenotype, how does it affect the colocalization measure used?

When we select cells for imaging, we generally look for healthy cells with what seems to be an average/common expression level among the cells. This way, the analysis results are as comparable as possible between conditions. As a result, the overall density of wildtype and mutant CD36 is similar; see Fig. S1C, H of the revised manuscript. The higher density of wildtype CD36 at the periphery, and conversely the lower density of mutant CD36 at the periphery, were post-image acquisition surprises that we discovered at the analysis stage. They were not part of any criteria during image acquisition.

As for the effect of density on colocalization analysis, this echoes questions by the other two reviewers. Please see our responses at Points 2 and 5 of Reviewer 1 and Point 3 of Reviewer 2.

Minor Issues:

1. Table S1 spans multiple pages. It would be helpful if the column headers could be repeated at the top of each page.

Done, thank you for the suggestion.

2. The authors claim to use a pan phospho-SFK antibody (Thermo-Fisher 44-660A1), but this product appears to be specific to Src and not the pan-SFK family. Is there a citation or other source supporting the pan-SFK specificity of this antibody?

The Src family kinases (SFKs) are very similar in the region containing tyrosine 419, and antibodies detecting the phosphorylation of this tyrosine (even most biosensors detecting the phosphorylation of this site), an indicator of SFK activation generally cannot distinguish between the different SFKs (Mukherjee et al., 2020). These papers (Maldonado and Leyton, 2023; Peckham et al., 2016) use the same antibody that we use (but unconjugated to a fluorophore, thus called 44660G) to probe phospho-SFKs in general and phospho-Fyn, respectively. We have used this antibody to detect phospho-Fyn in a prior publication (Githaka et al., 2016). We have supported our pan phospho-SFK use of this antibody by citing prior work in the revised manuscript (lines: 402-404).

3. The localization of the G12V mutant to caveoli suggests that it may undergo accelerated recycling/internalization. If so, this would indicate a role for the tetraspanin/integrin interactions in stabilizing CD36 on the cell surface. Can this be assessed directly?

This is an interesting point that we have considered as well, and it could suggest yet another functional consequence of CD36-tetraspanin/integrin interactions. However, it is beyond the scope of our current study, which is concerned with the link between CD36 and actin.

4. "Abstract" is spelled incorrectly on page 2.

Typo fixed, thank you.

References:

Berditchevski, F. 2001. Complexes of tetraspanins with integrins: more than meets the eye. *Journal of cell science*. 114:4143-4151.

Charrin, S., F. le Naour, O. Silvie, P.E. Milhiet, C. Boucheix, and E. Rubinstein. 2009. Lateral organization of membrane proteins: tetraspanins spin their web. *Biochem J*. 420:133-154.

Chugh, P., and E.K. Paluch. 2018. The actin cortex at a glance. *Journal of cell science*. 131:jcs186254.

Cohen, J. 1988. Statistical Power Analysis for the Behavioral-Sciences - Cohen,J. *Percept Motor Skill*. 67:1007-1007.

Dasgupta, A., H.T. Ngo, D. Tschoerner, N. Touret, B. da Rocha-Azevedo, and K. Jaqaman. 2023. Multiscale imaging and quantitative analysis of plasma membrane protein-cortical actin interplay. *Biophysical journal*. 122:3798-3815.

Delorme, V., M. Machacek, C. DerMardirossian, K.L. Anderson, T. Wittmann, D. Hanein, C. Waterman-Storer, G. Danuser, and G.M. Bokoch. 2007. Cofilin Activity Downstream of Pak1 Regulates Cell Protrusion Efficiency by Organizing Lamellipodium and Lamella Actin Networks. *Developmental cell*. 13:646-662.

Doliwa, B., and A. Heuer. 1998. Cage Effect, Local Anisotropies, and Dynamic Heterogeneities at the Glass Transition: A Computer Study of Hard Spheres. *Physical Review Letters*. 80:4915-4918.

Githaka, J.M., A.R. Vega, M.A. Baird, M.W. Davidson, K. Jaqaman, and N. Touret. 2016. Ligand-induced growth and compaction of CD36 nanoclusters enriched in Fyn induces Fyn signaling. *Journal of cell science*. 129:4175-4189.

Heit, B., H. Kim, G. Cosio, D. Castano, R. Collins, C.A. Lowell, K.C. Kain, W.S. Trimble, and S. Grinstein. 2013. Multimolecular Signaling Complexes Enable Syk-Mediated Signaling of CD36 Internalization. *Developmental cell*. 24:372-383.

Huang, W., M. Febbraio, and R.L. Silverstein. 2011. CD9 tetraspanin interacts with CD36 on the surface of macrophages: a possible regulatory influence on uptake of oxidized low density lipoprotein. *PloS one*. 6:e29092.

Kazerounian, S., M. Duquette, M.A. Reyes, J.T. Lawler, K.L. Song, C. Perruzzi, L. Primo, R. Khosravi-Far, F. Bussolino, I. Rabinovitz, and J. Lawler. 2011. Priming of the vascular endothelial growth factor signaling pathway by thrombospondin-1, CD36, and spleen tyrosine kinase. *Blood*. 117:4658-4666.

Lakoduk, A.M., Z. Kadlecova, and S.L. Schmid. 2020. A functionally neutral single chain antibody to measure beta-1 integrin uptake and recycling. *Traffic*. 21:590-602.

Maher, J.M., J.C. Markey, and D. Ebert-May. 2013. The other half of the story: effect size analysis in quantitative research. *CBE Life Sci Educ*. 12:345-351.

Maldonado, H., and L. Leyton. 2023. CSK-mediated signalling by integrins in cancer. *Front Cell Dev Biol*. 11:1214787.

Miao, W.M., E. Vasile, W.S. Lane, and J. Lawler. 2001. CD36 associates with CD9 and integrins on human blood platelets. *Blood*. 97:1689-1696.

Morgan, J.L. 2025. Alternative to the statistical mass confusion of testing for "no effect". *The Journal of cell biology*. 224.

Mukherjee, A., R. Singh, S. Udayan, S. Biswas, P.P. Reddy, S. Manmadhan, G. George, S. Kumar, R. Das, B.M. Rao, and A. Gulyani. 2020. A Fyn biosensor reveals pulsatile, spatially localized kinase activity and signaling crosstalk in live mammalian cells. *Elife*. 9.

Peckham, H., L. Giuffrida, R. Wood, D. Gonsalvez, A. Ferner, T.J. Kilpatrick, S.S. Murray, and J. Xiao. 2016. Fyn is an intermediate kinase that BDNF utilizes to promote oligodendrocyte myelination. *Glia*. 64:255-269.

Primo, L., C. Ferrandi, C. Roca, S. Marchio, L. di Blasio, M. Alessio, and F. Bussolino. 2005. Identification of CD36 molecular features required for its in vitro angiostatic activity. *Faseb J*. 19:1713-+.

Saini, A., E.G. Cardona, H. Huang, S.M. Khaing, K. Klein, K. Jaqaman, O. Julien, and N. Touret. 2025. Investigation of CD36 interactome provides insights into multimolecular complexes necessary for anti-angiogenic signalling. *bioRxiv*.

Thorne, R.F., J.F. Marshall, D.R. Shafren, P.G. Gibson, I.R. Hart, and G.F. Burns. 2000. The integrins alpha(3)beta(1) and alpha(6)beta(1) physically and functionally associate with CD36 in human melanoma cells - Requirement for the extracellular domain of CD36. *J. Biol. Chem*. 275:35264-35275.

Vega-Lugo, J., B. da Rocha-Azevedo, A. Dasgupta, and K. Jaqaman. 2022. Analysis of conditional colocalization relationships and hierarchies in three-color microscopy images. *The Journal of cell biology*. 221.

Vignaud, T., C. Copos, C. Leterrier, M. Toro-Nahuelpan, Q. Tseng, J. Mahamid, L. Blanchoin, A. Mogilner, M. Thery, and L. Kurzawa. 2021. Stress fibres are embedded in a contractile cortical network. *Nat Mater*. 20:410-420.

Yauch, R.L., A.R. Kazarov, B. Desai, R.T. Lee, and M.E. Hemler. 2000. Direct extracellular contact between integrin alpha(3)beta(1) and TM4SF protein CD151. *J. Biol. Chem*. 275:9230-9238.

Zhang, X.F., S. Kazerounian, M. Duquette, C. Perruzzi, J.A. Nagy, H.F. Dvorak, S. Parangi, and J. Lawler. 2009. Thrombospondin-1 modulates vascular endothelial growth factor activity at the receptor level. *Faseb J*. 23:3368-3376.

May 21, 2026

RE: JCB Manuscript #202504156R-A

Khuloud Jaqaman
The University of Texas Southwestern Medical Center

Dear Dr. Jaqaman:

Thank you for submitting your revised manuscript entitled "Integrins and tetraspanins mediate CD36-actin coupling and regulate CD36 organization and signaling". The manuscript has been seen by the original reviewers whose full comments are appended below.

You will see that, while reviewers #1 and #3 are overall positive about the work in terms of its suitability for JCB, reviewer #2 remains more critical and still voices essential concerns that much be addressed via thorough revisions to your text before the paper is suitable for publication in JCB. We recommend including the textual revisions and additional points for the Discussion as well as adding a schematic as suggested by reviewer #2. We further encourage you to improve the explanations throughout the text in order to improve readability for a broad audience.

To avoid unnecessary delays in the acceptance and publication of your paper, please read the following information carefully.

A. MANUSCRIPT ORGANIZATION AND FORMATTING:

Full guidelines are available on our Instructions for Authors page, http://jcb.rupress.org/submission-guidelines#revised.
Submission of a paper that does not conform to JCB guidelines will delay the acceptance of your manuscript.

1) Text limits: Character count for Articles is < 40,000, not including spaces. Count includes abstract, introduction, results, discussion, and acknowledgments. Count does not include title page, figure legends, materials and methods, references, tables, or supplemental legends.

**Please note that we can allow you extra space if necessary to fully address the reviewers' comments, but we ask that you are as concise as possible.

2) Figures limits: Articles may have up to 10 main text figures.

3) Figure formatting: Scale bars must be present on all microscopy images, including inset magnifications. Molecular weight or nucleic acid size markers must be included on all gel electrophoresis. Aspect ratios of images may not be altered.

**4) Statistical analysis: Error bars on graphic representations of numerical data must be clearly described in the figure legend. The number of independent data points (n) represented in a graph must be indicated in the legend. Statistical methods should be explained in full in the materials and methods. **For figures presenting pooled data the statistical measure should be defined in the figure legends. Please also be sure to indicate the statistical tests used in each of your experiments (either in the figure legend itself or in a separate methods section) as well as the parameters of the test (for example, if you ran a t-test, please indicate if it was one- or two-sided, etc.). Also, if you used parametric tests, please indicate if the data distribution was tested for normality (and if so, how). If not, you must state something to the effect that "Data distribution was assumed to be normal but this was not formally tested."

**5) Abstract and title: The abstract should be no longer than 160 words and should communicate the significance of the paper for a general audience. The title should be less than 100 characters including spaces. Make the title concise but accessible to a general readership.

Given that your abstract currently exceeds the 160 word limit, we kindly ask you to shorten it a bit.

6) Materials and methods: Should be comprehensive and not simply reference a previous publication for details on how an experiment was performed. Please provide full descriptions in the text for readers who may not have access to referenced manuscripts.

7) All antibodies, cell lines, animals, and tools used in the manuscript should be described in full, including accession numbers for materials available in a public repository such as the Resource Identification Portal. Please be sure to provide the sequences for all of your primers/oligos and RNAi constructs in the materials and methods. You must also indicate in the methods the source, species, and catalog numbers (where appropriate) for all of your antibodies. Please also indicate the acquisition and quantification methods for immunoblotting/western blots.

8) Microscope image acquisition: The following information must be provided about the acquisition and processing of images:
a. Make and model of microscope
b. Type, magnification, and numerical aperture of the objective lenses
c. Temperature
d. Imaging medium
e. Fluorochromes
f. Camera make and model
g. Acquisition software
h. Any software used for image processing subsequent to data acquisition. Please include details and types of operations involved (e.g., type of deconvolution, 3D reconstitutions, surface or volume rendering, gamma adjustments, etc.).

9) References: There is no limit to the number of references cited in a manuscript. References should be cited parenthetically in the text by author and year of publication. Abbreviate the names of journals according to PubMed.

**10) Supplemental materials: There are strict limits on the allowable amount of supplemental data. Articles may have up to 5 supplemental figures. At the moment, you have 8 supplemental figures and we will be able to give you a bit more space in this case. Please also note that tables, like figures, should be provided as individual, editable files. A summary of all supplemental material should appear at the end of the Materials and methods section.

11) eTOC summary: A ~40-50-word summary that describes the context and significance of the findings for a general readership should be included on the title page. The statement should be written in the present tense and refer to the work in the third person.

12) Conflict of interest statement: JCB requires inclusion of a statement in the acknowledgements regarding competing financial interests. If no competing financial interests exist, please include the following statement: "The authors declare no competing financial interests." If competing interests are declared, please follow your statement of these competing interests with the following statement: "The authors declare no further competing financial interests."

**13) ORCID IDs: ORCID IDs are unique identifiers allowing researchers to create a record of their various scholarly contributions in a single place. Please note that ORCID IDs are now *required* for all authors. At resubmission of your final files, please be sure to provide your ORCID ID and those of all co-authors.

14) A separate author contribution section following the Acknowledgments. All authors should be mentioned and designated by their full names. We encourage use of the CRediT nomenclature.

Please note that JCB now requires authors to submit Source Data used to generate figures containing gels and Western blots with all revised manuscripts. This Source Data consists of fully uncropped and unprocessed images for each gel/blot displayed in the main and supplemental figures. For assays performed using capillary electrophoresis and/or immunoassay-based detection, authors should instead provide the electropherogram graph(s) for each experiment, plotting fluorescence/chemiluminescence intensity vs. molecular weight/size. Please be sure to provide one Source Data file for each figure gels, blots, and/or capillary electrophoresis assays along with your revised manuscript files. File names for Source Data figures should be alphanumeric without any spaces or special characters (i.e., SourceDataF#, where F# refers to the associated main figure number or SourceDataFS# for those associated with Supplementary figures). For traditional gels and blots, the lanes of the gels/blots should be labeled as they are in the associated figure, the place where cropping was applied should be marked (with a box), and molecular weight/size standards should be labeled wherever possible. For capillary electrophoresis assays, each trace in the graph should be color-coded and labeled to indicate which protein, gene, or sample is being measured (please try to avoid red/green combinations to accommodate our color-blind readers).

Source Data files will be made available to reviewers during evaluation of revised manuscripts and, if your paper is eventually published in JCB, the files will be directly linked to specific figures in the published article.

Source Data Figures should be provided as individual PDF files (one file per figure). Authors should endeavor to retain a minimum resolution of 300 dpi or pixels per inch. Please review our instructions for export from Photoshop, Illustrator, and PowerPoint here: https://rupress.org/jcb/pages/submission-guidelines#revised

Journal of Cell Biology now requires a data availability statement for all research article submissions. These statements will be published in the article directly above the Acknowledgments. The statement should address all data underlying the research presented in the manuscript. Please visit the JCB instructions for authors for guidelines and examples of statements at (https://rupress.org/jcb/pages/editorial-policies#data-availability-statement).

B. FINAL FILES:

Please upload the following materials to our online submission system. These items are required prior to acceptance. If you have any questions, contact JCB's Managing Editor, Lindsey Hollander (lhollander@rockefeller.edu).

-- An editable version of the final text (.DOC or .DOCX) is needed for copyediting (no PDFs).

-- High-resolution figure and MP4 video files: See our detailed guidelines for preparing your production-ready images, https://jcb.rupress.org/fig-vid-guidelines.

-- Cover images: If you have any striking images related to this story, we would be happy to consider them for inclusion on the journal cover. Submitted images may also be chosen for highlighting on the journal table of contents or JCB homepage carousel. Images should be uploaded as TIFF or EPS files and must be at least 300 dpi resolution.

It is JCB policy that if requested, original data images must be made available to the editors. Failure to provide original images upon request will result in unavoidable delays in publication. Please ensure that you have access to all original data images prior to final submission.

The license to publish form must be signed before your manuscript can be sent to production. A link to the license to publish form will be sent to the corresponding author only. Please take a moment to check your funder requirements before choosing the appropriate license.

Additionally, JCB encourages authors to submit a short video summary of their work. These videos are intended to convey the main messages of the study to a non-specialist, scientific audience. Think of them as an extended version of your abstract, or a short poster presentation. We encourage first authors to present the results to increase their visibility. The videos will be shared on social media to promote your work. For more detailed guidelines and tips on preparing your video, please visit https://rupress.org/jcb/pages/submission-guidelines#videoSummaries.

Thank you for your attention to these final processing requirements. Please revise and format the manuscript and upload materials within 14 days. If you need an extension for whatever reason, please let us know and we can work with you to determine a suitable revision period.

Please contact the journal office with any questions at cellbio@rockefeller.edu.

Thank you for this interesting contribution, we look forward to publishing your paper in Journal of Cell Biology.

Sincerely,

Satyajit Mayor
Monitoring Editor
Journal of Cell Biology

Gabriele Stephan
Scientific Editor
Journal of Cell Biology

Reviewer #1:

The authors have candidly addressed all my comments and suggestions, including additional experiments that support the main message of the paper and the role of these multi-molecular interactions linking CD36 to cortical actin. I also appreciate the extended explanations provided now regarding the conditional co-localization analysis and the respective comparisons with the null hypothesis and the random conditional simulations.
Just a couple of minor comments:
1) Double check the revised version for some minor typos.
2) Remove the reference of Doliwa et al that is still typed after the description of Figure 5.

Reviewer #2:

Although the authors have added some additional experiments and clarifications, I have several remaining technical and biological issues that I think are insufficiently resolved by the authors.

Regarding the radius:
I thank the authors for providing additional clarification regarding the choice of colocalization radius and including the comparison between the different radii. Yet, I still think the rationale for selecting a 2-pixel radius requires further clarification. First, the authors state that a 2-pixel radius provides the "best balance, minimizing false positives and false negatives". Yet, it is unclear how this conclusion was reached. Estimating false positives and negatives requires some form of ground truth, which does not appear to be available here. It would be helpful if the authors could clarify what quantitative measures were used to determine that 2 pixels represents the optimal threshold.
Related to this, the authors conclude from the provided Table that a 1-pixel radius results in insufficient events to calculate the conditional colocalization values. Still, direct pairwise colocalization remains detectable and significant, as also indicated by the authors. An alternative interpretation could therefore be that true three-way colocalization events are rare rather than that the radius is too restrictive. In fact, I do not think that "insufficient data" by itself is a sufficient reason to discard the 1 pixel radius as relevant. I encourage the authors to discuss these aspects more explicitly.
Furthermore, increasing the radius from 1 to 2 pixels expands the area to consider colocalization by a factor of 4 (from a circle with a diameter of 160 nm to 320 nm if I understand correctly). I wonder if such a large area still reflects meaningful molecular proximity or potential interactions. Also, I wonder what the term "cooperativity" means in this respect if the area is this large. It is for example unclear if the three molecules need to be found within the same radius or that an event also scores positive if a target is within 320nm of a condition-positive reference irrespective of where the condition is located (which effectively could be 320 nm in the other direction in that case). Lastly, what is the purpose of performing a sub-pixel localization of the molecules if such a large radius is considered for the colocalization? It would be useful for the authors to eloborate on these aspects further and specifically discuss whether a radius corresponding to this spatial scale remains biologically justified in the context of the system being studied.

Regarding the actin cortex:
While the additional explanation regarding actin organization and TIRF imaging is noted, I feel that my original concern is only partially addressed. I appreciate that, as discussed in Vignaud et al., stress fibers can be mechanically embedded within and coupled to the cortical actin network. Yet, even within this framework, it remains important to distinguish these different actin structures. The cortical actin meshwork directly underlying the plasma membrane is typically considered the main regulator of membrane receptor organization (Chugh and Paluch, 2018; Svitkina, 2021; Mattila et al., 2016). In contrast, the influence of stress fibers on receptor behavior is likely more indirect and mediated through larger-scale mechanical coupling.
In the context of the manuscript, where the authors repeatedly discuss coupling between membrane receptors and cortical actin, I would therefore expect a clearer definition of what is considered "cortical actin." As the authors also note, not all actin, even within the cortex, will necessarily interact with or influence CD36. Figure S1B, Figure S7A and B, but also the newly added Figure S3B & C and likely also the signal from the speckle analysis all display a mixture of actin structures (Cortical actin? Lamellipodia? Stress fibers?). However, the current analyses do not distinguish between these populations, nor is this discussed.
This has two important implications. First, it complicates the interpretation of the results, as regions with high signal from bundled structures (e.g., stress fibers) may disproportionately influence the analysis, even though receptor-actin coupling may depend primarily on the local cortical meshwork and not on filament bundling per se. Second, it makes it difficult to directly link the measured actin intensity to the proposed mechanism of receptor coupling.
I would therefore encourage the authors to discuss these aspects more explicitly in the manuscript. In particular, it would be helpful to clarify what they consider as cortical actin. Related to this, the authors should better explain what is represented by the "periphery" versus "center" of the cell, particularly regarding the actin organization in endothelial cells in their physiological context. Do these regions correspond to distinct actin organizations, membrane environments, or functional states in endothelial cells? How do these spatial differences relate to CD36 biology and its proposed coupling to actin?
Finally, the manuscript would benefit from a schematic or model that integrates the key findings, including cortical actin organization, receptor distribution, caveolae localization, and spatial differences between periphery and center, to provide a clearer overview of the proposed mechanism and its biological implications.

Regarding the conditional colocalization:
I thank the authors for adding the Cohen's d values. Although there is an expected direct correlation between the d values and the p values, Cohen's d is indeed somewhat more informative in quantifying the extent of the effect. Yet, it remains unclear what the reported p-values indicate in Table S1, specifically for the conditional colocalization. The Methods section indicates that "For the conditional colocalization measures (all but p(TwR)), three rank-sum tests were performed to assess significance, namely comparison of each measure to the corresponding nullTR, randC (obtained by randomizing the condition objects, repeated 100 times) and p(TwR). The reported p-value was the maximum (i.e. least significant) of these three tests."
Yet, those mentioned comparisons are all very different and contain different types of information. I am not sure how informative a comparison with nullTR is and a comparison with randC and p(TwR) could result in very different values depending on how the molecules are distributed. More specifically, there are many similar changes observed in the conditional fraction as compared to p(TwR) that have a broad range of p-values or Cohens d values. For example: CD36 WT Edge, Beta1, CD81 increase from 0.10 to 0.24 --> -log10(p-value): 0.48, Cohens d: 0.00 while CD36 WT Edge, Beta1, CD9 increase from 0.13 to 0.24 --> -log10(p-value): 3.03, Cohens d: 1.80. The nonintuitive nature of these numbers makes it very challenging to infer biological conclusions from the data and I would like to know from the authors how it is possible that seemingly similar changes in the colocalization fractions can result in such different p values/effect sizes.

Other remaining comment regarding the annotation of the fractions:
The annotation of the fraction of colocalization remains unclear for me and since the paper is still very technical, it would suggest the following. Currently, it is "p(TwR)", "p(TwR|TwC)", and "p^rs(Tw(RwC))" for the three different conditions. Yet, if I understand correctly, I think it would be more consistent to present them as p(TwR), p(TwR|TwC), and p(TwR|RwC). Or alternatively p(TwR), p((TwC)wR), p(Tw(RwC)). Or is there a fundamental difference between the two conditional fractions that I am overlooking?

Reviewer #3:

Summary: This study investigates the role of the association of CD36 with tetraspanins and integrins on CD36 localization and signalling on cells. Mutating a single residue (G12V) is sufficient to reduce CD36 interactions with several tetraspanins and integrins, transitions CD36 to larger confinement regions, and impairs SFK activation following ligand stimulation. This study relies primarily on high-resolution and single molecule imaging approaches to probe these phenomena. In my initial review I identified some unsupported assumptions made in their model of CD36-actin interactions and some limitations in the data that were used to draw the main conclusion.

The authors have significantly revised the paper in response to my, and the other reviewers, comments. These revisions include several new experiments, including a proximity labeling experiment, which nicely support their original data. These revisions sufficiently address the concerns I had raised in my first review, no new concerns have been raised by the new results, and I agree with the authors interpretation of the new data and how it fits into their study.

2nd Revision - Authors' Response to Reviewers: June 8, 2026

We thank the reviewers for their positive assessment of our revised manuscript and Reviewer 2 for their follow up critiques, which we have strived to fully address in this manuscript revision. Below please find a point-by-point response to the reviews, indicating where and how we address each critique in the manuscript. To aid with the revision assessment, we include with our submission a PDF of the manuscript, with the changes in response to reviewers highlighted in yellow. We believe that our manuscript has become stronger, thank to the added definitions of critical terms, discussion of various complexities, and more detailed explanation of conditional colocalization analysis.

Reviewer #1:

The authors have candidly addressed all my comments and suggestions, including additional experiments that support the main message of the paper and the role of these multi-molecular interactions linking CD36 to cortical actin. I also appreciate the extended explanations provided now regarding the conditional co-localization analysis and the respective comparisons with the null hypothesis and the random conditional simulations.
Just a couple of minor comments:
1) Double check the revised version for some minor typos.
2) Remove the reference of Doliwa et al that is still typed after the description of Figure 5.

We thank the reviewer for their positive assessment of our work and are glad that we have addressed their critiques to their satisfaction. We have done our best to remove all typos; hopefully the revised manuscript does not have any. The Doliwa et al. reference has been removed.

Reviewer #2:

Although the authors have added some additional experiments and clarifications, I have several remaining technical and biological issues that I think are insufficiently resolved by the authors.

We hope that with this revised manuscript we address the reviewer's remaining concerns.

(1) Regarding the radius:
I thank the authors for providing additional clarification regarding the choice of colocalization radius and including the comparison between the different radii. Yet, I still think the rationale for selecting a 2-pixel radius requires further clarification.
First, the authors state that a 2-pixel radius provides the "best balance, minimizing false positives and false negatives". Yet, it is unclear how this conclusion was reached. Estimating false positives and negatives requires some form of ground truth, which does not appear to be available here. It

would be helpful if the authors could clarify what quantitative measures were used to determine that 2 pixels represents the optimal threshold.

We thank the reviewer for their thoughtful critique of our choice of radius and words. Regarding the choice of colocalization radius = 2 pixels, in fact there is a straightforward rationale for this choice, which is that, in the Tetraspeck beads experiments, after registration shift, the bead alignment between channels is 0-2 pixels. This was mentioned in the earlier versions of our manuscript, but not explicitly. Now we write this explicitly to justify the choice of colocalization radius = 2 pixels; please see lines 723-725 in the revised manuscript.

As for the terminology "minimizing false positives and false negatives," the reviewer is correct that we do not have any ground truth in this work to designate something as false positive or negative. Rather, this terminology was based on findings in our earlier work (Vega-Lugo et al. JCB 2022), where we assessed the performance of the conditional colocalization analysis using simulated data as well as using positive and negative control molecule combinations. Please note that this terminology was used only in the response to reviewers document, and not in the manuscript itself. This is the case in the current revised manuscript as well. To avoid any confusion over terminology, we have simplified the justification of the colocalization radius to purely refer to the beads data after registration shift, as mentioned above.

(2) Related to this, the authors conclude from the provided Table that a 1-pixel radius results in insufficient events to calculate the conditional colocalization values. Still, direct pairwise colocalization remains detectable and significant, as also indicated by the authors. An alternative interpretation could therefore be that true three-way colocalization events are rare rather than that the radius is too restrictive. In fact, I do not think that "insufficient data" by itself is a sufficient reason to discard the 1 pixel radius as relevant. I encourage the authors to discuss these aspects more explicitly.

We are afraid that there might be a misunderstanding here. In order to address this point, we repeat the table of colocalization p-values from the 1-pixel, 2-pixel and 3-pixel colocalization analyses that we included in our response to the first round of review.

**Target**	**Reference**	**Condition**	**-Log 10 pValues**								
			pTwR			p_TwR_TwC			p^rs_RwC		
			1 pixel	2 pixel	3 pixel	1 pixel	2 pixel	3 pixel	1 pixel	2 pixel	3 pixel
CD36 WT	β1	CD9	**2.55**	**6.52**	**7.42**	NaN	**3.03**	**5.53**	NaN	**1.97**	1.61
β1	CD36 WT	CD9	**1.68**	**2.20**	**2.03**	NaN	0.30	0.47	NaN	**2.08**	**2.25**
CD36 WT	CD9	β1	**1.73**	**3.01**	**6.40**	NaN	**3.48**	**5.32**	NaN	0.58	0.46

CD9	CD36 WT	β1	**1.63**	**2.20**	**2.67**	NaN	0.02	0.09	NaN	**4.44**	**2.36**
CD9	β1	CD36 WT	**11.19**	**11.19**	**11.2**	NaN	0.25	0.19	NaN	0.95	0.77
β1	CD9	CD36 WT	**10.98**	**11.19**	**10.7**	NaN	1.54	1.66	NaN	1.17	0.79

We would like to clarify that the case of insufficient data in the 1-pixel analysis, indicated by NaN, does not mean that there aren't enough 3-way colocalization events to calculate a p-value. Rather, it means that the subset of target molecules (in the case of p(TwR|TwC)) or the subset of reference molecules (in the case of p^{rs}(Tw(RwC))) colocalized with the condition is too small to perform any subsequent analysis and ask whether that subset has altered colocalization with the reference (in the case of p(TwR|TwC)) or with the target (in the case of p^{rs}(Tw(RwC))) compared to its general population.

Specifically, for our colocalization analysis, a minimum of 20 objects is needed to perform statistical tests and assess significance. If the number of e.g. condition-positive target molecules is less than that (as is the case in the 1-pixel analyses shown in the table), then that subset of target molecules (i.e., condition-positive target molecules) is too small to ask the follow-up question of whether the condition-positive target molecules have enhanced colocalization with the reference. This is what NaN indicates for the conditional measures p(TwR|TwC) and p^{rs}(Tw(RwC)). Note that, at the same time, these low numbers might still correspond to fractions that are significantly higher than expected by change, which would lead to significant pairwise colocalization (p(TwR)).

(3) Furthermore, increasing the radius from 1 to 2 pixels expands the area to consider colocalization by a factor of 4 (from a circle with a diameter of 160 nm to 320 nm if I understand correctly). I wonder if such a large area still reflects meaningful molecular proximity or potential interactions.

As mentioned at Point 1 above, a colocalization radius of 2 pixels (162 nm) was necessary to account for the residual registration shift between channels after channel alignment. As the reviewer rightfully points out, this scale is large for molecular dimensions. Thus, we do not take our colocalization results as indicative of direct, physical interactions; rather, we use the umbrella term "assemblies," which we state could be complexes, clusters, or other PM domains. We elaborate on this point now more in the manuscript, and explicitly write that one reason that the nature of the assemblies is unknown is the spatial scale of colocalization analysis. In addition, to keep all possibilities open, we no longer use the term nanodomain in the revised manuscript, but rather "plasma membrane domain" (which we already used in some places in earlier versions of our manuscript). Please see the revised text on lines 137-144 of the revised manuscript.

Having said that, a colocalization radius of 162 nm is still within the spatial scale of some plasma membrane domains, especially those involving tetraspanins. For example, Schmidt et al. (Sci Rep, 2024; cited in the manuscript) examined the lateral distribution of tetraspanin-enriched assemblies

and showed that these domains can exist at both nanoscale and mesoscale levels. Using STED microscopy, they identified conventional tetraspanin nanodomains in the ~60–100 nm range, and in addition described larger assemblies termed “arranged crowds” spanning ~900 nm neighborhoods. These arranged crowds were defined as irregularly shaped micron-scale regions enriched in multiple tetraspanin nanodomains, i.e. regions in which tetraspanin assemblies are more locally concentrated than outside these domains.

Additionally, another super-resolution study demonstrated that, while clusters composed of a single tetraspanin species may themselves appear randomly distributed, the clusters of different tetraspanins can nevertheless exhibit non-random higher-order spatial organization relative to one another (Zuidscherwoude et al., Sci Rep., 2015). Specifically, it was observed that distinct tetraspanin nanoclusters (~100–120 nm) were frequently positioned in closer proximity to each other than expected by chance.

In addition to the spatial scale of our analysis being within the spatial scale of various plasma membrane domains, we would like to reiterate here that our colocalization analysis builds on multiple earlier works that have themselves shown physical interactions between the molecules in our study. These are cited in our manuscript, including:

- For CD36 with integrins and tetraspanins: (Heit et al., 2013; Huang et al., 2011; Kazerounian et al., 2011; Miao et al., 2001; Primo et al., 2005; Thorne et al., 2000).
- For integrins and tetraspanins with each other: (Berditchevski, 2001; Charrin et al., 2009; Yauch et al., 2000; Zhang et al., 2009).

Finally, the proximity ligation assay experiments performed in response to the previous round of reviews (Fig. S5), as well as those in Saini et al. BioRXiv (https://doi.org/10.1101/2025.07.15.664851; cited in our manuscript), confirmed our colocalization analysis results (those tested via PLA) at a smaller spatial scale.

(4) Also, I wonder what the term "cooperativity" means in this respect if the area is this large. It is for example unclear if the three molecules need to be found within the same radius or that an event also scores positive if a target is within 320nm of a condition-positive reference irrespective of where the condition is located (which effectively could be 320 nm in the other direction in that case).

The term “cooperativity” in our manuscript primarily referred to the (statistical) interdependence of colocalization between objects, in other words that if object X is with Y, then it has a higher chance to be also with Z. To avoid any ambiguity and confusion, we have now removed the term cooperativity from the manuscript. Instead, we use the term interdependence, and state explicitly that this is interdependence in a statistical sense, not in any sense of molecular mechanism. See line 128 for the first use of the term “interdependence.”

Here we would like to clarify however that the maximum distance between objects to be considered colocalized is the colocalization radius (i.e., 160 nm), and not the colocalization diameter (320 nm) as stated by the reviewer. Apart from this misunderstanding, the reviewer is

correct in their understanding that an event scores positive if a target is within the colocalization radius (160 nm) of a condition-positive reference irrespective of where the condition is located, which could be 160 nm in the other direction. Having said that, again our previous use of the term cooperativity and current use of the term interdependence is purely in a statistical sense, which we hope is now clearer in the revised manuscript.

(5) Lastly, what is the purpose of performing a sub-pixel localization of the molecules if such a large radius is considered for the colocalization? It would be useful for the authors to eloborate on these aspects further and specifically discuss whether a radius corresponding to this spatial scale remains biologically justified in the context of the system being studied.

Sub-pixel localization allows us to better correct for registration shifts between channels and allows a more precise calculation of the distance between objects, regardless of the employed colocalization radius. To give a 1D example, suppose one object is at position 3.1 while another object is at position 5.4. Then the distance between the two objects is 5.4-3.1 = 2.3, which is greater than the colocalization radius, and the two objects will not be considered colocalized. Without subpixel localization, the positions would be 3 and 5, resulting in a distance of 5-3 = 2, which would lead to considering the two objects to be colocalized.

As for the biological relevance of the spatial scale of our colocalization analysis, please see our response at Point 3 above.

(6) Regarding the actin cortex:
While the additional explanation regarding actin organization and TIRF imaging is noted, I feel that my original concern is only partially addressed. I appreciate that, as discussed in Vignaud et al., stress fibers can be mechanically embedded within and coupled to the cortical actin network. Yet, even within this framework, it remains important to distinguish these different actin structures. The cortical actin meshwork directly underlying the plasma membrane is typically considered the main regulator of membrane receptor organization (Chugh and Paluch, 2018; Svitkina, 2021; Mattila et al., 2016). In contrast, the influence of stress fibers on receptor behavior is likely more indirect and mediated through larger-scale mechanical coupling.
In the context of the manuscript, where the authors repeatedly discuss coupling between membrane receptors and cortical actin, I would therefore expect a clearer definition of what is considered "cortical actin." As the authors also note, not all actin, even within the cortex, will necessarily interact with or influence CD36. Figure S1B, Figure S7A and B, but also the newly added Figure S3B & C and likely also the signal from the speckle analysis all display a mixture of actin structures (Cortical actin? Lamellipodia? Stress fibers?). However, the current analyses do not distinguish between these populations, nor is this discussed.
This has two important implications. First, it complicates the interpretation of the results, as regions with high signal from bundled structures (e.g., stress fibers) may disproportionately influence the analysis, even though receptor-actin coupling may depend primarily on the local

cortical meshwork and not on filament bundling per se. Second, it makes it difficult to directly link the measured actin intensity to the proposed mechanism of receptor coupling.
I would therefore encourage the authors to discuss these aspects more explicitly in the manuscript. In particular, it would be helpful to clarify what they consider as cortical actin.

We thank the reviewer for highlighting the structurally heterogenous nature of the actin cortex. What comprises the actin cortex depends on the experimental conditions, the cell type being studied, and which cell surface is probed (e.g., ventral vs. dorsal) (Svitkina, 2020). In our model system of well spread endothelial cells on fibronectin-coated coverslips (>24 h), and in the context of ventral TIRFM imaging, we use the term "cortical actin" to collectively refer to the various actin cytoskeletal components visible in our images. This includes the barrier-forming cortical actin meshwork, short dynamic cortical actin filaments interspersed within the meshwork, and actin bundles/stress fibers interconnected with the meshwork, all of which have been implicated in influencing the organization and dynamics of PM constituents in various ways (Gowrishankar et al., 2012; Kusumi et al., 2012; Lehtimaki et al., 2021; Li et al., 2020; Svitkina, 2020; Vignaud et al., 2021). Given the many different definitions of cortical actin, we agree with the reviewer that the term should be well-defined in our manuscript, which we do now in the introduction (see lines 56-61 in the revised manuscript).

Given that the cortical actin that we see in our images is a composite of multiple actin cytoskeleton components, the reviewer is correct that the coupling that we observe between CD36 and actin is a superposition of all of the different components' effects. As the reviewer points out, some components might be truly linked to CD36 while others not; some may have opposing effects. At this stage, our analysis is not able to dissect the actin signals and determine what comes from which component; this requires further developments, probably both experimentally and computationally, that are beyond the scope of this work. Here we would like to highlight that, despite this complexity, reproducible relationships emerge between actin cytoskeleton dynamics and CD36 organization and mobility in our analyses. Also, our SMI-FSM assay has the sensitivity to detect changes in these relationships when CD36 is mutated (G12V), allowing us to gain new insight into the molecules that link CD36 to actin and the role that this link plays in CD36 organization and signaling, even if the nature of that link is not yet fully dissected and understood. We discuss these issues now explicitly in the manuscript; please see lines 235-239.

(7) Related to this, the authors should better explain what is represented by the "periphery" versus "center" of the cell, particularly regarding the actin organization in endothelial cells in their physiological context. Do these regions correspond to distinct actin organizations, membrane environments, or functional states in endothelial cells? How do these spatial differences relate to CD36 biology and its proposed coupling to actin?

Actin organization in the periphery and center is indeed distinct. In our system, the peripheral region corresponds to the lamellipodium and part of the adjacent lamella behind the free cell edge, a region characterized by higher actin density and different actin dynamics compared to the cell

center. As this cell edge is a free edge – without contact with neighbors – the peripheral region right behind it resembles the leading edge of spreading or migrating cells, e.g., tip endothelial cells in the context of angiogenesis. This was stated in the previous version of the manuscript, but in a scattered manner (in multiple places). Now we have consolidated this information into one place (lines 113-120), to clearly define the two regions in our study.

Beyond actin organization, our study is helping define the differences between these two regions in terms of molecular organization and signaling. To name some, we observe differences in molecule density (Fig. S1) (in addition to differences in actin organization and dynamics (Fig. S1A and Fig. S4C)), in molecular interactions (Fig. 1), in CD36-actin coupling (Fig. 2A, B), in CD36 organization (e.g., localization in caveolae; Fig. 5A, B), and in signaling (Fig. 5C-E). Interestingly, the increased SFK signaling observed at the WT periphery is reminiscent of localized Fyn activation reported at the leading edge of migrating cells (as mentioned in the manuscript, lines 459-461). This observation is particularly relevant in endothelial cells, where Fyn contributes to signaling pathways regulating migration and apoptosis. It is important to remember, however, that the endothelial cells in our experiments were for the most part not exposed to stimuli. Therefore, while the periphery and center clearly represent distinct cortical actin and signaling environments, extensive discussion of the differences in these regions would extend beyond the scope of the present study.

(8) Finally, the manuscript would benefit from a schematic or model that integrates the key findings, including cortical actin organization, receptor distribution, caveolae localization, and spatial differences between periphery and center, to provide a clearer overview of the proposed mechanism and its biological implications.

We thank the reviewer for this suggestion, which we have now implemented. We have added a new Fig. 6, which shows a model/schematic of our main findings.

(9) Regarding the conditional colocalization:
I thank the authors for adding the Cohen's d values. Although there is an expected direct correlation between the d values and the p values, Cohen's d is indeed somewhat more informative in quantifying the extent of the effect. Yet, it remains unclear what the reported p-values indicate in Table S1, specifically for the conditional colocalization. The Methods section indicates that "For the conditional colocalization measures (all but p(TwR)), three rank-sum tests were performed to assess significance, namely comparison of each measure to the corresponding nullTR, randC (obtained by randomizing the condition objects, repeated 100 times) and p(TwR). The reported p-value was the maximum (i.e. least significant) of these three tests."
Yet, those mentioned comparisons are all very different and contain different types of information. I am not sure how informative a comparison with nullTR is and a comparison with randC and p(TwR) could result in very different values depending on how the molecules are distributed. More specifically, there are many similar changes observed in the conditional fraction as compared to p(TwR) that have a broad range of p-values or Cohens d values. For example: CD36 WT Edge, Beta1,

CD81 increase from 0.10 to 0.24 --> -log10(p-value): 0.48, Cohens d: 0.00 while CD36 WT Edge, Beta1, CD9 increase from 0.13 to 0.24 --> -log10(p-value): 3.03, Cohens d: 1.80. The nonintuitive nature of these numbers makes it very challenging to infer biological conclusions from the data and I would like to know from the authors how it is possible that seemingly similar changes in the colocalization fractions can result in such different p values/effect sizes.

The reviewer is correct that the three comparisons (with nullTR, with randC, and with p(TwR)) contain different information. We explain this now in more detail in the Materials and Methods section "Statistical testing for colocalization analysis" (lines 730-864). All three tests are essential and informative in different ways, which we hope will be now clearer with the extended explanation in the Materials and Methods.

As for the specific examples brought up by the reviewer, indeed it can happen that a seemingly similar change from p(TwR) to p(TwR|TwC) yields different p-values, because the reported p-value is not only from comparing p(TwR|TwC) to p(TwR), but also from comparing it to the corresponding nullTR and randC. In this specific example, for the CD81 case, the three p-values for the 3 comparisons are 0.019 (comparison to p(TwR)), 0.33 (comparison to nullTR), and 0.06 (comparison to randC). For the CD9 case, the three p-values are 0.0002 (comparison to p(TwR)), 0.0006 (comparison to nullTR), and 0.0009 (comparison to randC). The least significant p-values in the two cases are 0.33 for the CD81 case and 0.0009 for the CD9 case. These yield the cited -log10(p-values) of 0.48 (CD81 case) and 3.03 (CD9 case). Please note that a statistical test's outcome does not only depend on the difference in magnitude between the two quantities being compared, but also the sample size.

(10) Other remaining comment regarding the annotation of the fractions:
The annotation of the fraction of colocalization remains unclear for me and since the paper is still very technical, it would suggest the following. Currently, it is "p(TwR)", "p(TwR|TwC)", and "p^rs(Tw(RwC))" for the three different conditions. Yet, if I understand correctly, I think it would be more consistent to present them as p(TwR), p(TwR|TwC), and p(TwR|RwC). Or alternatively p(TwR), p((TwC)wR), p(Tw(RwC)). Or is there a fundamental difference between the two conditional fractions that I am overlooking?

We apologize that we did not fully clarify the fundamental difference between p(TwR|TwC) (which is truly a conditional probability, i.e., a fraction) and p^{rs}(Tw(RwC)) (which is actually a rescaled probability, and is not a fraction). We explain this now in more detail in the Materials and Methods section "Statistical testing for colocalization analysis" (lines 765-771). We hope that this explanation clarifies what these measures are and how they are different, thus clarifying our terminology/measure names.

Reviewer #3:

Summary: This study investigates the role of the association of CD36 with tetraspanins and

integrins on CD36 localization and signalling on cells. Mutating a single residue (G12V) is sufficient to reduce CD36 interactions with several tetraspanins and integrins, transitions CD36 to larger confinement regions, and impairs SFK activation following ligand stimulation. This study relies primarily on high-resolution and single molecule imaging approaches to probe these phenomena. In my initial review I identified some unsupported assumptions made in their model of CD36-actin interactions and some limitations in the data that were used to draw the main conclusion.

The authors have significantly revised the paper in response to my, and the other reviewers, comments. These revisions include several new experiments, including a proximity labeling experiment, which nicely support their original data. These revisions sufficiently address the concerns I had raised in my first review, no new concerns have been raised by the new results, and I agree with the authors interpretation of the new data and how it fits into their study.

We thank the reviewer for their positive assessment of our work and are glad that we have addressed their critiques to their satisfaction.

References

Berditchevski, F. 2001. Complexes of tetraspanins with integrins: more than meets the eye. *Journal of Cell Science*. 114:4143-4151.

Charrin, S., F. le Naour, O. Silvie, P.E. Milhiet, C. Boucheix, and E. Rubinstein. 2009. Lateral organization of membrane proteins: tetraspanins spin their web. *Biochem J*. 420:133-154.

Gowrishankar, K., S. Ghosh, S. Saha, R. C, S. Mayor, and M. Rao. 2012. Active remodeling of cortical actin regulates spatiotemporal organization of cell surface molecules. *Cell*. 149:1353-1367.

Heit, B., H. Kim, G. Cosio, D. Castano, R. Collins, C.A. Lowell, K.C. Kain, W.S. Trimble, and S. Grinstein. 2013. Multimolecular Signaling Complexes Enable Syk-Mediated Signaling of CD36 Internalization. *Developmental Cell*. 24:372-383.

Huang, W., M. Febbraio, and R.L. Silverstein. 2011. CD9 tetraspanin interacts with CD36 on the surface of macrophages: a possible regulatory influence on uptake of oxidized low density lipoprotein. *PLoS One*. 6:e29092.

Kazerounian, S., M. Duquette, M.A. Reyes, J.T. Lawler, K.L. Song, C. Perruzzi, L. Primo, R. Khosravi-Far, F. Bussolino, I. Rabinovitz, and J. Lawler. 2011. Priming of the vascular endothelial growth factor signaling pathway by thrombospondin-1, CD36, and spleen tyrosine kinase. *Blood*. 117:4658-4666.

Kusumi, A., T.K. Fujiwara, R. Chadda, M. Xie, T.A. Tsunoyama, Z. Kalay, R.S. Kasai, and K.G. Suzuki. 2012. Dynamic organizing principles of the plasma membrane that regulate signal transduction: commemorating the fortieth anniversary of Singer and Nicolson's fluid-mosaic model. *Annu Rev Cell Dev Biol*. 28:215-250.

Lehtimaki, J.I., E.K. Rajakyla, S. Tojkander, and P. Lappalainen. 2021. Generation of stress fibers through myosin-driven reorganization of the actin cortex. *Elife*. 10.

Li, J.H., P. Santos-Otte, B. Au, J. Rentsch, S. Block, and H. Ewers. 2020. Directed manipulation of membrane proteins by fluorescent magnetic nanoparticles. *Nature Communications*. 11:4259.

Miao, W.M., E. Vasile, W.S. Lane, and J. Lawler. 2001. CD36 associates with CD9 and integrins on human blood platelets. *Blood*. 97:1689-1696.

Primo, L., C. Ferrandi, C. Roca, S. Marchio, L. di Blasio, M. Alessio, and F. Bussolino. 2005. Identification of CD36 molecular features required for its in vitro angiostatic activity. *Faseb Journal*. 19:1713-+.

Svitkina, T.M. 2020. Actin Cell Cortex: Structure and Molecular Organization. *Trends Cell Biol*. 30:556-565.

Thorne, R.F., J.F. Marshall, D.R. Shafren, P.G. Gibson, I.R. Hart, and G.F. Burns. 2000. The integrins alpha(3)beta(1) and alpha(6)beta(1) physically and functionally associate with CD36 in human melanoma cells - Requirement for the extracellular domain of CD36. *Journal of Biological Chemistry*. 275:35264-35275.

Vignaud, T., C. Copos, C. Leterrier, M. Toro-Nahuelpan, Q. Tseng, J. Mahamid, L. Blanchoin, A. Mogilner, M. Thery, and L. Kurzawa. 2021. Stress fibres are embedded in a contractile cortical network. *Nat Mater*. 20:410-420.

Yauch, R.L., A.R. Kazarov, B. Desai, R.T. Lee, and M.E. Hemler. 2000. Direct extracellular contact between integrin alpha(3)beta(1) and TM4SF protein CD151. *Journal of Biological Chemistry*. 275:9230-9238.

Zhang, F., J. Kotha, L.K. Jennings, and X.A. Zhang. 2009. Tetraspanins and vascular functions. *Cardiovasc Res*. 83:7-15.
